# ﻿New Species of *Virola* (Myristicaceae) from South America

**DOI:** 10.3897/phytokeys.197.81367

**Published:** 2022-05-30

**Authors:** Daniel Santamaría-Aguilar, Laura P. Lagomarsino

**Affiliations:** 1 Shirley C. Tucker Herbarium, Department of Biological Sciences, Louisiana State University, 103 Life Sciences Building, Baton Rouge, Louisiana 70803-1705, USA Louisiana State University Baton Rouge United States of America; 2 Missouri Botanical Garden, St. Louis, Missouri, USA Missouri Botanical Garden St. Louis United States of America

**Keywords:** Brazil, Colombia, Ecuador, Herbarium, Magnoliales, Neotropics, nutmeg, Peru, Taxonomy

## Abstract

With about 70 species *Virola*, is the largest genus of Myristicaceae in the Neotropics, the genus ranked in the top ten genera of abundance across Amazonia. Ten new species are proposed in this striking genus, which are described based on morphology, and are illustrated. The new species were discovered thanks to herbarium specimens collected mainly in the 1980s and 1990s when field documentations were more active. The new species come from Colombia (*V.calimensis***sp. nov.**, *V.cogolloi***sp. nov.**, *V.excisa***sp. nov.**, *V.tuckerae***sp. nov.**), Ecuador (*V.alvaroperezii***sp. nov.**, *V.bombuscaroensis***sp. nov.**, *V.calimensis*, *V.excisa*, *V.yasuniana***sp. nov.**), Peru (*V.aguarunana***sp. nov.**, *V.cumala***sp. nov.**, *V.excisa*, *V.parkeri***sp. nov.**), and Brazil (*V.excisa*, *V.yasuniana*). Additionally, a lectotype is designated for *V.macrocarpa*, a name used to identify some specimens of the new species here described, and *V.kwatae* is reported for the first time for Brazil. We provide a comparation table between the new species and the species that is morphologically close to it, a preliminary list of species for the genus, and notes of how the new species were treated in floras, checklists, or collections that need more study and herbarium specimens.

## ﻿Introduction

**Figure 1.**
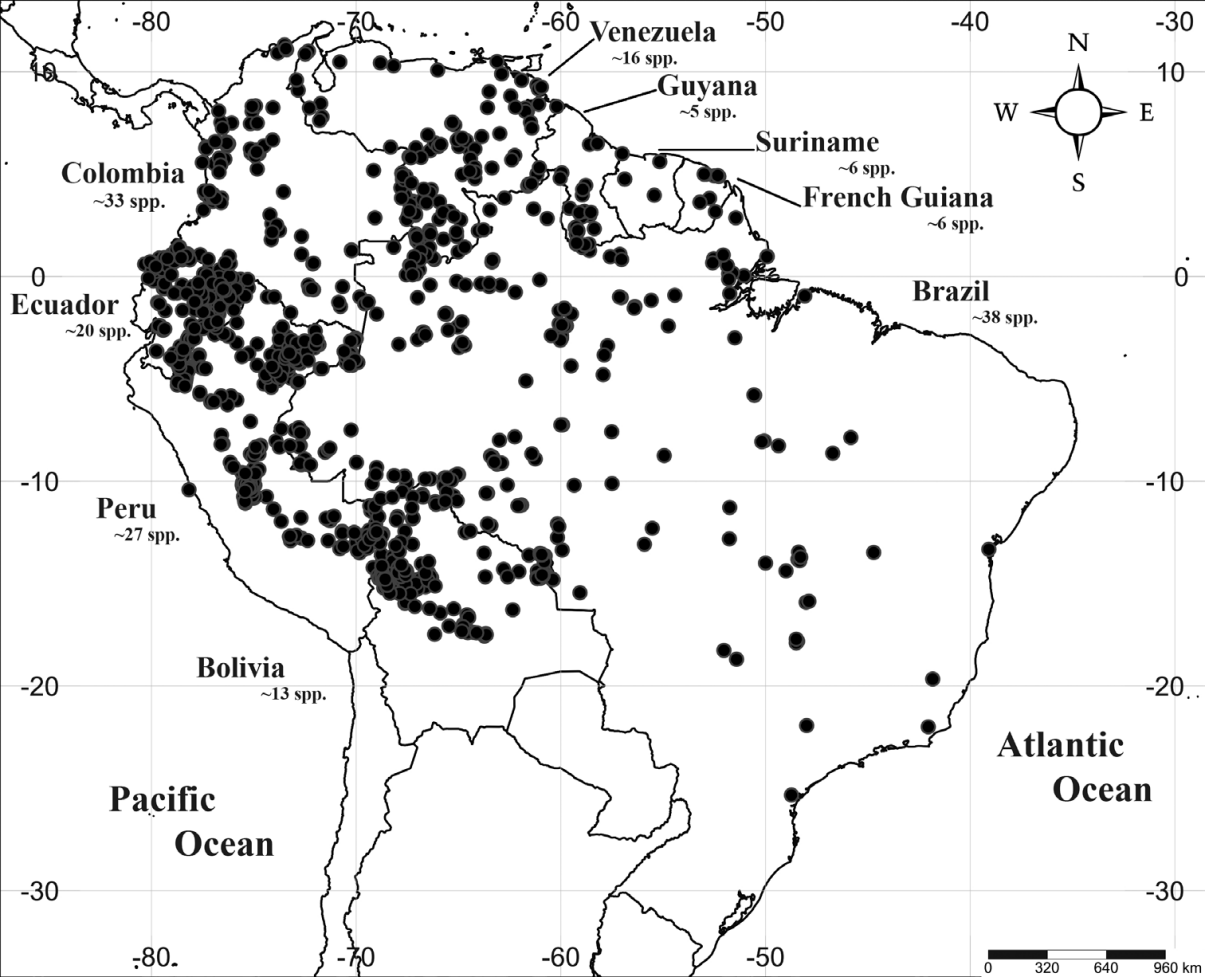
Geographic distribution of *Virola* in South America with number of species per country.

*Virola* Aubl. (Myristicaceae) is an ecologically and economically important genus of trees in the nutmeg family, Myristicaceae within the order Magnoliales (APG IV 2016). It is the fourth largest genus in Myristicaceae, a pantropical family with 21 genera and nearly 500 species; only three Asian genera, *Horsfieldia* Willd., *Knema* Lour., and *Myristica* Gronov., are larger. *Virola* occurs across the wet Neotropics, where it is distributed from Mexico to southern Brazil and in the West Indies [i.e. *V.surinamensis* (Rol. ex Rottb.) Warb.], though it is notably absent from El Salvador (Smith and Wodehouse 1938; Rodrigues 1980; Acevedo-Rodríguez and Strong 2012; Santamaría-Aguilar et al. 2019). Across *Virola*’s *ca.* 70 species (including those described here), *ca.* 55 occur in South America (Fig. 1) and 15 in Central America, with just two widespread species [i.e., *V.elongata* (Benth.) Warb., and *V.sebifera* Aubl.] occurring in both (Table 1). The species richness of *Virola* is highest in Amazonia, where it is ranked in the top ten most abundant genera with 35 species (Cardoso et al. 2017). Like other genera that are most species-rich and abundant in Amazonian forests including *Protium* Burm. f. (Burseraceae) (Daly 2020; Daly et al. 2020), a smaller number of species are confined to montane forests, and an even smaller number of species occur in dry areas such as Brazilian (Rodrigues 1980). A few widespread species, including *V.elongata* and *V.sebifera*, can be found across different forest types (Aymard et al. 2020).

**Table 1.** List of species of *Virola* in Central and South America, and Antilles. Region: C = Central America; S = South America; A = Antilles; M = Mexico. For the number of species per country in South America see Fig. 1. References next to a country correspond to species for which we have not seen a herbarium specimen for that country.

Species	Country of original material	Region	Distributions
*V.aequatorialis* Muriel & Balslev. Nordic J. Bot. 20(4): 443 (–445) (2000).	Ecuador	S	Ecuador.
*V.aguarunana* D. Santam.	Peru	S	Peru.
*V.albidiflora* Ducke. J. Wash. Acad. Sci. 26: 259 (1936).	Brazil	S	Colombia (Gradstein 2016), Ecuador, Peru (Vásquez M. et al. 2018), Brazil.
*V.allenii* D. Santam. & Aguilar. PhytoKeys 134: 14 (2019).	Costa Rica	C	Costa Rica.
*V.alvaroperezii* D. Santam.	Ecuador	S	Ecuador.
*V.amistadensis* D. Santam. PhytoKeys 134: 24 (2019).	Panama	C	Costa Rica, Panama.
*V.bombuscaroensis* D. Santam.	Ecuador	S	Ecuador.
*V.caducifolia* W. A. Rodrigues. Acta Amazonica 7: 459 (–462) (1977).	Brazil	S	Colombia (Gradstein 2016), Peru (Vásquez M. et al. 2018), Brazil.
*V.calimensis* D. Santam.	Colombia	S	Colombia, Ecuador
*V.calophylla* (Spruce) Warb. Nova Acta Acad. Caes. Leop.-Carol. German. Nat. Cur. 68: 231 (1897).	Venezuela	S	Colombia, Ecuador, Peru, Bolivia, Brazil, Venezuela, Guyana, Suriname.
*V.calophylloidea* Markgr. Repert. Spec. Nov. Regni Veg. 19: 24 (1923).	Brazil	S	Colombia (Aymard C. et al. 2020), Brazil.
*V.carinata* (Benth.) Warb. Nova Acta Acad. Caes. Leop.-Carol. German. Nat. Cur. 68: 222 (1897). (1897).	Brazil	S	Colombia (Gradstein 2016), Peru (Vásquez M. et al. 2018), Bolivia (Achá & Liesner 2014), Brazil, Venezuela (Aymard C. et al. 2020).
*V.chrysocarpa* D. Santam. & Aguilar. PhytoKeys 134: 28 (2019).	Costa Rica	C	Costa Rica, Panama.
*V.coelhoi* W. A. Rodrigues. Acta Amazonica 7: 462 (–464) (1977).	Brazil	S	Colombia, Peru, Brazil
*V.cogolloi* D. Santam.	Colombia	S	Colombia.
*V.crebrinervia* Ducke. J. Wash. Acad. Sci. 26: 260 (1936).	Brazil	S	Brazil.
*V.cumala* D. Santam.	Peru	S	Peru.
*V.decorticans* Ducke. J. Wash. Acad. Sci. 26: 262 (1936).	Brazil	S	Colombia (Gradstein 2016), Peru, Bolivia (Achá & Liesner 2014), Brazil.
*V.divergens* Ducke. Wash. Acad. Sci. 26: 255 (1936).	Brazil	S	Colombia (Gradstein 2016), Ecuador (Jaramillo et al. 2004), Peru, Brazil.
*V.dixonii* Little. Phytologia 19: 255 (1970).	Ecuador	S	Colombia, Ecuador.
*V.duckei* A. C. Sm. Brittonia 2: 487 (1938).	Brazil	S	Colombia, Ecuador, Peru, Bolivia (Achá & Liesner 2014), Brazil, Venezuela (Aymard C. et al. 2007).
*V.elongata* (Benth.) Warb. Ber. Deutsch. Bot. Ges.13: 89 (1895). The use of this name should be evaluated in detail, and perhaps some names put in synonymy should be resurrected.		C, S	Panama, Colombia, Ecuador, Peru, Bolivia, Brazil, Venezuela, Guyana, French Guiana, Suriname.
*V.excisa* D. Santam.	Ecuador	S	Colombia, Ecuador, Peru, Brazil.
*V.flexuosa* A. C. Sm. Brittonia 2: 151 (1936).	Brazil	S	Colombia, Ecuador, Peru, Bolivia, Brazil.
*V.fosteri* D. Santam. PhytoKeys 134: 35(2019).	Panama	C	Costa Rica, Panama.
*V.gardneri* (A. DC.) Warb. Nova Acta Acad. Caes. Leop.-Carol. German. Nat. Cur. 68: 192 (1897).	Brazil	S	Brazil.
*V.guatemalensis* (Hemsl.) Warb. Nova Acta Acad. Caes. Leop.-Carol. German. Nat. Cur. 68: 220 (1897).	Guatemala	C, M	Mexico, Guatemala, Honduras.
*V.guggenheimii* W. A. Rodrigues. Acta Amazonica 7: 464 (–467) (1977).	Brazil	S	Brazil.
*V.koschnyi* Warb. Repert. Spec. Nov. Regni Veg. 1: 71 (1905).	Costa Rica	C, M	Mexico, Guatemala, Honduras, Belize, Nicaragua, Costa Rica, Panama.
*V.kwatae* Sabatier. Adansonia ser. 3, 19: 273 (1997).	French Guiana	S	Brazil, French Guiana.
*V.laevigata* Standl. Publ. Field Mus. Nat. Hist., Bot. Ser. 4: 209 (1929).	Panama	C	Costa Rica, Panama.
*V.loretensis* A. C. Sm. Bull. Torrey Bot. Club 58: 95 (1931).	Peru	C	Colombia, Peru, Bolivia, Brazil, (Achá & Liesner 2014)
*V.macrocarpa* A. C. Sm. Brittonia 2: 476 (1938).	Colombia	S	Colombia.
*V.malmei* A. C. Sm. Brittonia 2: 496 (1938).	Brazil	S	Brazil.
*V.marleneae* W. A. Rodrigues. Acta Amazonica 7: 467 (–469) (1977).	Brazil	S	Colombia, Peru, Brazil.
*V.megacarpa* A. H. Gentry. Ann. Missouri Bot. Gard. 62: 474 (1975).	Panama	C	Panama.
*V.michelii* Heckel. Ann. Inst. Bot.-Géol. Colon. Marseille 6: 118 (1898).	French Guiana	S	Colombia (Gradstein 2016), Bolivia (Achá & Liesner 2014), Brazil, Venezuela, Guyana, French Guiana, Suriname.
*V.micrantha* A. C. Sm. J. Wash. Acad. Sci. 43: 203 (1953).	Colombia	S	Colombia, Venezuela.
*V.minutiflora* Ducke. J. Wash. Acad. Sci. 26: 259 (1936).	Brazil	S	Peru (Vásquez M. et al. 2018), Brazil, Venezuela (Rodrigues et al. 2001).
*V.mollissima* (Poepp. ex A. DC.) Warb. Nova Acta Acad. Caes. Leop.-Carol. German. Nat. Cur. 68: 167 (1897).	Peru	S	Colombia, Ecuador (Jaramillo et al. 2004), Peru, Brazil.
*V.montana* D. Santam. PhytoKeys 134: 52 (2019).	Costa Rica	C	Costa Rica, Panama.
*V.multicostata* Ducke. J. Wash. Acad. Sci. 26: 261 (1936).	Brazil	S	Colombia (Gradstein 2016), Peru, Brazil, French Guiana (Aymard et al. 2007).
*V.multiflora* (Standl.) A. C. Sm. Brittonia 2: 499 (1938).	Belize	C	Honduras, Belize, Nicaragua, Costa Rica, Panama.
*V.multinervia* Ducke. J. Wash. Acad. Sci. 26: 261 (1936).	Brazil	S	Colombia (Gradstein 2016), Ecuador (Jaramillo et al. 2004), Peru (Vásquez M. et al. 2018), Bolivia (Achá & Liesner 2014), Brazil, Venezuela. (Rodrigues 2008).
*V.nobilis* A. C. Sm. Brittonia 2: 490 (1938).	Panama	C	Costa Rica (cf.), Panama.
*V.obovata* Ducke. Bol. Tecn. Inst. Agron. N. no. 4: 12 (1945).	Brazil	S	Colombia, Peru, Brazil.
*V.officinalis* Warb. Nova Acta Acad. Caes. Leop.-Carol. German. Nat. Cur. 68: 228 (1897).	Brazil	S	Brazil.
*V.otobifolia* D. Santam. PhytoKeys 134: 63 (2019).	Panama	C	Panama.
*V.parkeri* D. Santam. & Lagom.	Peru	S	Peru.
*V.parvifolia* Ducke. J. Wash. Acad. Sci. 26: 264 (1936).	Brazil	S	Colombia (Gradstein 2016), Brazil, Venezuela.
*V.parvusligna* Vásquez & L. Valenz. J. Pl. Sci. 10: 28 (2022).	Peru	S	Peru.
*V.pavonis* (A. DC.) A. C. Sm. Brittonia 2: 504 (1938).	Peru	S	Colombia, Ecuador, Peru, Bolivia (Achá & Liesner 2014), Brazil, Venezuela.
*V.peruviana* (A. DC.) Warb. Nova Acta Acad. Caes. Leop.-Carol. German. Nat. Cur. 68: 188 (1897).	Peru	S	Colombia, Ecuador, Peru, Bolivia, Brazil.
*V.polyneura* W.A.Rodrigues. Acta Amazonica 7: 469 (–471) (1977).	Brazil	S	Colombia (Gradstein 2016), Brazil.
*V.pseudosebifera* Vásquez & Soto-Shareva. Q’Euña 10: 8–11 (2019) [2020].	Peru	S	Peru.
*V.reidii* Little. Phytologia 19: 258 (1970).	Ecuador	S	Colombia, Ecuador.
*V.rugulosa* (Spruce) Warb. Nova Acta Acad. Caes. Leop.-Carol. German. Nat. Cur. 68: 227 (1897).	Brazil	S	Brazil, Venezuela.
*V.sanguinea* D. Santam. J. Bot. Res. Inst. Texas 15(2): 343 (2021).	Honduras	C	Honduras.
*V.schultesii* A. C. Sm. Amer. J. Bot. 43: 575 (1956).	Colombia	S	Colombia, Venezuela.
*V.sebifera* Aubl. Pl. Guian. 2: 904 (1775).	French Guiana	C, S	Honduras, Nicaragua, Costa Rica, Panama, Colombia, Ecuador, Peru, Bolivia, Brazil, Venezuela, Guyana, French Guiana, Suriname.
*V.sessilis* (A. DC.) Warb. Nova Acta Acad. Caes. Leop.-Carol. German. Nat. Cur. 68: 190 (1897).	Brazil	S	Brazil.
*V.steyermarkii* W. A. Rodrigues. Ann. Missouri Bot. Gard. 76: 1164 (1989).	Venezuela	S	Venezuela.
*V.subsessilis* (Benth.) Warb. Nova Acta Acad. Caes. Leop.-Carol. German. Nat. Cur. 68: 191 (1897).	Brazil	S	Brazil.
*V.surinamensis* (Rol. ex Rottb.) Warb. Nova Acta Acad. Caes. Leop.-Carol. German. Nat. Cur. 68: 208 (1897).	Suriname	S, A	Colombia, Ecuador, Peru, Bolivia, Brazil, Venezuela, French Guiana, Guyana, Suriname, Antilles.
*V.tuckerae* D. Santam. & Lagom.	Colombia	S	Colombia.
*V.urbaniana* Warb. Nova Acta Acad. Caes. Leop.-Carol. German. Nat. Cur. 68: 168 (1897).	Brazil	S	Brazil.
*V.venosa* (Benth.) Warb. Nova Acta Acad. Caes. Leop.-Carol. German. Nat. Cur. 68: 224 (1897).	Brazil	S	Brazil, Venezuela, Suriname.
*V.weberbaueri* Markgr. Notizbl. Bot. Gart. Berlin-Dahlem 9: 965 (1926).	Peru	S	Peru.
*V.yasuniana* D. Santam.	Ecuador	S	Ecuador, Brazil.

**Figure 2.**
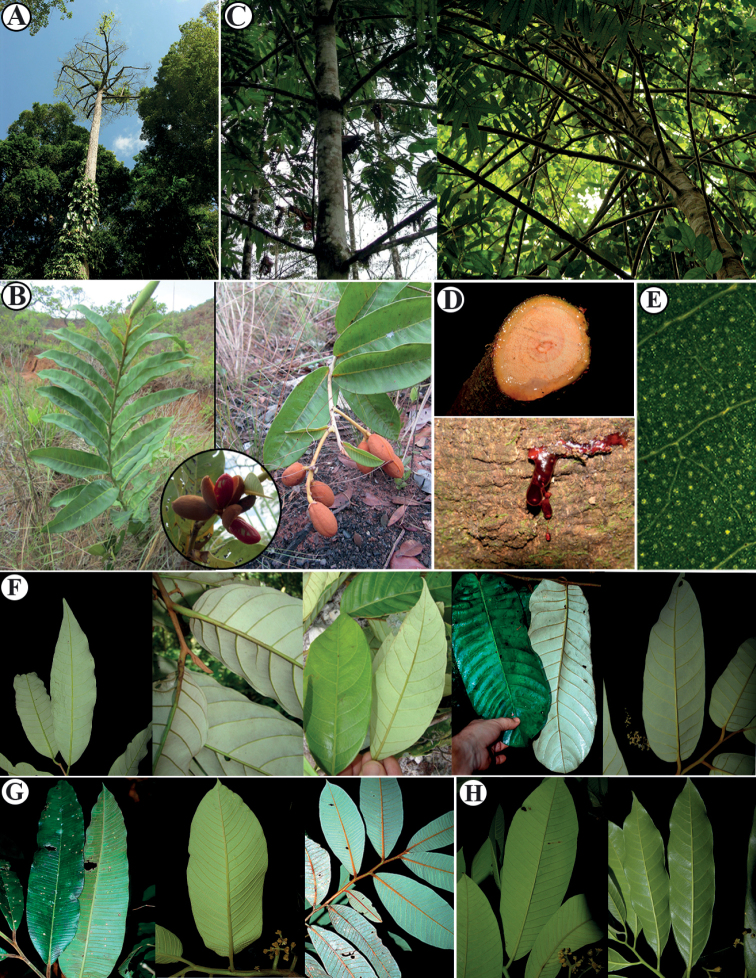
Morphological characters of *Virola***A** tree habitat of *V.chrysocarpa***B** shrubby habitat and fruits of *V.sessilis***C** myristicaceous branching **D** sap on branches (above) and truck (below) **E** leaf punctations **F–H** leaf venations, which correspond to groups Calophyllae, Sebiferae (line **F**; from left to right *V.allenii*, *Virola* spp., *V.calophylla*, *V.sebifera*), Rugolosae (line G; *Virola* spp.) and Surinamenses (line **H**; from left to right V.cf.nobilis, *V.laevigata*) which correspond to groups of Smith and Wodehouse 1938. Photos by Reinaldo Aguilar (**A, D, E, F** [first and last photo], **G**-right, **H**), Denise Barbosa Silva (**B**), Christopher Davidson (line **F**, second and third photo from left to right), Robin Foster (line **F**, fourth from left to right), **G**-left), and Robbin Moran (**C**).

**Figure 3.**
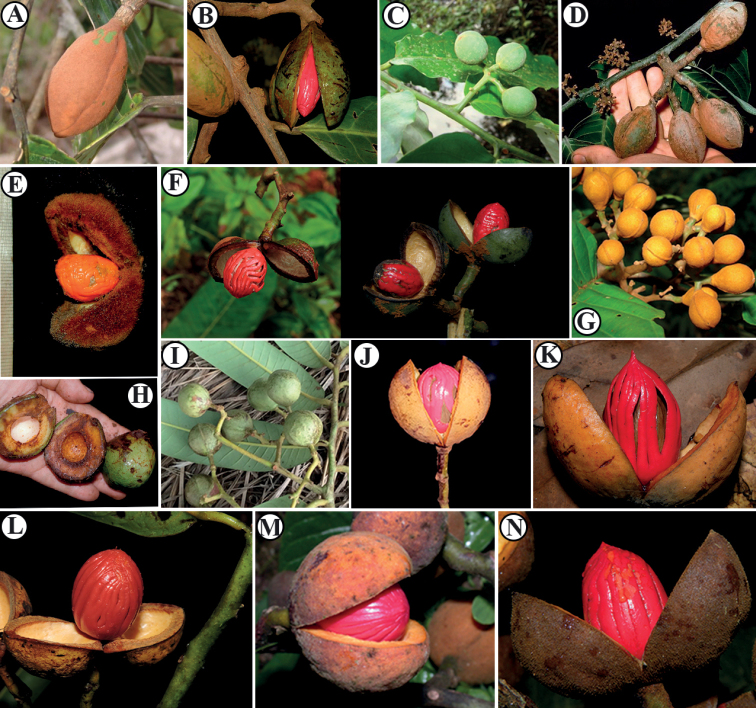
Fruit morphology of *Virola*, note shape, pericarp, indument, and arils. Also see the green color of pericarp, and the minutely pubescent, falling easily as dust (**A–D, F**), and the yellow pericarp (**J–M**) **A***Virola* sp **B***V.allenii***C***V.elongata***D***V.otobifolia***E***Virola* sp **F***V.sebifera***G***Virola* sp **H***Virola* sp **I***V.surinamensis***J***V.fosteri***K**V.cf.nobilis**L***V.laevigata***M***V.koschnyi***N***V.chrysocarpa*. Photos by Reinaldo Aguilar (**B, F, K, L, M, N**), Christopher Davidson (**A, C, G**), Robin Foster (**E, H**), Alwyn H. Gentry (**D**), Manuel Morales (**J**), Luiz O. A. Teixeira (**I**).

*Virola* is morphologically distinctive on account of its myristicaceous tree architecture model known as “Massart” (Hallé et al. 1978) and sometimes referred to as myristicaceous branching, red exudate, pubescence of dentritic or stellate trichomes on under surface (Aymard et al. 2020), and dehiscent fruit which splits to reveal a seed covered by a brightly colored, usually lacinate aril (Figs 2, 3). While *Virola* species tend to be medium to large forest trees, two species [i.e., *V.sessilis* (A. DC.) Warb., *V.subsessilis* (Benth.) Warb.] have an unusually small stature (about 1 to 3 m tall; Fig. 2B). The genus is further characterized by simple, alternate, distichous leaves borne on plagiotropic branches that occasionally have pellucid punctuation (Fig. 2E–G); paniculate staminate inflorescences; ebracteolate, unisexual flowers; a compound androecium with 3 (4–6) anthers fused into a column; and dehiscent fruits that open by two valves that are usually green [e.g., *V.elongata*, *V.obovata* Ducke, *V.sebifera*, and the new species described here] (Fig. 3A–D, F) or yellow [e.g., *V.fosteri* D. Santam., *V.laevigata* Standl., *V.multiflora* (Standl.) A. C. Sm., *V.nobilis* A. C. Sm.] when ripe (Fig. 3J–M), each with a single, arillate seed (Rodrigues 1980; de Wilde 1991; Gentry 1993; Kühn and Kubitzki 1993) (Fig. 3). Among Neotropical Myristicaceae, *Virola* can be vegetatively identified by the combination of its characteristic tree architecture, colored exudate in cut bark or branches, stellate or dendritic trichomes that are often found on young branches, and petioles and leaf blades which display size variation within an individual (see Braga 1992).

*Virola* is important to indigenous and rural communities for its several ethnobotanical attributes. It can be used as medicine to treat malaria, asthma, rheumatism, tumors of the joints, intestinal worms, skin diseases, erysipelas, hemorrhoids, bucal ulcerations, leishmaniasis, and halitosis; while its wood is used both for construction and paper pulp manufacturing, (Rodrigues 1972, 1980; Lourerio et al. 1989; Plotkin and Schultes 1990; Milliken 1997; Rodrigues et al. 2001). Resin from several species, including *V.calophylla* (Spruce) Warb. and *V.calophylloidea* Markgr., are potent hallucinogens (Schultes 1954). These hallucinogens, commonly ingested as a snuff known as *yakee* and *yato* in Colombia and *paricá*, *kawabó*, *epena* or *nyakwana* in Brazil (Schultes 1969, 1978; Aymard et al. 2020), are central to spiritual ceremonies of various indigenous communities in South America. *Virola* is likely also the source of the powerful hallucinogenic snuff *hakudufha* made by the Yekwana tribe in the upper Orinoco River and other tribes (Schultes 1954). For the elaboration of these snuffs, only the resin is used, or they mix it with the ashes of the leaves of *Justiciapectoralis* Jacq. (Acanthaceae), or ashes of the outer bark of *Duguetialepidota* (Miq.) Pulle (Annonaceae), *Elizabethaleiogyne* Ducke (Fabaceae), or *Eschweileraitayensis* R. Knuth (Lecythidaceae); the preparation of snuff varies among different indigenous communities (Schultes 1976; Schultes and Raffauf 1994; Milliken et al. 1999).

The genus has also been well-studied by botanists and ecologists as it is a tractable system for studying seed dispersal (e.g., Howe and Vande Kerckhove 1980; Howe 1981, 1993; Queenborough et al. 2007; Ratiarison and Forget 2013; Moreira et al. 2017).

The last complete taxonomic revision of *Virola* was published over 80 years ago by Smith and Wodehouse (1938). This treatment recognized 38 species [including *V.oleifera* (Schott) A. C. Smith, now placed in its own genus *Bicuiba* W. J. de Wilde]. Smith and Wodehouse (1938) divided the genus into six groups: Mollissimae (I), Sebiferae (II), Calophyllae (III), Rugulosae (IV), Surinamenses (V), and Subsessilis (VI). The characters used to delimit these groups included trichome type, degree of perianth connation, spacing of secondary leaf veins, column shape, and anther apex shape. Since then, 27 binomials of *Virola* have been published (Ducke 1945; Smith 1953, 1956; Williams 1964; Little 1970; Gentry 1975; Rodrigues 1977, 1989; Sabatier 1997; Jaramillo et al. 2000; Santamaría-Aguilar et al. 2019; Vásquez Martínez and Soto Shareva 2020; Vásquez Martínez and Valenzuela Gamarra 2022). Additionally, floristic treatments and/or catalogs have been published for almost all the countries in South America where this genus occurs (Gradstein 2016 [Colombia]; Achá and Liesner 2014 [Bolivia]; Rodrigues 2015; Oliveira 2021 [Brazil]; Jaramillo et al. 2004 [Ecuador]; Mitchell 2002 [central French Guiana]; Aymard et al. 2007 [Guiana Shield]; Vásquez Martínez 2010; Vásquez et al. 2018 [Peru]; Rodrigues 2008; Rodrigues et al. 2001 [Venezuela]; Aymard et al. 2020 [Rio Negro basin: Brazil, Colombia and Venezuela]). Of these, the most comprehensive studies are Ecuador and Brazil, which treated 16 and 35 species, respectively (Rodrigues 1980; Jaramillo et al. 2004). Recent contributions include a vegetative identification key for 25 species of *Virola* (and four additional genera of Myristicaceae) from the Rio Negro basin (Aymard et al. 2020) and a key to the majority (i.e., 34 of 35) of species native to Brazil (Oliveira 2021). Despite its ecological and ethnobotanical importance, the phylogenetic analyses of the genus are limited, with a single well-sampled analysis supporting the resolution of two large subclades, informally called “Multinervae” and “Sebiferae” (Steeves 2011).

**Figure 4.**
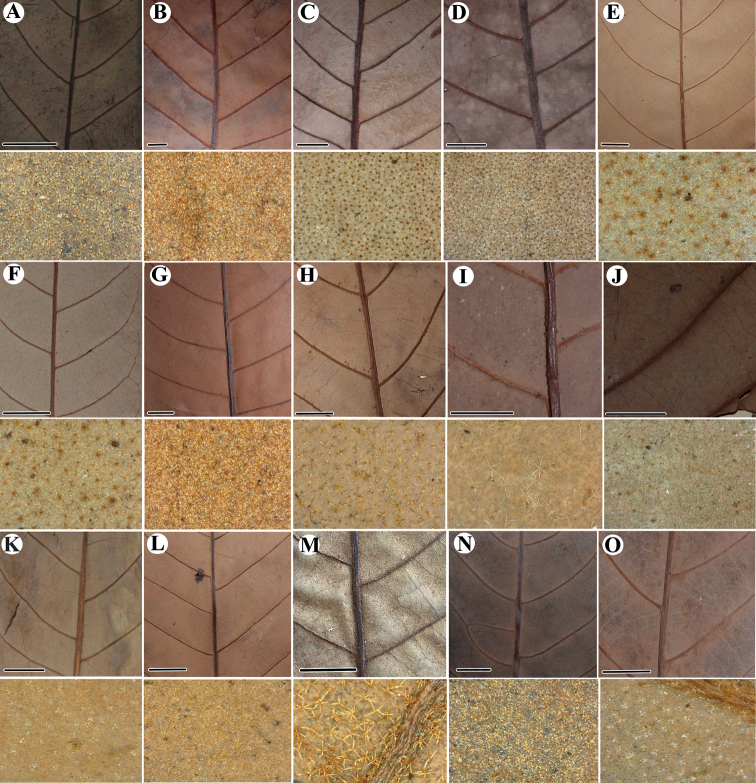
Comparation of pattern of the lateral veins and trichomes on abaxial leaf surface (below) of the newly described species of *Virola* and species that are morphologically similar **A***Virolaaguarunana* (*C. Díaz et al. 7700A*, MO) **B***V.alvaroperezii* (*G. Tipaz et al. 1789*, MO) **C***V.bombuscaroensis* (*J. Homeier 4507*, MO) **D***V.calimensis* (*M. Monsalve 1769*, MO) **E***V.calophylla* (*J. E. L. da S. Ribeiro et al. 1138*, MO) **F***V.calophylloidea* (*C. C. Berg et al. P18793*, MO) **G***V.cogolloi* (*Á. Cogollo et al. 4198*, MO) **H***V.excisa* (*C. E. Cerón & F. Hurtado 4147*, MO) **I***V.obovata* (*R. Vásquez & N. Jaramillo 3822*, MO; below from *A. [H.] Gentry & J. Revilla 20448*, MO) **J***V.macrocarpa* (*A. E. Lawrence 675*, **K** below *A. E. Lawrence 675*, MO) **K***V.parkeri* (*A. Monteagudo et al. 10761*, MO; below from *M. Huamán et al. 334*, MO) **L***V.peruviana* (*W. H. Lewis et al. 10074*, MO) **M***V.sebifera* (*R. Aguilar 2171*, MO) **N***V.tuckerae* (*Á. Cogollo et al. 4147*, MO) **O***V.yasuniana* (*H. Vargas & J. Cerda 678*, MO). Scale bars: 1 cm.

During a review of herbarium material from South America as part of the Myristicaceae treatment for “Flora Mesoamericana” (D. Santamaría-Aguilar, in rev.), we found several specimens that we consider fall outside of the morphological species concepts under which they were identified or treated. Most of this material was collected in the 1940s, 1980s, and 1900s, and was identified as *V.calophylla*, *V.macrocarpa* A. C. Sm., *V.obovata* Ducke, *V.peruviana* (A. DC.) Warb., and *V.sebifera*, or *V.multinervia* Ducke. We describe these as new species here. Morphologically, the new species appear similar to the species with which they were previously identified; however, detailed observation of complete specimens (i.e., those in which both flowers and mature fruits are present) reveals clear morphological differences in leaves, trichomes, inflorescences, staminate flowers, and, especially, fruits. It is likely that pistillate flowers are also distinct, although these are infrequent in herbarium specimens. With the exception of *V.cumala*, whose leaves have numerous and close lateral veins and dendritic trichomes, the other new species have leaves with well-separated lateral veins and stellate, sessile trichomes (usually with the central portion darkened) on the abaxial surface of leaf blades (Fig. 4). These characteristics are also shared with *V.allenii* D. Santam. & Aguilar, *V.amistadensis* D. Santam., and *V.otobifolia* D. Santam from Central America.

Below, we describe 10 new species of *Virola* from Colombia, Ecuador, Peru, and Brazil. This increases the total number of species in *Virola* to *ca.* 70, with 33, 20, 27 and 38 species, respectively, now known from each of these countries. Continued exploration and collection in South America likely will continue to reveal an abundance of new species and new distribution records of *Virola*.

## ﻿Materials and methods

Approximately 3000 physical herbarium specimens from Meso and South American *Virola* were examined for this study from the following herbaria: COL, CR (including ex INB), JAUM, LSCR, LSU, MO, NO, NY and USJ (acronyms follow Thiers 2021 [continuously updated]), although specimens from MO and NY represent the majority of the material studied. All type specimens, as well as general collections, hosted by virtual herbaria, were consulted, including those maintained by: the Field Museum (F; http://emuweb.fieldmuseum.org/botany/taxonomic.php), Instituto Nacional de Pesquisas da Amazônia (INPA; http://inct.florabrasil.net/en/), Jardín Botánico Juan María Céspedes (TULV; https://www.flickr.com/photos/98771984@N05/), JSTOR Global Plants (http://plants.jstor.org), Museum of Natural History, Paris (P; http://www.mnhn.fr), Oberösterreichischen Landesmuseums (LI; https://www.europeana.eu/portal/es), Reflora Virtual Herbarium (http://reflora.jbrj.gov.br/reflora/), speciesLink (https://specieslink.net/), Smithsonian Institution (US; https://collections.si.edu/search/), Universidad Nacional de Colombia (COL; http://www.biovirtual.unal.edu.co/en/), Universidad Nacional Autónoma de México (MEXU; https://datosabiertos.unam.mx/biodiversidad/) and the National Herbarium of The Netherlands (U; http://herbarium.naturalis.nl/).

Species descriptions are based primarily on herbarium specimens. If necessary and material permitted, flowers from herbarium specimens were rehydrated before measurement. A ruler was used to measure leaves and inflorescences; a digital Neiko caliper was used to measure fruits and seeds, as well as the thickness of the twigs, petioles and peduncles; and, finally, flowers, trichomes and thickness of the pericarp were measured with a micrometer calibration tool (1div = 1 mm) under a dissecting stereoscope (Bausch & Lomb).

Specimens cited are listed first by country. Within a country, specimens are listed alphabetically by major division and then alphabetically by department, province or state and, finally, in alphabetical order by the collector’s surname. When the coordinates and/or elevation were not included on the herbarium label, but were present in the TROPICOS database, the values from TROPICOS are included. Dot-distribution maps were compiled from studied specimens and generated with SimpleMappr (Shorthouse 2010).

Distribution, habitat, phenological data, common name and uses, flower and fruit colors, habit, bark, and exudate data were obtained from herbarium labels.

In the nomenclatural section for each new species, we cite both accession numbers and barcodes when present.

The preliminary conservation status of each new species was assessed using quantitative criteria recommended by the IUCN Red List (IUCN Standards and Petitions Subcommittee 2014). Georeferenced specimen data were used to determine the area of occupancy (AOO) and the extent of occurrence (EOO), which in turn were used to determine threat status. All analyses were performed in the R package conR (Dauby et al. 2017). When the recommendation differed between AOO and EOO assessments for a given species, we opted to conservatively recommend the more vulnerable status, following Knapp (2013).

## ﻿Taxonomy

### ﻿1. Virola aguarunana

Taxon classificationPlantaeMagnolialesMyristicaceae

D. Santam. sp. nov.

8D45C222-88FD-5FA9-8C30-4CEFBF415C32

urn:lsid:ipni.org:names:77298660-1

#### Type.

**Figure 5.**
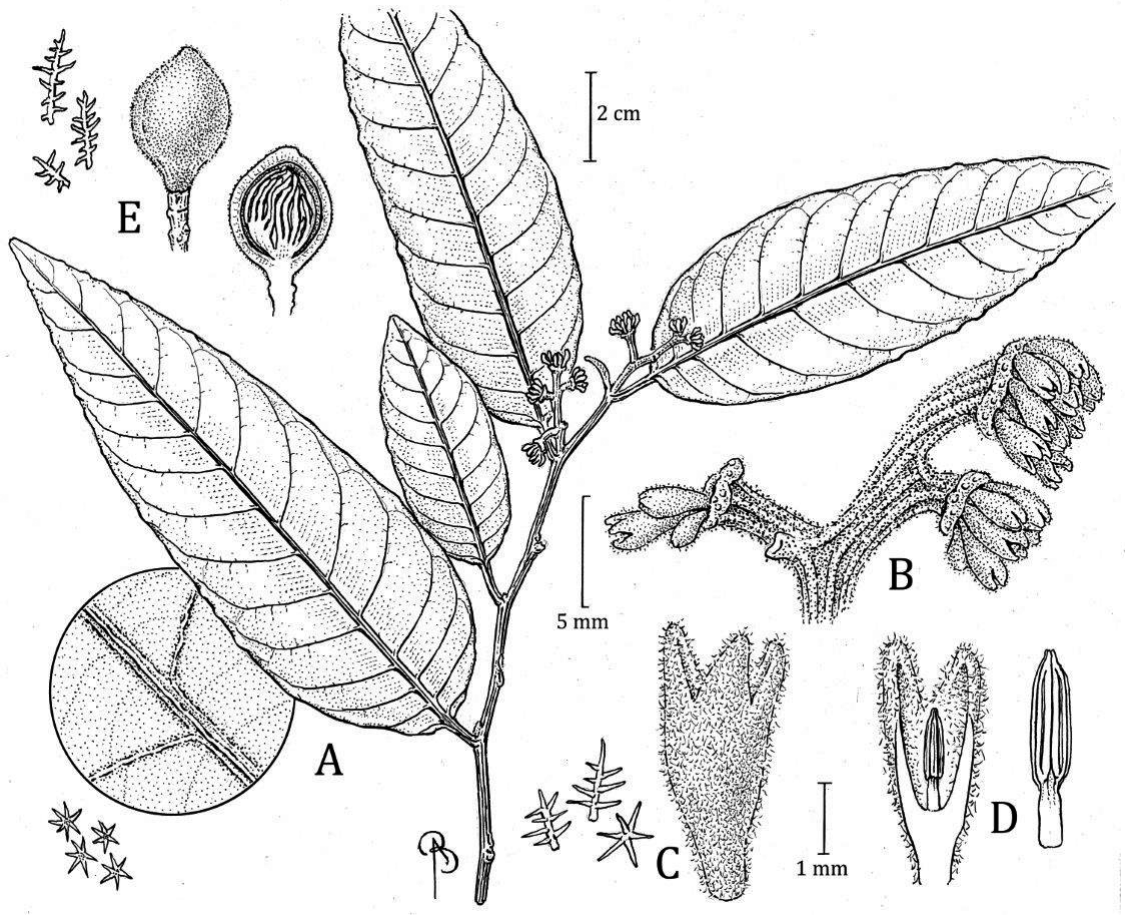
*Virolaaguarunana***A** branch with staminate inflorescence, including detail of abaxial leaf surface **B** partial staminate inflorescence **C** staminate perianth, with detail of trichomes (left) **D** medial sections of staminate perianth, showing the internal surface and androecium, with a closeup of the filament column and anthers (right) **E** fruit with detail of trichomes (left), with open fruit showing laciniate aril. Drawn by Bobbi Angell based on *C. Díaz et al. 7700A* (**A–E**; MO) and *C. Díaz et al. 7195* (**F**; MO).

**Peru. Amazonas**: Bagua, distrito Imaza, Comunidad Aguaruna Putuim (anexo Yamayakat), zona de colinas altas 24SW de Putuim, [05°00'47"S, 078°23'20"W], 700 m, 22 Sep 1994 (fr), *C. Díaz*, *A. Peña*, *& P. Ataim 7195* (holotype: MO! [accession 04808693, barcode MO-1405111]; isotypes: UPCB [n.v.]). Fig. 5

#### Diagnosis.

*Virolaaguarunana* was mainly confused with *V.calophylla*. Both species share stellate and sessile trichomes and well-separated lateral veins. Morphologically, it differs because the new species has sparsely pubescent abaxial leaf surface (vs. densely pubescent), long staminate perianth ([2–] 2.5–2.7 mm vs. 1–2.1 mm long), and fruits that are covered by a dense layer of trichomes (vs. covered with an inconspicuous layer of trichomes).

***Tree*** 16–20 m tall, diameter and inner bark not described. ***Exudate*** transparent, oxidizing to red, location of exudate on plant not stated. ***Twigs*** 0.18–0.27 cm thick, terete to slightly angled, tomentose to puberulent, trichomes dendritic, sessile, brown to ferruginous, sometimes slightly lenticellate. ***Leaves*** young terminal bud 0.8–1 × 0.16–0.18 cm; petiole 0.9–1.5 (–1.7) × 0.18–0.28 cm, slightly canaliculate, sometimes with very short alate, tomentose, the trichomes dendritic or sometimes stellate; leaf blades (10.7–) 14–22.7 × 4–8.5 cm, oblong-elliptic to ovate; adaxial surface of mature leaves that dry pale to dark brown or blackish, the surface smooth, sometimes shiny, glabrous; abaxial surface dries grayish pale to dark brown, sparsely pubescent, the trichomes stellate or sometimes stellate with dendriform trichomes along the main veins, the stellate trichomes 0.1–0.15 mm diameter, sessile, the central part of the trichome reddish, the branches pale brown-reddish or colorless; lateral veins 13–16 per side, 4–6 per 5 cm, spaced 0.8–1.1 (–1.5) cm, on adaxial side, the same color as the adaxial surface, flat, on abaxial surface raised, laterally compressed, tomentose, arcuate-ascending, slightly anastomosing near the margin and without forming a marked intramarginal vein; tertiary veins slightly visible on both leaf surfaces; midvein adaxially flat to slightly elevated, abaxially raised, rounded, laterally compressed or something triangular, tomentose; base acute to rounded, not revolute, flat; margin flat; apex acuminate. ***Staminate inflorescences*** 2.2–2.5 cm long, axes flattened, tomentose, the trichomes dendritic, ferruginous; peduncle 0.8–1.1 × 0.1–0.2 cm; main axes with 5 ramifications, the first pair opposite, the others alternate; bracts not seen. ***Staminate flowers*** (in bud) in dense terminal fascicles of 8–16+ flowers, borne on a receptacle (2–) 3.5–4.5 mm wide; perianth (2–) 2.5–2.7 mm long, obovate, fleshy, brown to brown greenish, connateconnate by 1.3–1.6 mm long, 1.3–1.6 mm long, external surface densely pubescent with ferruginous dendritic trichomes, internal surface moderately pubescent on the lobes; lobes 3, 0.9–1.2 (–1.4) × 0.3–0.8 mm and 0.1–0.2 mm thick, without resinous punctuations when rehydrated; stamens 3, the filament column 0.4–0.5 mm long and ca. 0.2–0.3 mm wide, glabrous, straight or rarely as a bottle shape, not constricted at the apex; anthers 0.6–0.9 mm long and 0.2–0.4 mm wide; apiculus 0.1–0.2 mm long or appearing absent (*R. Rojas et al. 486*), obtuse, connate. ***Pistillate inflorescences*** and ***pistillate flowers*** not seen. ***Infructescence*** 3.4–5 cm long, with 1–2 fruits, peduncle 1.5–2 cm × 0.35–0.46 cm. ***Fruits*** 2.9–3.5 × 1.8–2.2 cm, green or brown (probably due to pubescence) when fresh, pyriform to subglobose (with narrow base), stipitate, densely tomentose, the trichomes dendritic (ca. 0.2 mm long), ferruginous, and falling easily to the touch as dust, the surface slightly rugose, the line of dehiscence smooth to slightly carinate, the base obtuse, the apex acute to obtuse; pericarp 2–2.5 mm thick; pedicel 0.5–0.6 cm long. ***Seed*** ca. 2 × 1.6 the testa drying brownish reddish, grooved; aril color when fresh not described, blackish to brownish reddish when dry, the texture dry and thin, laciniate almost to the base, in narrow bands distally.

#### Distinctive characters..

**Figure 6.**
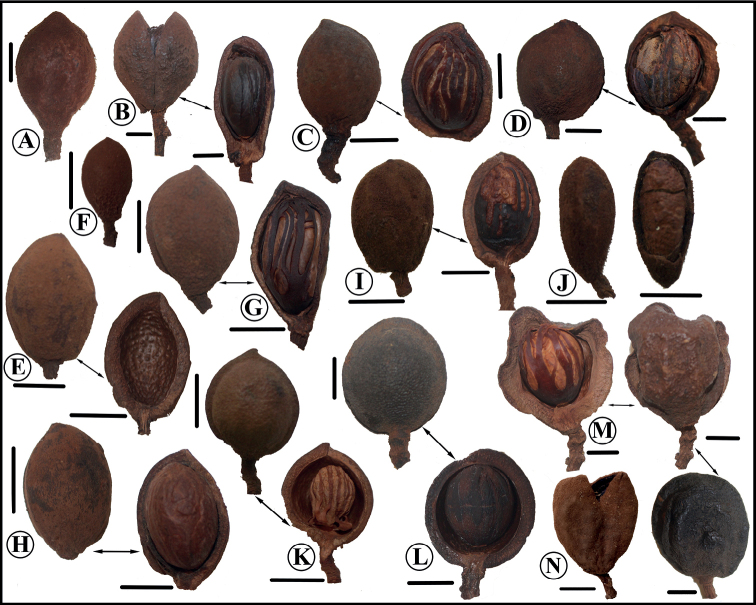
Comparison of fruits of newly described *Virola* species, with those species that are morphologically similar to them, noting the shape, indument, a pericarp thickness **A***V.aguarunana* (*C. Díaz et al. 7195*, MO) **B***V.alvaroperezii* (*D. Rubio et al. 2205*, MO) **C***V.bombuscaroensis* (*J. Homeier 4507*, MO) **D***V.calimensis* (*M. Monsalve 158*, MO) **E***V.calophylla* (*J. J. Pipoly 15614*, MO) **F***V.calophylloidea* (*S. R. Lowrie et al. 54*, MO) **G***V.cogolloi* (*D. Cárdenas & E. Alvarez 3239*, MO) **H***V.macrocarpa* (*A. E. Lawrance 675*, MO) **I***V.excisa* (*W. Palacios 3186*, MO) **J***V.obovata* (*R. Vásquez & N. Jaramillo 3822*, MO) **K***V.peruviana* (*D. N. Smith & V. García 13841*, MO). **L***V.parkeri* (*A. Monteagudo et al. 10761*, MO) **M***V.yasuniana* (*Á. Pérez & W. Santillán 4361*, MO; right and below from *V. Zak & S. Espinoza 5149*, MO) **N***V.tuckerae* (*Á. Cogollo et al. 3924*, COL). Scale bars: 1 cm.

*Virolaaguarunana* is recognized by its very short staminate inflorescences (2.2–2.5 cm long) with dense fascicles of flowers, relatively thin perianth that is externally densely pubescent and internally moderately pubescent, and a filament column that is shorter (0.4–0.5 mm long) than the anthers (0.6–0.9 mm long). It is further distinguished by its pyriform to subglobose fruits covered with ferruginous trichomes (Fig. 6A) that fall easy to the touch and leaf blades usually that are narrow with well-separated later veins and scattered pubescence of stellate and sessile trichomes, on the abaxial surface (Fig. 4A).

#### Etymology.

The specific epithet honors the Aguaruna people, who live in the area where this species was collected.

#### Distribution.

*Virolaaguarunana* is known only from the Amazonas Department of Peru (Fig. 18A). This tree has been collected along creek margins on rocky soils with abundant organic material at 700–800 m elevation. The region is also home to other notable species, including many magnoliids— *Compsoneuradiazii* Janovec (Myristicaceae), *Cremastospermabullatum* Pirie, *Cremastospermayamayakatense* Pirie (Annonaceae), *Ocoteaimazensis* van der Werff, *Ocotealeptophylla* van der Werff (Lauraceae)— as well *Dacryodesuruts-kunchae* Daly, M. C. Martínez & D. A. Neill (Burseraceae) and *Quipuanthusepipetricus* Michelang. & C. Ulloa (Melastomataceae), among many others.

#### Phenology.

Staminate buds and flowers of *Virolaaguarunana* have been recorded in January, October, and November; pistillate flowers were not seen in the studied material. Fruits have been collected in August and September.

#### Common name and uses.

No common names or uses are mentioned among the herbarium specimens observed.

#### Preliminary conservation status.

*Virolaaguarunana* is Endangered following IUCN criteria B1a and B2a. It is known from two localities, has an EOO of 98 km^2^, and an AOO of 12 km^2^. Further justifying this status, this very small distribution is combined with occurrence in areas known to be impacted by forest declines driven by shifting agriculture demands (Antonelli 2022). Of the few species we were able to verify, the most recent was collected in 1997.

#### Discussion.

Most of the specimens of *Virolaaguarunana* were previously determined as *V.calophylla* or *V.sebifera*, both widely distributed in South America. The new species shares some characteristics with *V.calophylla*, including a mixture of stellate and sessile trichomes and well-separated lateral veins (a feature shared with *V.sebifera*). However, *V.aguarunana* can be distinguished by its sparsely pubescent abaxial leaf surface (vs. densely pubescent; Fig. 4A, E), longer staminate perianth ([2–] 2.5–2.7 mm vs. 1–2.1 mm long), a filament column that is shorter than the anthers (vs. longer than anthers), and fruits that are covered by a dense layer of trichomes (vs. covered with an inconspicuous layer of trichomes; Fig. 6A, E). *Virolaaguarunana* shares the following traits with *V.sebifera*: a filament column that is shorter than the anthers, fruits covered by a dense layer of trichomes, and the same leaf traits as shared with *V.calophylla*. The new species differs from *V.sebifera*, by the sessile trichomes on the abaxial leaf surface (vs. usually pediculate; compare with Fig. 4M) and larger fruits (2.9–3.5 × 1.8–2.2 cm vs. 1–1.9 [–2.1] × 0.7–1.4 [–1.7] cm). Additionally, *V.aguarunana* can be differentiated in both species for its short and narrow staminate inflorescences with flowers borne in dense terminal fascicles. Further, *V.calophylla* and *V.sebifera* tend to have larger leaf blades.

#### Notes.

The only seed that could be measured is from one of the two fruits of the specimen *C. Díaz et al. 7195* (MO), which is not well preserved. The typical seed size for *V.aguarunana* is likely larger than presented here.

The specimen *D. Neill & Dendrology course students 15280* (MO; fr), from Cordillera del Cóndor, Zamora-Chinchipe, Ecuador may correspond to this species, though it is difficult to tell without flowers.

#### Specimens examined.

**Peru. Amazonas**: comunidad Aguaruna Putuim, anexo de Yamayakat, 700–750 m, 19 Jan 1996 (♂ fl), *C. Díaz et al. 7700A* (MO!); Comunidad Aguaruna de Wanás (Km 92 Carretera Bagua-Imacita), Cerros Chinim, 700–800 m, 29 Aug 1996 (fr), *C. Díaz et al. 8054* (F [image!], MO!); Comunidad Aguaruna de Kusú-Listra, Cerro Apág, margen derecha Quebrada Kusú, [05°02'24"S, 078°19'12"W], 550 m, 19 Nov 1996 (fl bud), *C. Díaz et al. 8086* (MO!); Tayu Mujaji, Comunidad de Wawas, 05°15'25"S, 078°21'41"W, 800 m 25 Oct 1997 (♂ fl bud), *R. Rojas et al. 486* (MO!).

### ﻿2. Virola alvaroperezii

Taxon classificationPlantaeMagnolialesMyristicaceae

D. Santam. sp. nov.

1D9442D9-ED21-599C-9E13-9ED331D901D4

urn:lsid:ipni.org:names:77298661-1

#### Type.

**Figure 7.**
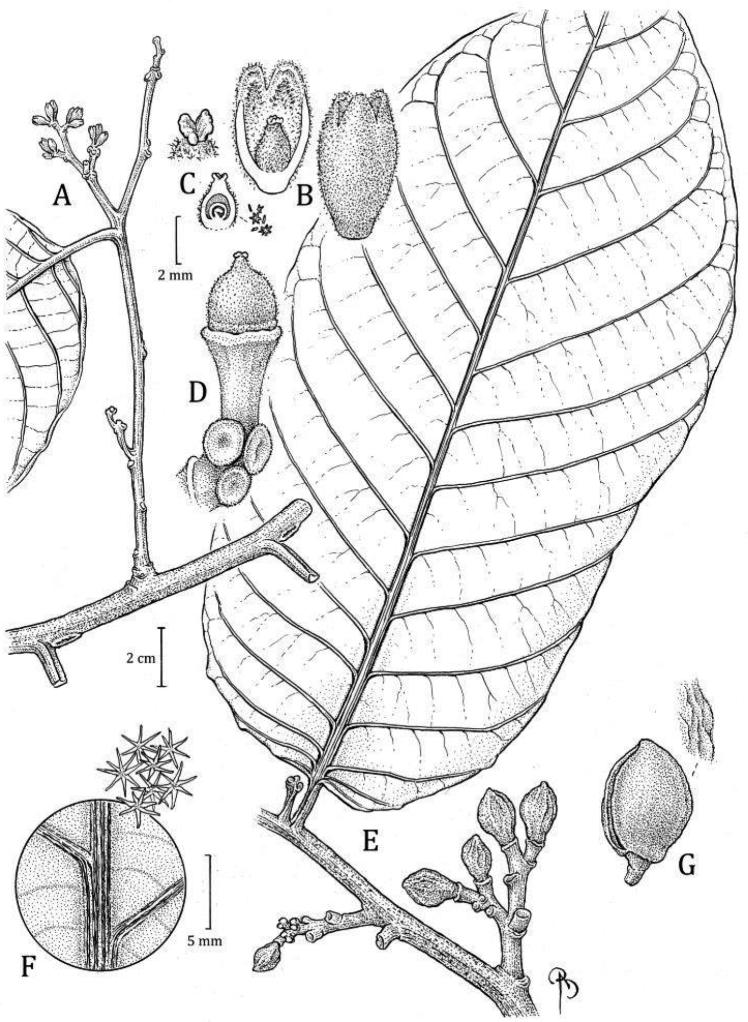
*Virolaalvaroperezii***A** branch with pistillate inflorescence **B** lateral view of pistillate perianth (right), as well as a medial section (left) showing the internal side and gynoecium **C** medial sections of gynoecium, with enlargement of trichomes (right) and stigma (top) **D** lateral view of gynoecium after perianth has fallen **E** leaf blade with infructescence **F** abaxial surface of leaf blade showing the midvein with enlargement of trichomes **G** fruit with detail of indument (right). Drawn by Bobbi Angell based on *G. Tipaz et al. 1789* (**A–B** MO-05005569), and *G. Tipaz et al. 1789* (**C–G** MO-05005570).

**Ecuador. Carchi**: Tulcan Cantón, Parroquia Tobar Donoso, Reserva Indígena Awá, Centro El Baboso, 00°53'N, 078°25'W, 1800 m, 17–27 Aug 1992 (♀ fl and fr), *G. Tipaz, M. Tirado, C. Aulestia, N. Gale & P. Ortiz 1789* (holotype: MO-2 sheets! [flowers: accession 05005569, barcode MO-1528199; fruits: accession 05005570, barcode MO-1528198]; isotypes: QCNE [n.v.]). Fig. 7

#### Diagnosis.

*Virolaalvaroperezii* is more similar to *V.macrocarpa* and *V.otobifolia* from Colombia, Panama, respectively. All these species have relatively large leaf blades and fruits, with lateral veins that are well separated and the abaxial leaf surface covered with stellate, sessile trichomes. Morphologically, it differs from *V.macrocarpa* and *V.otobifolia* by the abaxial leaf surface densely pubescent (vs. sparsely pubescent). Additionally, it differs from *V.macrocarpa* in having lateral veins that are more separated ([2–] 2.4–2.7 cm apart vs. 0.8–1.5 cm apart), and large fruits (4.3–4.5 × 3–3.6 cm vs. 2.7–3.3 × 2–2.3 cm). It differs from *V.otobifolia* by its wide fruit (3–3.6 cm vs. [1.9–] 2.3–2.9) and thin pericarp (2.8 mm vs. [2.7–] 3–4.7 mm).

***Tree*** 30–40 m × 60–70 cm diameter, inner bark and ***exudate*** not described. ***Twigs*** 0.37–0.4 cm thick, terete to slightly compressed, inconspicuously pubescent, trichomes dendritic, sessile, ferruginous, without lenticels. ***Leaves*** young terminal bud not seen; petiole 1.5–2 × 0.34–0.5 cm, terete to slightly canaliculate, sometimes slightly winged, tomentose to tomentulose, the trichomes dendritic; leaf blades 26–28.5 × 12.1–14 cm, widely elliptic; adaxial surface of mature leaves drying brown to brown-olivaceous, the surface smooth, sometimes shiny, glabrous; abaxial surface drying dark brown-reddish to brown-whitish, densely pubescent, the trichomes stellate, ca. 0.1 mm diameter, sessile, the central part of the trichome dark reddish or colorless, the branches brown-reddish or colorless; lateral veins ca. 15, ca. 3 veins per 5 cm, spaced (2–) 2.4–2.7 cm, adaxially the same color as the leaf surface or slightly darker, flat to slightly raised, abaxially brown to blackish, raised, glabrescent above, densely pubescent to the sides, arcuate-ascending distally, slightly anastomosing near the margin and without forming a marked intramarginal vein; tertiary veins lightly visible on both sides, but especially below; midvein adaxially flat to slightly elevated, abaxially raised, rounded, glabrescent to tomentose; base obtuse, not revolute, flat; margin flat; apex absent. ***Staminate inflorescence*** and ***flowers*** unknown. ***Pistillate inflorescence*** ca. 4.2 cm long, axes flattened, tomentose, with trichomes dendritic, ferruginous; peduncle ca. 1.2–1.7 × 0.3–0.35 cm; main axes with 5 ramifications; bracts not seen. ***Pistillate flowers*** in terminal fascicles of 2–3 flowers, on a receptacle ca. 6 mm wide; perianth ca. 6 mm long, lanceolate, fleshy, brown when fresh, connate by ca. 3–3.2 mm long, external surface densely pubescent with ferruginous and dendritic trichomes, internal surface pubescent (especially on the lobes); lobes 3, ca. 2.8 × 1.2–1.5 mm and ca. 0.5–0.6 mm thick, without resinous punctuations when rehydrated; gynoecium ca. 2.5–3.1 × 1.5–1.6 mm, conical to subglobose, densely pubescent, the trichomes ferruginous; stigma 2-lobed, ca. 0.5 × 0.3 mm, erect, flat seen from above, drying blackish, slightly wavy at the margins. ***Infructescence*** ca. 4.5 cm long, with 1 or 4 fruits in immature infructescence, peduncle ca. 1.5 × 0.8 cm. ***Fruits*** 4.3–4.5 × 3–3.6 cm, brown when fresh (probably by the indument), ellipsoid, shortly stipitate, densely tomentose, the trichomes dendritic, sessile, ferruginous, not falling to the touch like dust, the surface rugose, the line of dehiscence smooth to faintly carinate, the base obtuse to truncate, the apex obtuse; pericarp 2.8 mm thick; pedicel 0.6–1 cm long. ***Seed*** ca. 2.7 × 1.6 cm, the testa dark brown to blackish when dry, sulcate; aril described once as red when fresh, dark brown when dry, the texture dry and thin, laciniate almost to the base, in narrow bands distally.

#### Distinctive characters.

*Virolaalvaroperezii* is a distinctive species characterized by generally large, wide leaf blades with well-separated lateral veins that are abaxially covered with dense pubescence of stellate, sessile trichomes (Fig. 4B) and large fruits (4.3–4.5 × 3–3.6 cm) with thick pericarp (2.8 mm) (Fig. 6B). It is also distinctive for being a large tree (30–40 m tall).

#### Etymology.

It is a great pleasure to dedicate this new species to the Ecuadoran botanist Álvaro Javier Pérez Castañeda. He is an excellent botanist, collector and expert of the flora of Ecuador, especially the flora of Yasuní. Among other contributions, he has described at least 26 from different angiosperm families (e.g., Pérez et al. 2013; Torke and Pérez 2013; Kawasaki and Pérez 2015, 2016) and is coauthor of *Árboles emblemáticos de Yasuní, Ecuador* (Pérez et al. 2014) and the treatment of Myrtaceae for the *Flora of Ecuador* (Kawasaki et al. 2019). Pérez Castañeda collected specimens of some of the *Virola* species described here.

#### Distribution.

*Virolaalvaroperezii* is know from Carchi and Esmeraldas provinces in Ecuador (Fig. 18A). It grows in primary vegetation in premontane rain and very wet forest from (500–) 1600–1800 m.

#### Phenology.

Only a single studied specimen of *Virolaalvaroperezii* has pistillate flowers; it was collected in August. Staminate flowers were not observed in the studied material. Fruits were collected in August and September.

#### Common name and uses.

Guangare macho (Ecuador; *C. Aulestia & M. Aulestia 1017*).

#### Preliminary conservation status.

*Virolaalvaroperezii* is Endangered following IUCN criteria B1a and B2a. It is known from three localities, has an EOO of 966 km^2^, and an AOO of 12 km^2^. Further justifying this status, this new species occurs in a region of very high rates of deforestation due to agricultural pressures (Kleemann et al. 2022). The only specimens of *V.alvaroperezii* that we were able to verify were collected on the Awá Reserve, and this species may be locally protected by sustainable forestry practices by the Awá indigenous community (Oviedo 2006).

#### Discussion.

Herbarium specimens of *Virolaalvaroperezii* have been confused with another montane species, *V.macrocarpa* from Colombia (1100 m elevation). It could be confused with another Colombian species, *V.cogolloi* (840–1500 m elevation), which is formally described here, as well with *V.otobifolia* D. Santam., recently described from Panama (50–850 m elevation) (Santamaría-Aguilar et al. 2019). All these species have relatively large leaf blades and fruits, with lateral veins that are well separated and the abaxial leaf surface covered with stellate, sessile trichomes. Differences among the three species are summarized in Table 2.

**Table 2.** Comparison of *Virolaalvaroperezii*, with *V.cogolloi*, *V.macrocarpa*, and *V.otobifolia*. ^†^From Smith and Wodehouse (1938).

Morphological character	*V.alvaroperezii*	*V.cogolloi*	*V.macrocarpa*	*V.otobifolia*
Leaf blade size, and pubescence abaxially	26–28.5 × 12.1–14 cm; densely pubescent (Fig. 4B)	25–29.7 × (9.2–) 10.8–15 cm; densely pubescent (Fig. 4G)	20–40 × 7–11 cm^†^; sparsely pubescent (Fig. 4J)	(14–) 18.2–42.5 × (4.1–) 7.3–14.2 cm; sparsely pubescent
Spaced lateral veins	(2–) 2.4–2.7 cm apart	1.9–2.5 cm apart	0.8–1.5 cm apart	1.7–3 cm apart
Fruit size, and pubescence	4.3–4.5 × 3–3.6 cm; with persistent trichomes (Fig. 6B)	2.7–3.2 × 1.7–2.2 cm; the trichomes do not fall easily like dust (Fig. 6G)	2.7–3.3 × 2–2.3 cm^†^; with caducous trichomes (Fig. 6H)	(2.7–) 3.5–4.5 × (1.9–) 2.3–2.9 cm; densely tomentose, with persistent trichomes (Fig. 3D)
Pericarp thickness	2.8 mm	1.2–1.6 mm	1.8–3 mm	(2.7–) 3–4.7 mm
Seed	2.7 × 1.6 cm	2.2–2.4 × 1.3–1.4 cm	2.2–2.5 × 1.5–1.7 cm^†^	2.5–2.8 × 1.5–1.7 cm

#### Notes.

The holotype deposited at Missouri Botanical Garden (MO) represents a single collection mounted on two sheets that are clearly labeled (i.e., “Sheet 1 of 2” and “Sheet 2 of 2”) as being parts of the same specimen (ICN Art. 8.3; Turland et al. 2018); one of the sheets carries pistillate flowers (MO-05005569) and the other, fruits (MO-05005570).

Leaf size and the number of lateral veins may be higher than presented here, as most of the material examined had broken leaves.

The collection *C. Aulestia & M. Aulestia 1017* (MO) from Esmeraldas Province, Ecuador bears a fruit (possibly immature) that is smaller (ca. 3.7 × 2.6 cm) with thinner pericarp (ca. 2 mm) than other specimens of *V.alvaroperezii*. However, it otherwise matches very well including in its leaf morphology, tall tree habit, and occurrence Río Mira basin; for this reason, it is included in the concept we have adopted, although the fruit measurement is not included in the description.

The fruit specimen *H. Vargas et al. 4603* (MO, QCNE [image!]), also from Esmeraldas Province, Ecuador, could not be confirmed to the species. It is similar to *V.alvaroperezii* in its leaf morphology (e.g. size, pubescence and trichomes, lateral vein spacing); however, its fruits are rounded and smaller (ca. 3.2–3.3 × 2.6–2.7 mm) with a conspicuous carina.

The two collections of *V.macrocarpa* from Carchi Province that are cited in *Flora of Ecuador* (Jaramillo et al. 2004) correspond to this new species.

#### Specimens examined.

**Ecuador. Carchi**: Tulcán cantón, Reserva Indígena Awá, Comunidad El Baboso, 12 km al norte de Lita, 00°53'N, 078°20'W, 1600 m, 20 Sep 1991 (fr), *D. Rubio et al. 2205* (MO!, QCNE [n.v.]). **Esmeraldas**: Reserva Etnica Awá, Parroquia Ricaurte, Centro Pambilar, 01°08'N, 078°36'W, 500 m, 21 Jan 1993 (fr), *C. Aulestia & M. Aulestia 1017* (MO!, QCNE [n.v.]).

### ﻿3. Virola bombuscaroensis

Taxon classificationPlantaeMagnolialesMyristicaceae

D. Santam. sp. nov.

AE1F6C2C-B36D-51FD-A076-7101C3F378A2

urn:lsid:ipni.org:names:77298662-1

#### Type.

**Figure 8.**
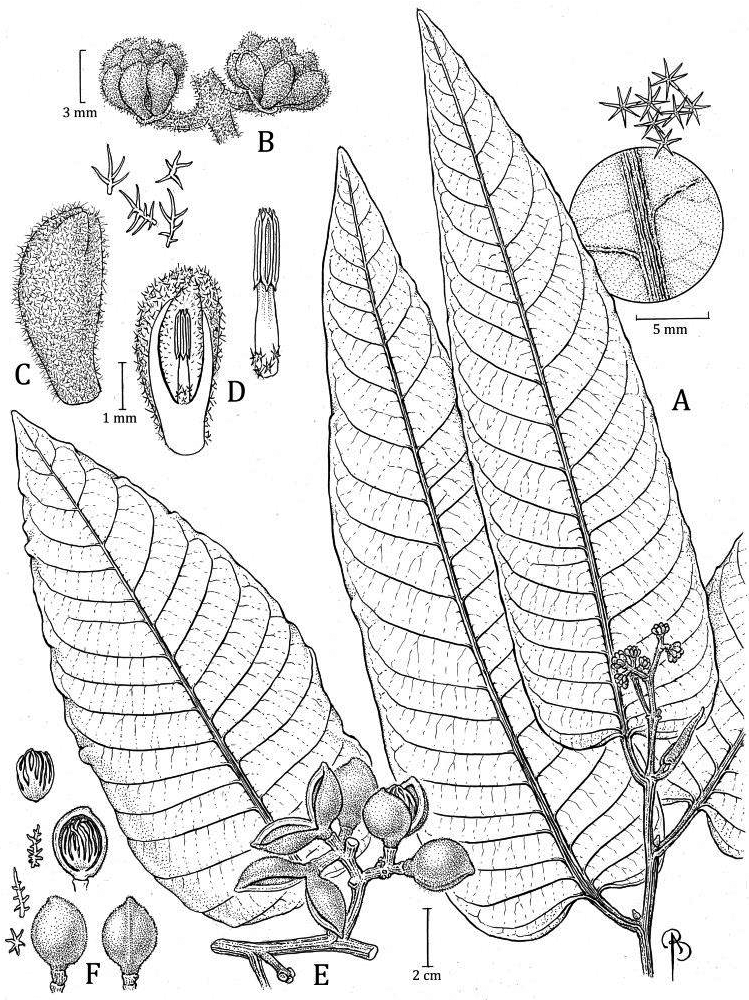
*Virolabombuscaroensis***A** branch with staminate inflorescence and detail of trichomes on abaxial surface **B** partial staminate inflorescence in bud **C** external surface of staminate perianth (in bud), with detail of trichomes (top right) **D** medial section of staminate perianth showing the internal surface and androecium, with a close-up of the filament column with trichomes at bas and anthers (right) **E** branch with infructescence **F** different views of the fruits, detail of trichomes (left), and open fruit showing the seed with laciniate aril (above). Drawn by Bobbi Angell based on *J. Homeier 1090* (**A–E** MO), and *J. Homeier 4507* (**F–G** MO).

**Ecuador. Zamora-Chinchipe**: PN [Parque Nacional] Podocarpus, Bombuscaro entrance, 04°07'S, 078°58'W, 1350 m, 25 Jan 2011 (fr), *J. Homeier 4507* (holotype: MO! [accession 6863380, barcode MO-3053475]; isotypes: n.v.). Fig. 8

#### Diagnosis.

*Virolabombuscaroensis* differs morphologically from all other species by the combinations of abaxial leaf surface covered by stellate, sessile trichomes, perianth of staminate flowers densely pubescent on both faces, and the column of filaments that has scattered trichomes at the base.

***Tree*** 6–15 m tall, diameter, inner bark and ***exudate*** not described. ***Twigs*** 0.43–0.62 cm thick, terete to laterally flattened, tomentose to tomentulose, trichomes dendritic, sessile, ferruginous to whitish, the bark sometimes with small lenticels. ***Leaves*** young terminal bud 2.5–2.9 × 0.4–0.48 cm, with conspicuous veins; petiole 1.5–2.2 × 0.33–0.5 cm, canaliculate, sometimes very short alate, tomentose, the trichomes dendriform; leaf blades 23.3–30 × 7.1–10.3 cm, ovate; adaxial surface of mature leaves dries brown to olivaceous, glabrous or with very few and scattered trichomes (densely pubescent on new leaves), the surface smooth, sometimes shiny; abaxial surface when drying brown or whitish-grayish, densely pubescent, but inconspicuous to the naked eye, trichomes stellate or sometimes dendriform trichomes along the main veins, the stellate trichomes 0.1–0.2 mm diameter, sessile, the central part of the trichome (usually) reddish and contrasting in color with the hyaline to reddish branches, or the branches the same color as the central part, the dendriform trichomes ca. 0.4 mm long, ferruginous, persistent; lateral veins (13–) 18–23 per side, (3–) 4–5 veins per 5 cm, spaced (0.9–) 1.4–1.8 cm apart, on adaxial surface flat or elevated, the same color as the adaxial leaf surface or a little darker, on abaxial surface raised or flat, rounded or laterally compressed, tomentose to glabrescent, arcuate-ascending, slightly anastomosing near the margin and without forming a very marked intramarginal vein; tertiary veins slightly visible on both sides, more prominent on adaxial side; midvein adaxially slightly elevated, abaxially raised, usually rounded, tomentose; base cordate, not revolute, flat; margin flat to slightly revolute; apex acuminate. *Staminate inflorescences* ca. 5.4 cm long, axes flattened, tomentose, the trichomes dendritic, ca. 0.3–0.4 mm long, ferruginous; peduncle ca. 2 × 0.27 cm; main axes with 5 ramifications, the first pair opposite, the others alternate; bracts not seen. ***Staminate flowers*** (in bud) in dense terminal fascicles of 13–15+ flowers, on a receptacle 2–2.7 mm wide; perianth 2.8–3.4 mm long, obovoid, fleshy, brown when fresh, connation not seen (young flowers), external surface densely pubescent with ferruginous and dendritic trichomes, internal surface densely pubescent; lobes 3, not measured (young flowers), ca. 0.2 mm thick, without resinous punctuations when rehydrated; stamens 3, the filament column ca. 1.4 mm long, ca. 0.4 mm wide, slightly pubescent at the base, straight to slightly thickened at the base, not constricted at the apex; anthers ca. 0.9 mm long, ca. 0.4 mm wide; apiculus ca. 0.1 mm long, apiculate, separate. ***Pistillate inflorescences*** and ***pistillate flowers*** not seen. ***Infructescence*** 3–3.5 cm long, with 2–6 fruits, peduncle 1.8–3 × 0.43–0.5 cm. ***Fruits*** 2.4 × 1.8–1.9 cm, brown when fresh (probably by the pubescence), globose, stipitate, densely tomentose, the trichomes dendritic, ca. 0.1–0.3 mm long, ferruginous, and falling easily to the touch as dust, the surface slightly rugose, the line of dehiscence slightly carinate, but not very conspicuous, the base obtuse, the apex acute to obtuse; pericarp 1.6–2.2 mm thick; pedicel 0.5–0.7 cm long. ***Seed*** 1.8–1.9 × 1.2–1.4 cm, the testa whitish grayish to brownish when dry, grooved; aril color when fresh not described, blackish to brownish red when dry, the texture dry and thin, laciniate almost to the base, in narrow bands distally.

#### Distinctive characters.

*Virolabombuscaroensis* can be recognized by many pubescence characters. Dendritic trichomes cover its branches, petioles, inflorescence axes, and sometimes along the main veins on the abaxial sides of leaves; the abaxial leaf surface always is marked by stellate, sessile trichomes (Fig. 4C); its fruits are covered by ferruginous trichomes that fall very easily to the touch (Fig. 6C); the perianth of staminate flowers is densely pubescent on both sides; and the column of filaments has scattered trichomes at the base. Finally, the filament column is straight, not constricted at the apex, and is longer (ca. 1.4 mm) than the anthers (ca. 0.9 mm).

#### Etymology.

The specific epithet makes reference to the Bombuscaro River in the Podocarpus National Park of southern Ecuador, where most of the collections of this new species were made.

#### Distribution.

*Virolabombuscaroensis* is known only from Ecuador (Zamora-Chinchipe Province) (Fig. 18A). It is found in premontane forest at elevations between 1200 to 1350 m, with a single collection from 1930 m in elevation.

#### Phenology.

Staminate flowers of *Virolabombuscaroensis* have been recorded in April and fruits in January. Pistillate flowers were not seen in the studied material.

#### Common name and uses.

None recorded.

#### Preliminary conservation status.

*Virolabombuscaroensis* is Critically Endangered following IUCN criterion B2a. While its very small range from only a single locality justifies this status, *V.bombuscaroensis* benefits from its occurrence in Podocarpus National Park and surrounding areas, which are highly protected within Ecuador (Kleemann et al. 2022).

#### Discussion.

*Virolabombuscaroensis* is difficult to confuse with other species of *Virola* described to date. However, it shares the combination of a cordate leaf base, well-separated lateral veins, sessile trichomes on abaxial leaf surface, and fruits with a conspicuous layer of trichomes with *V.excisa* (described here). Other species with similar leaf morphology, but with fruits with less conspicuous trichomes include: *V.calophylla* (Fig. 6E), *V.peruviana* (Fig. 6K), and *V.schultesii* A. C. Sm.; they can be distinguished by the characters in Table 3.

**Table 3.** Comparison of *Virolabombuscaroensis* with the morphologically most similar species. ^†^From Smith and Wodehouse (1938); ^§^from Smith (1956) (except trichomes on filament column and fruit carina); ^¶^from Vásquez Martínez and Soto Shareva (2020).

Morphological character	*V.bombuscaroensis*	*V.calophylla*	*V.excisa*	*V.peruviana* ^†^	*V.pseudosebifera ^¶^*	*V.schultesii* ^§^
Leaf base	Cordate	Usually deeply cordate to truncate (obtuse)	Truncate to subcordate, rarely deeply cordate	Deeply to shallowly cordate	Cordate to rounded	Deeply cordate
# of lateral veins	(13–) 18–23	11–28	(13–) 18–24	17–30	(10–) 12–18	14–19
Perianth staminate long and trichomes inside	2.8–3.4 mm; densely pubescent	1–2.1 mm; glabrescent	1.5–2.8 mm; almost glabrous	2.3–3.2 mm; puberulent	3–3.5 mm; tomentose (in pistillate flowers)	2–2.3 mm long; pubescent
Long filament column, and trichomes	ca. 1.4 mm; slightly pubescent at the base	0.2–0.6 mm; glabrous	(0.4–) 0.5–0.8 mm; glabrous	0.4–0.6 mm; glabrous	0.4–0.7 mm; trichomes not described	0.3–0.4 mm; glabrous
Fruit size, and trichomes	2.4 × 1.8–1.9 cm; densely tomentose, falling easily to the touch as dust (Fig. 6C)	2.5–3 × 1.2–2.5 cm; tomentelous to glabrescent, not falling easily to the touch as dust (Fig. 6E)	2–2.6 × 1.4–1.7 cm; densely tomentose, falling easily to the touch as dust (Fig. 6I)	2–2.8 × 1.5–2.2 cm; glabrescent (Fig. 6K)	2.8–3.6 ×1.8–2.4 cm, densely tomentose	0.8–1.1 × 0.7–1 cm; minutely pubescent, not falling easily to the touch as dust
Carina on the line of dehiscence	Slightly carinate	Carinate	Slightly carinate	Conspicuously carinate	Slightly carinate	Not carinate
Pericarp thick	1.6–2.2 mm	0.5–5 mm	1.5–2.3 mm	0.5–1.5 mm	2–3 mm	0.5 mm

**Figure 9.**
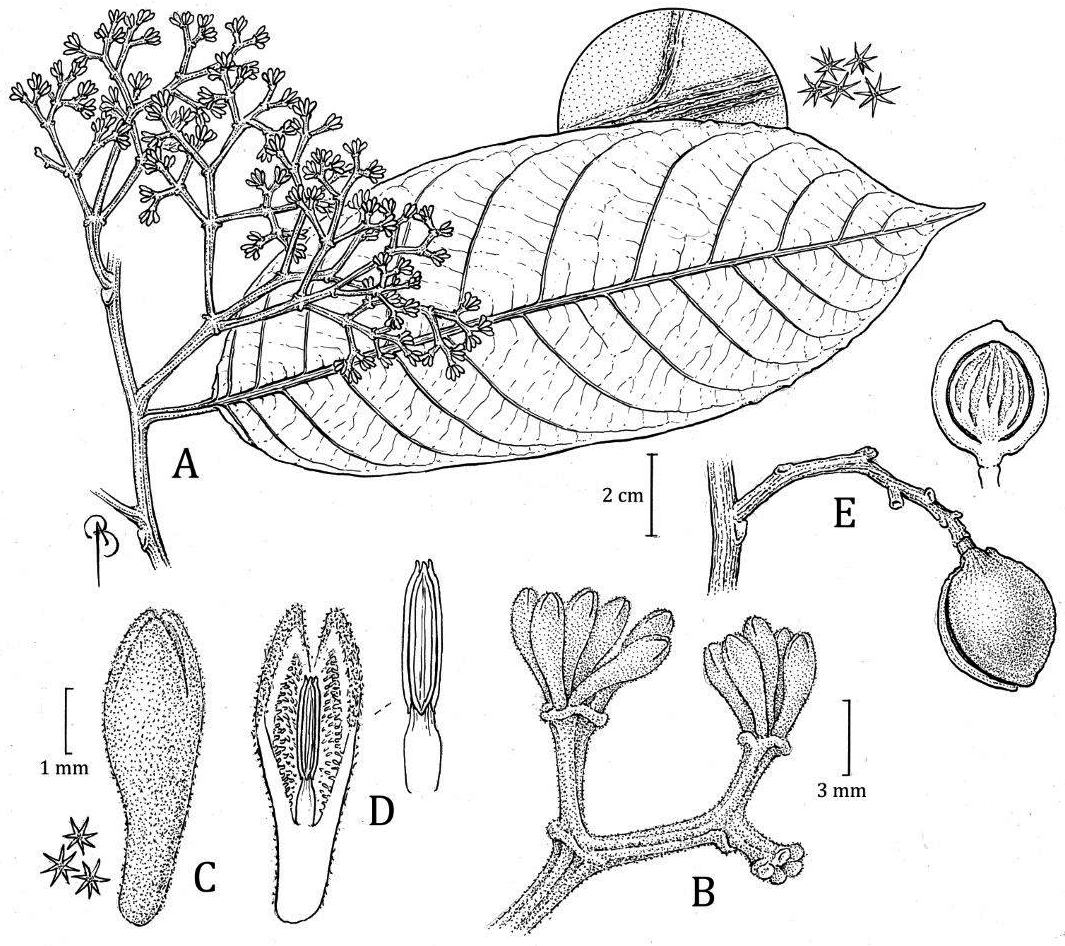
*Virolacalimensis***A** branch with staminate inflorescence and detail of trichomes on abaxial surface **B** closeup of partial staminate inflorescence **C** staminate perianth, with detail of trichomes (left) **D** medial sections of staminate flower showing the internal surface and androecium, with a closeup of filament column and anthers (right) **E** partial infructescence and open fruit (above), showing the seed with laciniate aril. Drawn by Bobbi Angell based on *C. Jativa & C. Epling 1143* (**A–D** MO), and *M. Monsalve 158* (**E** MO).

**Figure 10.**
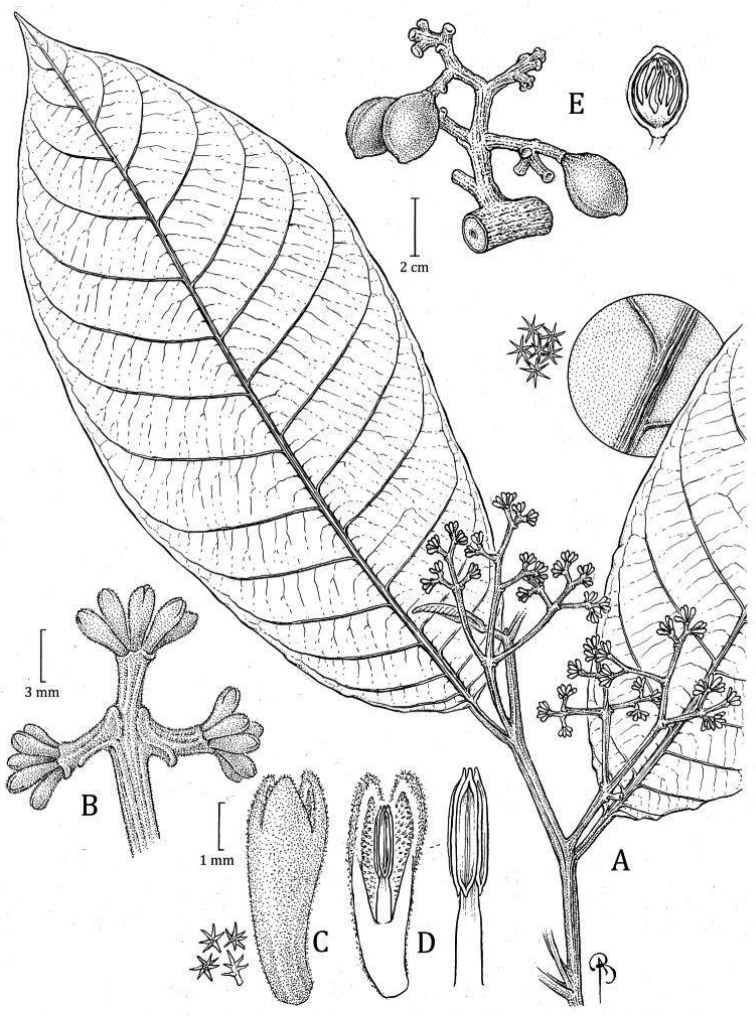
*Virolacogolloi***A** branch with staminate inflorescence, with detail of trichomes on abaxial surface **B** closeup of partial staminate inflorescence **C** staminate perianth, with detail of trichomes (left) **D** medial sections of staminate flower showing the internal surface and androecium, with a closeup of filament column and anthers (right) **E** partial infructescence and open fruit showing the seed with laciniate aril. Drawn by Bobbi Angell based on *Á. Cogollo 4198* (**A–D** MO), and *D. Cárdenas & E. Alvarez 3239* (**E** MO).

Other species with similar leaf morphology (i.e., a usually deeply cordate base and well-separated lateral veins) are: *V.divergens* Ducke, *V.mollissima* (Poepp. ex A. DC.) Warb., and *V.sebifera*. However, these species differ in having pediculate trichomes on the abaxial leaf surface (vs. sessile in *V.bombuscaroensis*), internally puberulent staminate perianth (vs. densely pubescent), a glabrous filament column (vs. with scattered trichomes at the base; Fig. 10D) that is shorter (vs. longer) than the anthers. In fruiting material, *V.divergens*, and *V.mollissima* are covered with a thick layer of long hairs (at least 1.4 mm long) that differ from *V.bombuscaroensis*. While the layer of trichomes on the fruit are somewhat similar to the new species in *V.sebifera*, the fruits are notably smaller (1–1.9 [–2.1] × 0.7–1.4 [–1.7] vs. 2.4 × 1.8–1.9 cm), and *V.sebifera* usually occurs at lower elevations.

Additionally, compared to the species with with it can be confused, *V.bombuscaroensis* tends to have longer staminate inflorescences and more obviously pubescent twigs, petioles, and abaxial leaf surface, very often with dendritic trichomes.

#### Notes.

The specimen *J. Homeier 1090* was cited as *Virola* sp. 1, in Spermatophyta/*Checklist Reserva Biológica San Francisco (Prov. Zamora-Chinchipe, S-Ecuador)* (Homeier and Werner 2007), while *W. Palacios & M. Tirado 13379* was discussed under *V.peruviana* in *Flora of Ecuador* (Jaramillo et al. 2004).

Two collections from Cajamarca Department, Peru collected at 1500–1700 m elevation, *J. Campos & S. Nuñez 4589* (MO!, NY-2 sheets!, UPCB [n.v.]) bearing staminate flowers and *J. Campos & S. Nuñez 4188* (MO, NY, UPCB [n.v.]) bearing an infructescence with very immature fruits, have similar leaf morphology to *V.bombuscaroensis* (i.e., cordate base and abaxial surface with stellate, sessile trichomes). However, these specimens differ in their longer staminate inflorescences (ca. 8 cm vs. ca. 5.4 cm long) and the shorter (ca. 0.4 mm vs. ca. 1.4 mm long), glabrous (vs. pubescent) filament column. Additional, more complete material is needed to assess whether these are conspecific. These specimens have been identified as *V.calophylla* and duplicates may have been distributed under this name.

#### Specimens examined.

**Ecuador. Zamora-Chinchipe**: Area of the Estación Científica San Francisco, road Loja-Zamora, ca 35 km from Loja, 03°58'S, 079°04'W, 1930 m, 6 Apr 2002 (fl ♂), *J. Homeier 1090* (MO!); Zamora cantón, Parque Nacional Podocarpus, guardería Río Bombuscaro, 04°05'S, 078°57'W, 1200 m, [s.d.] Jan 1995 (fr), *W. Palacios & M. Tirado 13379* (MO!, UPCB [n.v.]).

### ﻿4. Virola calimensis

Taxon classificationPlantaeMagnolialesMyristicaceae

D. Santam. sp. nov.

ED3F4679-1AD6-5D56-9DB0-52BB466B8F71

urn:lsid:ipni.org:names:77298663-1

#### Type.

**Colombia. Valle del Cauca**: Bajo Calima, concesión Pulpapel/ Buenaventura, 03°55'N, 077°00'W, 100 m, 03 Aug 1984 (fr), *M. Monsalve 158* (holotype: MO! [accession 3624779, barcode MO-2657915]; isotype: INPA [accession 147738; image!], JAUM [accession 007410]). Fig. 9

#### Diagnosis.

*Virolacalimensis* was previously confused with *V.calophylla* and *V.macrocarpa*. Perhaps because of stellate, sessile trichomes and lateral veins that are well separated. Morphologically it differs from *V.calophylla* in having flowers with longer staminate perianth (2.5–3.5 mm vs. 1–2.1 mm), the internal surface densely pubescent (vs. glabrescent), long anthers (0.6–1 [–1.2] mm vs. 0.4–0.5 mm), and large fruits (2.8–3.1 cm long vs. 1.6–1.5 cm). Differs from *V.macrocarpa* in leaf blades abaxially densely pubescent (vs. sparsely pubescent), fruits globose (vs. ellipsoid), shorter (2.8–3.1 cm long vs. [2.7–] 3.5–4.5 cm).

***Tree*** 5–20 or 30 m tall × 25–28 cm in diameter, inner bark described once as brown, cracked, rough. ***Exudate*** reddish, location of exudate on plant not stated, or described as being present in the inflorescence, but with no information about color. ***Twigs*** 0.3–0.33 cm thick, terete or slightly compressed, inconspicuously pubescent, trichomes dendritic, sessile, brown reddish, without lenticels. ***Leaves*** young terminal bud not seen; petiole 1.5–2.4 × 0.22–0.4 cm, slightly canaliculate, sometimes slightly winged, glabrescent, or inconspicuously tomentose to tomentulose, the trichomes dendritic; leaf blades 19–25 × 7.3–11 cm, widely oblong to elliptical; adaxial surface of mature leaves drying brown to olive, the surface smooth, sometimes shiny, glabrous; abaxial surface drying brown reddish to grayish brown, densely pubescent, the trichomes stellate, ca. 0.1 mm diameter, sessile, the central part of the trichome dark reddish, the branches pale reddish; lateral veins ca. 12–13 (*W. Devia 3086*), 3–4 veins per 5 cm, spaced 1.3–2.3 cm apart, flat to slightly raised on adaxial surface, raised on abaxial surface and slightly darker than on adaxial surface, puberulent, densely tomentose to glabrescent above, densely pubescent to the sides, arcuate-ascending distally, slightly anastomosing near the margin and without forming a marked intramarginal vein; tertiary veins slightly visible on both sides; midvein adaxially slightly elevated, abaxially raised, rounded to rectangular, glabrescent to tomentose; base obtuse, not revolute, flat; margin flat; apex acuminate. ***Staminate inflorescence*** 6–8 cm long, axes flattened, tomentose, with trichomes dendritic, brown-reddish; peduncle 1.3–2.8 × 0.28–0.4 cm; main axes with 5–11 ramifications, the first pair opposite to subopposite, the others alternate; bracts not seen. ***Staminate flowers*** (in bud) in lax terminal fascicles of 3–5 flowers, on a receptacle 3–5 mm wide; perianth 2.5–3.5 mm long, elongate to rhomboid, fleshy, ferruginous when fresh (probably by the trichomes), connate to 1.1–1.7 mm in length, external surface densely pubescent with ferruginous and dendritic trichomes, internal surface densely pubescent (especially in the lobes) with ferruginous trichomes; lobes 3, 1.3–2 × 0.9–1.5 mm and 0.3–0.7 mm thick, without resinous punctuations when rehydrated; stamens 3, the filament column 0.4–0.6 (–0.8) mm long and 0.2–0.4 mm wide, glabrous, straight, not constricted at the apex; anthers 0.6–1 (–1.2) mm long and 0.2–0.3 mm wide; apiculus 0.1–0.2 mm long, acuminate, slightly separated distally. ***Pistillate inflorescence*** and ***flowers*** unknown. ***Infructescence*** ca. 4.5 cm long, with 2 fruits, peduncle ca. 1.7 × 0.5 cm. ***Fruits*** ca. 3.3–4.2 × 2.8–3.1 cm, color not described when fresh, globose, shortly stipitate, densely tomentose, the trichomes dendritic, sessile, ferruginous, the trichomes not falling easily like dust, the surface slightly rugose, the line of dehiscence slightly carinate, the base obtuse, the apex acute; pericarp ca. 3.8–4 mm thick; pedicel ca. 0.6 cm long. ***Seed*** ca. 2.5 × 2.1 cm, the testa when dry brown reddish, slightly ribbed from the base to the apex; aril color when fresh not described, blackish to brown reddish when dry, the texture dry and thin, laciniate almost to the base, in narrow bands distally.

#### Distinctive characters.

*Virolacalimensis* can be recognized by its broad staminate inflorescence, few-flowered inflorescences, elongate to rhomboid, fleshy, densely pubescent perianth, very fleshy filament column and shorter (0.4–0.6 [–0.8] mm long) than the anthers (0.6–1 [–1.2] mm long), and globose, tomentose fruit with trichomes that do not fall easily and with a thick (3.8–4 mm) pericarp (Fig. 6D). Like other species described here, the abaxial side of *V.calimensis*’ leaves are covered with stellate, sessile trichomes whose central portion is darker than the branches on their abaxial surfaces. It further has lateral veins that are well separated (1.3–2.3 cm spaced) (Fig. 4D) and a relatively long and thick petiole.

#### Etymology.

The specific epithet of the new species refers to the Bajo Calima region (Valle del Cauca department, Colombia), the region where most specimens of this new species come from.

#### Distribution.

*Virolacalimensis* occurs in the Pacific coast of Colombia (Valle del Cauca department) and Ecuador (Esmeraldas province) at elevations ranging from 5 to 260 m (Fig. 18C). According to the field notes of the specimens collected in Colombia (e.g., *M. Monsalve 158*), the species grows in nutrient poor soils with a high concentration of aluminum. See Gentry (1986), Faber-Langendoen and Gentry (1991) and Marcano-Berti and Aymard C. (2021) for more information about this region.

#### Phenology.

Staminate flowers of *Virolacalimensis* have been recorded in March, May, June, July, and September. Fruits have been observed in June and August. Pistillate flowers were not seen in the studied material.

#### Common name and uses.

Cuangare (Colombia; *M. Monsalve 158*).

#### Preliminary conservation status.

*Virolacalimensis* is Endangered following IUCN criteria B1a and B2a. It is known from three localities, has an EOO of 313 km^2^, and an AOO of 12 km^2^. While its small range justifies this preliminary status, *V.calimensis* benefits from growing in regions with relatively low rates of deforestation compared to the rest of the region (Antonelli 2022), which is in part due to collective land titling by Afro-Colombian communities in Valle del Cauca, Colombia (Vélez et al. 2020) and proximity to the Awá Reserve (Oviedo 2006).

#### Discussion.

Herbarium specimens of *Virolacalimensis* were previously identified as *V.calophylla* and/or *V.macrocarpa*. This misidentification was probably due to characteristics shared with *V.calophylla*: stellate, sessile trichomes and lateral veins that are well separated, as well as their lax, few-flowered staminate inflorescences that are relatively wide (these traits are unknown in *V.macrocarpa*). However, *V.calimensis* differs from *V.calophylla* in having fleshier, longer staminate perianth with indument on the internal surface and a filament column that is shorter than the anthers. The new species also differs in the size and shape of its fruit as compared to *V.calophylla* and *V.macrocarpa*). A summary of the characteristics that differentiate these three species is presented in Table 4, and 5.

**Table 4.** Comparison of *Virolacalimensis*, with *V.macrocarpa*. ^†^From Smith and Wodehouse (1938).

Morphological character	*V.calimensis*	*V.macrocarpa*
Leaf blades size, and pubescence abaxially	19–25 × 7.3–11 cm; densely pubescent (Fig. 4D)	20–40 × 7–11 cm^†^; sparsely pubescent (Fig. 4J)
Spaced lateral veins	1.3–2.3 cm apart	1.7–3 cm apart
Fruit shape, and long	Globose, 2.8–3.1 cm (Fig. 6D)	Ellipsoid, (2.7–) 3.5–4.5 cm (Fig. 6H)
Pericarp thickness	ca. 3.8–4 mm	(2.7–) 3–4.7 mm
Seed size	ca. 2.5 × 2.1 cm	2.2–2.5 × 1.5–1.7 cm^†^
Habitat	Lowland rain forest, Colombia (Valle del Cauca), and Ecuador (Esmeraldas), at 20 to 260 m elevation	Montane forests, Andes of Colombia (Boyacá), at 1100 m elevation^†^

#### Notes.

The first collection of *Virolacalimensis* was made 76 years ago by José Cuatrecasas (*J. Cuatrecasas 17540*; 5–15 May 1944) in Río Cajambre, Valle del Cauca, Colombia. Six years later, Smith (1950) attributed this Cuatrecasas collection to *V.macrocarpa*, which he used to describe staminate inflorescences; however, he expressed doubt in this and stressed the need for more material, stating that this collection “*has leaves considerably smaller*, *although similar in texture, shape, and indument*.” Smith (1950) refers to two additional collections from Colombia with very young inflorescences, which we could not study, as *V.macrocarpa*; these are *J. Cuatrecasas 15596, 16613* (A, F [as Ch]) from the Western Cordillera (1250–1400 m elevations) and Chocó region (5–50 m elevations) respectively.

**Table 5.** Comparison of *Virolacalimensis*, with *V.calophylla*.

Morphological character	*V.calimensis*	*V.calophylla*
Leaf blade size, and base	19–25 × 7.3–11 cm, obtuse	(15–) 20–60 × 10–16 cm; (usually) deeply cordate to truncate (obtuse)
# of lateral veins	ca. 12–13	11–28
Length of staminate inflorescences	6–8 cm	6–30 cm
Staminate perianth long, and internal surface	2.5–3.5 mm; densely pubescent	1–2.1 mm; glabrescent
Long filament column	0.4–0.6 (–0.8) mm	0.2–0.6 mm
Long anthers	0.6–1 (–1.2) mm	0.4–0.5 mm
Fruit size	ca. 3.3–4.2 × 2.8–3.1 cm (Fig. 6D)	2.5–3 × 1.6–1.5 cm (Fig. 6E)

Walker and Walker (1979; fig. 44) illustrated the pollen of the first collection of this species (again, *J. Cuatrecasas 17540*; 5–15 May 1944), then attributed to *V.macrocarpa*. It was assigned to Pollen Type I (more information can be found in their publication).

The specimens *C. Jativa & C. Epling 1143* and *M. Monsalve 158*, mentioned under *V.macrocarpa* in *Flora of Ecuador* (Jaramillo et al. 2004) and *La Familia de Árboles Tropicales Myristicaceae en el Departamento del Valle del Cauca, Colombia* (Taylor and Devia Álvarez 2000) correspond to this new species. The digital images of *W. Devia et al. 5091* (st), *5461* (st), all at TULV, appear to correspond with *V.calimensis* as well.

#### Specimens examined.

**Colombia. Valle del Cauca**: Bajo Calima, 15–20 m, 28 Jun 1961 (fr), *I. Cabrera 556* (COL);

Costa del Pacífico, Río Cajambre, Silva, 5–80 m, 5–15 May 1944 (♂ fl), *J. Cuatrecasas 17540* (A!, F [image!]); Buenaventura, Carton de Colombia, Vía a Malaga Km. 22, Frente Hans, [03°59'47"N, 076°58'28"W], 20 m, 01 Mar 1990 (fl bud), *W. Devia 3086* (MO-2 sheets!, TULV [image!], UPCB [n.v]); B/ventura [Buenaventura], Canalete, km 28 vía Málaga, 50 m, [s.d.] May 1991 (fr), *W. Devia 3253* (NY!); Bajo Calima, concesión Pulpapel/ Buenaventura, 03°55'N, 077°00'W, 100 m, 11 Mar 1986 (♂ bud fl), *M. Monsalve 968* (INPA [image!], MO!); ibid., 22 Sep 1987 (♂ bud fl), *M. Monsalve 1769* (F [image!], INPA [image!], MO!). **Ecuador. Esmeraldas**: At Tobar Donoso, junct. of Río San Juan and Río Camumbi, [01°11'24"N, 078°30'30"W], 260 m, 25 Jul 1966 (♂ fl), *C. Jativa & C. Epling 1129* (NY!); ibid., 27 Jul 1966 (♂ fl), *C. Jativa & C. Epling 1143* (MO!, NY!, US [image!]).

### ﻿5. Virola cogolloi

Taxon classificationPlantaeMagnolialesMyristicaceae

D. Santam. sp. nov.

CB3A6579-FD71-53CB-86AA-0641E25F4D1E

urn:lsid:ipni.org:names:77298664-1

#### Type.

**Colombia. Antioquia**: Parque Nacional Natural “Las Orquídeas”, Sector Cruces, margen derecha del Río Calles, camino de Cruces hacia Venados, 06°30'N, 076°19'W, 840–870 m, 23 Feb 1989 (♂ fl), *Á. Cogollo 4198* (holotype: MO! [accession 4235971, barcode MO-2560852]; isotypes: COL [n.v.; accession 333167]). Fig. 10

#### Diagnosis.

*Virolacogolloi* previously confused with *V.macrocarpa* both from Colombian montane forests. These species have similar size and shape of the fruits and abaxial leaf blades with stellate, sessile trichomes. Morphologically, it differs from *V.macrocarpa* in leaf blade densely pubescent abaxially (vs. sparsely pubescent), small fruits (2.7–3.2 cm long vs. [2.7–] 3.5–4.5 cm), cover with persistent trichomes (vs. caducous trichomes), and thinner pericarp (1.9–2.1 mm vs. [2.7–] 3–4.7 mm).

***Tree*** (7–) 14–20 m × (8–) 17–24 cm diameter, inner bark not described. ***Exudate*** watery, oxidizing reddish, location of exudate on plant not stated. ***Twigs*** 0.43–0.54 cm thick, terete or laterally compressed, tomentose, trichomes dendritic, sessile, ferruginous, without lenticels on young twigs, but present on mature twigs. ***Leaves*** young terminal bud 2 × 0.4 cm; petiole 1.7–2.4 × (0.28–) 0.32–0.6 cm, slightly to deeply canaliculate, very often shortly winged, glabrescent to tomentulose, the trichomes dendritic; leaf blades 25–29.7 × (9.2–) 10.8–15 cm, widely elliptical; adaxial surface of mature leaves drying blackish to dark brown, the surface smooth, sometimes shiny, glabrous; abaxial surface drying brown, densely pubescent, the trichomes stellate, ca. 0.1 mm diameter, sessile, the central part of the trichome reddish to dark reddish, the branches reddish to brown-reddish; lateral veins ca. 12–14 per side, 3 veins per 5 cm, spaced 1.9–2.5 cm apart, on adaxial side the same color as the adaxial surface, flat to slightly raised, on abaxial surface blackish, raised, puberulent to glabrescent above, densely pubescent to the sides, arcuate-ascending, slightly anastomosing near the margin and without forming a very marked intramarginal vein; tertiary veins very slightly visible on both sides; midvein adaxially flat to slightly elevated, abaxially raised, rounded to laterally compressed, tomentose to puberulent, more pubescent to the sides; base obtuse, not revolute, flat; margin flat; apex acute or obtuse. ***Staminate inflorescence*** 7.5–9 cm long, axes flattened, tomentose, trichomes dendritic, ferruginous; peduncle 2.3–2.5 × 0.32–0.37 cm; main axis with 4–6 ramifications, the first pair opposite to subopposite, the others alternate; bracts not seen. ***Staminate flowers*** (in bud) in lax terminal fascicles of 5–8 flowers, on a receptacle 1.6–2 mm wide; perianth 3.5–4.1 mm long, elliptic to elongate, fleshy, yellowish when fresh, connate by 1.5–2.3 mm long, external surface densely pubescent with ferruginous and dendritic trichomes, internal surface densely pubescent (especially in the lobes); lobes 3 (4), 1.8–2 × 0.6–0.9 (–1.4) mm and 0.2–0.3 (–0.5) mm thick, without resinous punctuations when rehydrated; stamens 3, the filament column 1.2–1.4 mm long and 0.2–0.3 (–0.5) mm wide, glabrous, straight to bottle-shaped, constricted at the apex; anthers 1.1–1.2 mm long, ca. 0.4 mm wide; apiculus 0.2–0.3 mm long, acuminate, slightly separated distally. ***Pistillate inflorescence*** and ***flowers*** unknown. ***Infructescence*** 6–6.5 cm long, with 1–3 fruits, peduncle 2–3.4 × 0.75 cm. ***Fruits*** 2.7–3.2 × 1.7–2.2 cm, when fresh brown or ferruginous (probably by the trichomes), or green and covered with brown trichomes, subglobose to ellipsoid, sessile or shortly stipitate, densely tomentose, persistent, the trichomes dendritic, sessile, ferruginous, the trichomes not falling easily like dust, the surface smooth to slightly rugose, the line of dehiscence smooth, the base obtuse, the apex obtuse to acute; pericarp 1.2–1.6 mm thick on the thinnest side, 1.9–2.1 mm thick on the thickest side; pedicel 0.5–0.8 cm long. ***Seed*** 2.2–2.4 × 1.3–1.4 cm, the testa brown to brown reddish when dry, slightly ribbed distally; aril color when fresh described as red or creamy, brown to brown reddish when dry, the texture dry and thin, laciniate almost to the base, in narrow bands distally.

#### Distinctive characters.

*Virolacogolloi* is best distinguished by the combination of its wide leaf blades that are abaxially covered with dense but inconspicuous stellate and sessile trichomes and with lateral veins that are well-separated (Fig. 4G); wide staminate inflorescences with few flowers per fascicle; and staminate flowers with fleshy perianth lobes that are internally covered by a dense layer of trichomes (especially on the lobes) and filament columns that are longer (1.2–1.4 mm long) than the anthers (1.1–1.2 mm long). It is also distinctive for its densely tomentose fruits with relatively thin pericarp (1.2–1.6 mm thick, the thinnest side) (Fig. 6G) and a seed covered by a thin aril that is laciniate almost to the base.

#### Etymology.

The specific epithet honors Álvaro Cogollo Pacheco, the Colombian botanist who collected most known specimens of this new species as well for his valuable contribution to our knowledge of the Colombian flora. We celebrate his important contributions to botany, epitomized by his numerous collections, ~48 of which now represent type specimens (Tropicos 2021). Álvaro is author of the Myristicaceae treatment for the *Catálogo de las Plantas Vasculares de Antioquia* (Cogollo 2011), among others.

#### Distribution.

*Virolacogolloi* is known only from Colombia (Antioquia department) (Fig. 18C). It is found between 840–1500 m elevation in premontane forest in Las Orquídeas National Park in the Western Cordillera of Colombia.

#### Phenology.

Specimens with staminate flowers of *Virolacogolloi* were collected in February and May. Fruits have been observed in February, April to July. Pistillate flowers were not seen in the studied material.

#### Common name and uses.

Sebo (Colombia; *Á. Cogollo et al. 4195*).

#### Preliminary conservation status.

*Virolacogolloi* is Endangered following IUCN criteria B1a and B2a. It is known from three localities, has an EOO of 24 km^2^, and an AOO of 24 km^2^. While *V.cogolloi* benefits from its occurrence within Las Orquídeas National Park, this region (including within the national park) is still vulnerable to deforestation to expand human activities, including agriculture and livestock grazing (González-Caro and Vásquez 2017; Pedraza-Peñalosa 2015).

#### Discussion.

Prior to our study, most of the specimens with fruits of *Virolacogolloi* were identified as *V.macrocarpa*. Both species grow in Colombian montane forests, respectively in the departments of Antioquia (840–1500 m elevation) and Boyacá (1100 m elevation). In addition to a shared habitat, these species have similar size and shape of the fruits and, like other species described here, abaxial leaf blades with stellate, sessile trichomes with a reddish central portion. However, *V.cogolloi* is distinguished by the size of its leaf blades, petiole thickness, separation of the lateral veins, and other features summarized in Table 6.

**Table 6.** Comparison of *Virolacogolloi*, with *V.macrocarpa*. ^†^From Smith and Wodehouse (1938).

Morphological character	*V.cogolloi*	*V.macrocarpa*
Petiole	1.7–2.4 × (0.28–) 0.32–0.6 cm	1.5–2.3^†^ × 0.18–0.23 cm
Leaf blade size, and pubescence abaxially	25–29.7 × (9.2–) 10.8–15 cm; densely pubescent (Fig. 4G)	20–40 × 7–11 cm^†^; sparsely pubescent (Fig. 4J)
Spaced lateral veins	1.9–2.5 cm apart	1.7–3 cm apart
Fruit size, and pubescence	2.7–3.2 × 1.7–2.2 cm; with persistent trichomes (Fig. 6G)	(2.7–) 3.5–4.5 × (1.9–) 2.3–2.9 cm; with caducous trichomes (Fig. 6H)
Pericarp thickness	1.9–2.1 mm (on the thickest side)	(2.7–) 3–4.7 mm

A second *Virola* species (*V.tuckerae*, formally described below) occurs with *V.cogolloi*. In addition to their similar distributions, these species share leaf blades that are densely pubescent abaxially (Fig. 4G, N). However, *V.tuckerae*, has narrower leaf blades with more lateral veins, a filament column that is shorter than the anthers, and a fruit that is covered by a dense layer of trichomes. A comparison between these two species is presented in Table 7.

**Table 7.** Comparison of *Virolacogolloi*, with *V.tuckerae*.

Morphological character	*V.cogolloi*	*V.tuckerae*
Leaf blade wide	(9.2–) 10.8–15 cm	5–7.2 (–11.7) cm
Lateral veins	ca. 12–14 per side, 3 veins per 5 cm, spaced 1.9–2.5 cm	16–19 per side, 3–4 (–6) veins per 5 cm, spaced 1.1–1.8 (–2.1) cm
Staminate peduncle	2.3–2.5 cm long	0.6–1.8 (–2.8) cm long
Filament column	1.2–1.4 mm long	0.6–0.7 (–0.9) mm long
Anthers	1.1–1.2 mm long	1.2–1.6 mm long
Fruit pubescence	With an inconspicuous layer of trichomes, persistent (Fig. 6G)	With a conspicuous layer of trichomes, caducous, that fall like dust (Fig. 6N)
Pericarp thickness	1.9–2.1 mm (on the thickest side)	ca. 2.4 mm

#### Notes.

The specimens *Á. Cogollo et al. 4195*, cited as *V.macrocarpa* in Cogollo (2011), and [*Á.*] *Cogollo 3331*, included in Gradstein (2016), both cited as *V.macrocarpa*, correspond with this new species.

#### Specimens examined.

**Colombia. Antioquia**: Urrao, Parque Nacional Natural “Las Orquídeas”, Vereda Cruces, camino a Piñares, poco después de la escuela La Esperanza, orilla izquierda del río Calles, 06°28'56"N, 076°19'20"W, 960 m, 5 May 2013 (♂ fl), *J. Betancur et al. 18081* (COL!, NY!); Parque Nacional Natural “Las Orquídeas”, Sector Calles, quebrada La Bironda, 06°31'N, 076°19'W, 1300–1500 m, 02 Apr 1992 (fr), *D. Cárdenas & E. Alvarez 3239* (MO!); Parque Nacional Natural “Las Orquideas”, Camino a San Mateo, margen izquierda de la Quebrada San Mateo, 06°33'N, 076°19'W, 1060 m, 07 Jun 1988 (fr), *Á. Cogollo et al. 3331* (JAUM!, MO!); Parque Nacional Natural “Las Orquídeas”, sector Venados arriba, margen izquierda del río Venados, 06°34'N, 076°19'W, 1110–1240 m, 27 Jul 1988 (fr imm), *Á. Cogollo et al. 3553* (COL!, JAUM-2 sheets!, MO!); Parque Nacional Natural “Las Orquídeas”, Sector Cruces, margen derecha del río Calles, camino de Cruces hacia Venados, 06°30'N, 076°19'W, 840–870 m, 23 Feb 1989 (fr), *Á. Cogollo et al. 4195* (COL!, JAUM!, MO!); Parque Nacional Natural Las Orquídeas, Vereda Cruces, sitio Piñares, camino a Perdidas, poco después de la escuela La Esperanza, 06°28'35.5"N, 076°19'39.5"W, 980 m, 3 May 2013 (fr), *S. E. Hoyos et al. 2254* (COL!).

### ﻿6. Virola cumala

Taxon classificationPlantaeMagnolialesMyristicaceae

D. Santam. sp. nov.

65E558C6-A5BD-56A9-B118-3B820488EF64

urn:lsid:ipni.org:names:77298665-1

#### Type.

**Figure 11.**
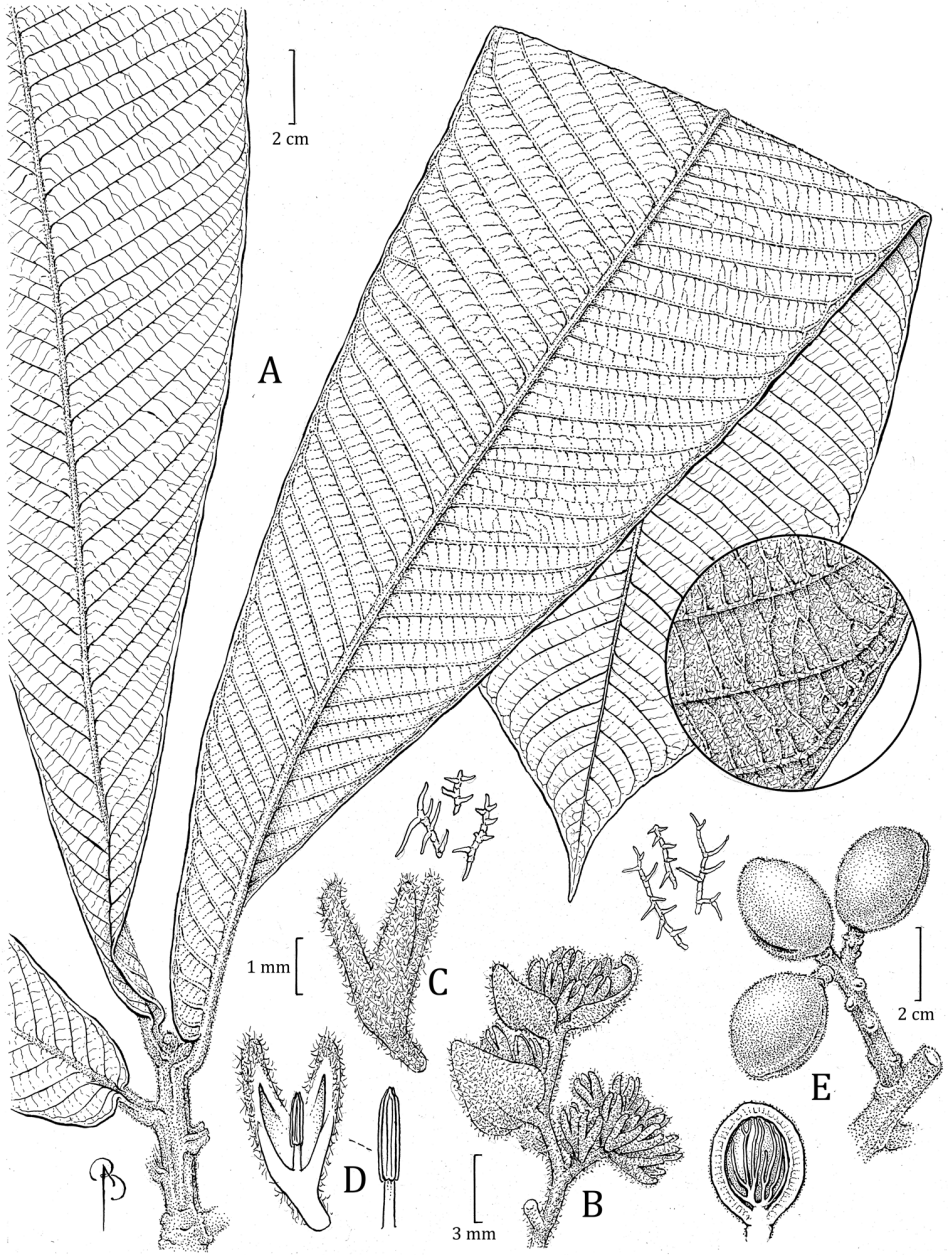
*Virolacumala***A** branch with leaf blades, with detail of abaxial surface showing trichomes and tertiary venation **B** part of staminate inflorescence **C** lateral view of staminate perianth with detail of trichomes (top) **D** medial section of staminate flower and androecium (right) **E** fruits with detail of trichomes (left), and open fruit showing seed covered with laciniate aril. Drawn by Bobbi Angell based on *R. Vásquez & C. A. Grández 17532* (**A–D** MO), and *N. Jaramillo et al. 388* (**E** MO).

**Peru. Amazonas**: Bagua, distrito Imaza, Región Nororiental del Marañón, Comunidad de Kampaenza, Ribera de la quebrada Shimutaz, Río Marañón, 04°55'S, 078°19'W, 320 m, 09 Sep 1994 (fr), *N. Jaramillo, R. Apanu, G. Apanu 388* (holotype: MO! [accession 5096935, barcode MO-254989]; isotypes: UPCB [n.v.]). Fig. 11

#### Diagnosis.

*Virolacumala* is most similar to *V.decorticans* and *V.multinervia*; all these species have large leaves, numerous lateral veins, dense indument of dendritic brown to ferruginous trichomes in almost all parts of the plant, and large fruits. Morphologically, it differs from *V.decorticans* in having leaf blade on upper surface glabrous (vs. pubescent), the staminate perianth is subcarnose, without lines or dots (vs. submembranous, and with lines), long filament column (0.5–0.7 mm vs. 0.3–0.4 mm), and anthers (0.7–0.9 mm vs. 0.5–0.6 mm), and narrow fruits (2.3–2.9 cm vs. 1.7–2.2 cm). It differs from *V.multinervia* in the staminate perianth subcarnose (vs. membranous), densely pubescent on all the surface (vs. glabrous or pubescent only at the base and apex), long anthers (0.7–0.9 mm vs. 0.3–0.4 mm), and large fruit (2.3–2.9 cm vs. 1.5–2.5 cm).

***Tree*** 15–35 m × 38 cm diameter; ***inner bark*** and ***exudate*** not described. ***Twigs*** 0.52–1.2 cm thick, laterally flattened to angled, densely tomentose, trichomes dendritic, branched from the base (1–2 mm long) with short lateral branches (0.1–0.2 mm long), brown, sometimes the bark in mature twigs cracks and flakes in small pieces. ***Leaves*** young terminal bud 2–2.5 × 0.6–2 cm; petiole 0.9–2.2 × 0.43–0.9 cm, strongly canaliculate, densely pubescent, trichomes dendritic; leaf blades 33.1–50.7 (–56.5) × (9.5–) 11.7–15.5 cm, elliptic-oblong to elliptic to obovate, sometimes gradually tapering towards the base; adaxial surface of mature leaves (usually) drying brown to dark grayish, glabrous, smooth; abaxial surface drying brown to pale brown, densely pubescent, the trichomes dendritic, yellowish to pale brown, shortly pediculated (branched almost at the base), with 5–8 branches, the branch 0.3–0.7 mm long, persistent; lateral veins 49–65 per side, with 5–7 (–11) veins per 5 cm, spaced 0.4–1.1 cm apart, the same color as the adaxial surface or sometimes slightly contrasting in color, impressed, on abaxial surface the same color as the surface, very conspicuous and raised, slightly straight to arcuate distally, anastomosing very near the margin, forming an intramarginal vein; tertiary veins slightly prominent adaxially, very prominent abaxially; midvein adaxially flat, pubescent, abaxially raised, rounded, pubescent; base cordate to subcordate, not revolute, flat; margin flat; apex acute to cuspidate. ***Staminate inflorescences*** 7.5–12 cm long, axes slightly flattened at the apex of the defoliated branches, densely pubescent, the trichomes dendritic (1–1.6 mm long), ferruginous; peduncle 1.3–1.5 × 0.34–0.47 cm; bracts 0.4–0.7 × 0.2–0.4 cm, ovate, sometimes triangular, with 3 vertical lines, pubescent on both sides, caducous. ***Staminate flower*** not very dense, terminal fascicles of 13–21+ flowers, on a receptacle ca. 2 mm wide; pedicel 1.5–2 mm long, pubescent; perianth 2–3 mm long, elongate, subcarnose, brown when fresh, connate to 0.6–1 mm in length, external surface densely pubescent with brown trichomes, internal surface glabrous; lobes 3, 1.1–1.5 × 0.4–0.6 mm, 0.1–0.2 mm thick, without resinous punctuations when rehydrated; stamens 3, the filament column 0.5–0.7 mm long and 0.1–.0.2 mm wide, thin, straight, not constricted at the apex; anthers 0.7–0.9 mm long, and ca. 0.2 mm wide; apiculus inconspicuous or obtuse. ***Pistillate inflorescences*** and ***flowers*** not seen. ***Infructescence*** 3.5–5.5 cm long, with 1–3 fruits, peduncle 1–2.7 × 0.4–07 cm. ***Fruits*** 3–3.5 × 2.3–2.9 cm, brown (possibly by the indumentum) when fresh, rounded to ellipsoid, densely and persistently pubescent with a layer of trichomes of 1–2.3 mm thick, the trichomes dendritic, brown to ferruginous, the surface not seen, the line of dehiscence not carinate, the base and apex obtuse; pericarp 3–6 mm thick; pedicel ca. 0.5 mm long. ***Seed*** (1.9–) 2.3–2.5 × (0.8–) 1.1–1.9 cm, the testa pale brown to brown reddish when dry, grooved (*N. Jaramillo 550*), aril when fresh not described, brown to blackish when dry, the texture dry and thin, laciniate in very narrow bands, almost to the base.

#### Distinctive characters.

**Figure 12.**
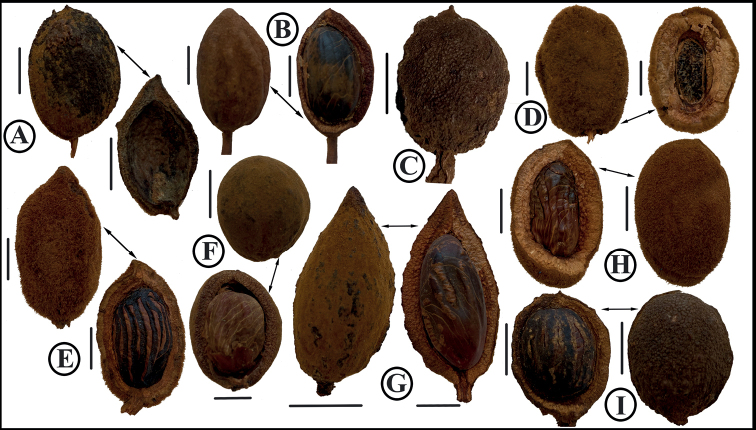
Diversity of fruits from *Virola* species that have numerous and close lateral veins, notice the shape, indument, and pericarp thickness **A***V.caducifolia* (*W. Rodrigues & D. Coêlho 8700*, MO) **B***V.chrysocarpa* (*B. Hammel et al. 16864*, MO) **C***V.crebrinervia* (*N. A. Rosa 4446*, NY) **D***V.cumala* (*C. Grá[n]dez et al. 4919*, MO) **E***V.decorticans* (*C. A. Cid Ferreira et al. 9961*, MO) **F***V.koschnyi* (*L. D. Gómez et al. 22725*, MO) **G***V.megacarpa* (*G. de Nevers 5184*, MO) **H***V.multinervia* (*C. A. Sothers & E. D. C. Pereira 1069*, MO) **I***V.multicosta* (*R. Vásquez & C. Grández 17507*, MO). Scale bars: 1 cm.

The most distinctive characteristic of *Virolacumala* is the dense indument of dendritic brown to ferruginous trichomes that cover almost all parts of the plant. Vegetatively, this species also has large leaves with numerous lateral veins that are prominent (including the tertiary veins, especially below), thick twigs, and bark that sometimes cracks and flakes into small pieces. Further, the staminate flowers have a subcarnose perianth that is densely and uniformly pubescent outside and glabrous inside with narrow lobes, a filament column that is straight and similar in length to the anthers, and large fruits that are covered with a thick layer of trichomes, thick pericarp, and a seed covered by an aril that is laciniate in very narrow bands almost to the base (Fig. 12D).

#### Etymology.

The specific epithet is taken from the common name on the label of *R. Vásquez & N. Jaramillo 7879*, MO). This common name is often used in Peru to refer to various species of *Virola*, as well to other species in *Otoba* (A. DC.) H. Karst., *Iryanthera* (A. DC.) Warb., and *Osteophloeum* Warb. (e.g. Vásquez Martínez 1997; Zárate-Gómez et al. 2019).

#### Distribution.

*Virolacumala* is known only from Peru (in the Amazonas, Loreto, and Pasco departments) (Fig. 18C). It occurs in primary forests in non-inundated, lateritic soil. It ranges in elevation from 120–320 m, although one collection from Pasco reaches 700–800 m (*H. van der Werff 20095)*. It is possible that this species also occurs in Ecuador, as some collections were made close to the border of Peru and Ecuador.

#### Phenology.

Staminate flowers of *Virolacumala* have been recorded in July; pistillate flowers were not seen in the studied material. Fruits have been collected in May, August to October, and December.

#### Common name and uses.

Cumala negra (Peru; *R. Vásquez & N. Jaramillo 7879*, MO).

#### Preliminary conservation status.

*Virolacumala* is Endangered following IUCN criterion B2a. It is known from five localities, has an EOO of 219,024 km^2^, and an AOO of 20 km^2^. Justifying its status, the western Amazonian region where this species occurs is modeled to be modestly impacted by new road construction, a threat that is likely to be superseded by the development of oil palm plantations in the region (Arima 2016).

#### Discussion.

*Virolacumala* is found among a morphological group of *ca.* 17 species (personal count) that correspond to the group Rugolosae of Smith and Wodehouse (1938). Generally, this group is characterized by leaves with numerous, closely adjacent lateral veins, percurrent tertiary veins that are usually conspicuous, cordate bases, and dendritic trichomes; staminate inflorescences often with large bracts that cover flowers; staminate flowers that are small and usually densely aggregated with membranous to sub membranous perianth, the tube almost split to the base, and a slender filament column. Within this group, the new species is most similar to *V.decorticans* Ducke and *V.multinervia* Ducke, both from South America, and *V.megacarpa* A. H. Gentry from Panama. All four species have large leaves, numerous lateral veins, dense indument of dendritic brown to ferruginous trichomes in almost all parts of the plant, large fruits with a thick layer of trichomes (thin in *V.megacarpa*), and thick pericarp. *Virolacumala* further shares bark that cracks and flakes into small pieces and the thick young terminal bud of leaves. These species are distinguished by the characteristics presented in Table 8.

**Table 8.** Comparison of *Virolacumala*, with three other morphologically closely related *Virola* species. ^‡‡^From Rodrigues (1980). Perianth information for *V.decorticans* is from *C. Grández et al. 2525*, MO), and fruits from *C. A. Cid Ferreira 7307, 9961*, MO); while those of perianth, filament column and anthers presented for *V.multinervia* are from *C. A. Cid Ferreira et at. 7537*, MO; *W. Rodrigues & D. Coelho 9617*, MO; *J. E. L. S. Ribeiro 1249*, MO; and *W. Thomas et al. 5239*, MO).

Morphological character	*V.cumala*	*V.decorticans*	*V.megacarpa*	*V.multinervia*
Leaf blade upper surface	Glabrous	Pubescent	Glabrous	Glabrous
No. of lateral veins	49–65	45–60^‡‡^	(32–) 40–50	40–60^‡‡^
Staminate perianth texture, and pubescence on external surface	Subcarnose, without lines or dots, densely pubescent on all the surface	Submembranous, with lines, pubescent	Subcarnose, without lines or dots, densely pubescent on all the surface	Membranous, with lines, glabrous or pubescent only at the base and apex
Filament column length	0.5–0.7 mm	0.3–0.4 mm^‡‡^	0.9–1.3 mm	0.3–0.5 mm
Anther length	0.7–0.9 mm	0.5–0.6 mm^‡‡^	0.8–0.9 mm	0.3–0.4 mm
Fruit size and apex	3–3.5 × 2.3–2.9 cm, obtuse (Fig. 12D)	2.7–3.5 × 1.7–2.2 cm^‡‡^, acute to apiculate (Fig. 12E)	4–5.7 × 2–2.9 cm, acuminate to rostrate (Fig. 12G)	2–3 × 1.5–2.5 cm, apiculate^‡‡^ (Fig. 12H)
Pericarp thickness	3–6 mm	4–5 mm	3–6 mm	1.5–4 mm

*Virolacumala* can be distinguished from other species with numerous lateral and relatively close veins (e.g., *V.caducifolia* W. A. Rodrigues, *V.chrysocarpa* D. Santam. & Aguilar, *V.flexuosa* A. C. Sm., *V.guggenheimii* W. A. Rodrigues, *V.koschnyi* Warb. and *V.polyneura* W. A. Rodrigues) by its fruits: these are large (3–3.5 × 2.3–2.9 cm vs. 1.6–3 × 1.3–2.5 cm), covered with a thick layer of trichomes (vs. tomentose or glabrescent), and have a thick pericarp (3–6 mm vs. 1–4 mm).

The fruits of *Viroladivergens*, *V.loretensis* A. C. Sm., and *V.mollissima* (species of Mollissimae group in Smith and Wodehouse 1938) are also covered by a dense layer of trichomes that is similar to *V.cumala* (cf. Fig. 3E). However, these species have fewer lateral veins (15–30 vs. 49–65), anthers with an apiculate apex (vs. inconspicuous or obtuse), and thin pericarp (0.3–0.7 vs. 3–6 mm).

#### Notes.

The type specimen of *V.cumala* (*N. Jaramillo et al. 388*, MO) was cited as *V.multinervia* in Vásquez et al. (2018). Peruvian specimens identified as *V.multinervia* are very variable, likely representing a combination of misidentifications and undescribed species. Within these, we identified three morphologically distinct groups. The first group corresponds to *V.cumala*. The second, represented by the fruiting specimens *A. H. Gentry et al. 42594* (MO), *C. Grández et al. 4929* (MO), *R. Vásquez & C. Grández 17507* (MO-2 sheets), we tentatively identify as *V.multicostata* Ducke (due to lack of flowers). This group is characterized by scant pubescence covering twigs, petioles, and axes of the infructescence, large leaf-blades (28–37 × 8.1–10 cm) with numerous lateral veins (54–62), the tertiary veins prominent below, and inconspicuously pubescent to glabrescent fruits that are 2.1–2.3 × 1.5–1.8 cm (Fig. 12) with a rugose surface and thin pericarp (1.3–1.5 mm). Finally, the third group is represented by the specimens *A. H. Gentry et al. 19014* (♂ fl.), *A. H. Gentry et al. 21736* (very young fl. bud.; F [image], MO!), and *R. Vásquez & N. Jaramillo 9379* (♀ fl; MO!). This group is characterized by deciduous (i.e. all leaves fall off the tree), oblong leaves (32.7–44 × 8.5–12.6 cm), brown trichomes that cover the entire plant, staminate inflorescences with bracts that are 0.9–1.5 × 1–1.2 cm, staminate flowers with a ca. 2 mm long perianth which is connate to 0.5 mm in length and densely pubescent externally, glabrous internally, and without dots or lines when rehydrated, perianth lobes that are ca. 1.5 × 0.6 mm, and a filament column ca. 0.4–0.5 mm long with anthers 0.4–0.6 mm long.

#### Specimens examined.

**Peru. Amazonas**: Bagua, distrito Imaza, Región Nororiental del Marañón, Comunidad de Kampaenza, Ribera de la quebrada Shimutaz, Río Marañón, 04°55'S, 078°19'W, 320 m, 10 Oct 1994 (fr), *N. Jaramillo & C. Peas 550* (MO!, UPCB [n.v.]). **Loreto**: Yanamono, Explorama Tourist Camp on Río Amazonas between Indiana and mouth of Río Napo, 03°28'S, 072°48'W, 120 m, 26 July 1980 (♂ fl), *A. H. Gentry et al. 29027* (INPA [image!], MO!); ExplorNapo Camp (Río Sucusari), 03°15'S, 072°54'W, 140 m, 29 July 1991 (♂ fl), *R. Vásquez & C. A. Grández 17532* (MEXU [image!], MO-2 sheets); Maynas, IQ [Iquitos]–Nauta, Km 32, 150 m, 20 Aug 1986 (fr), *R. Vásquez & N. Jaramillo 7879* (MO!); Maynas, Distrito Las Amazonas, Explor Napo (Suwzari), 03°20'S, 072°55'W, 124 m, 5 Dec 1992 (fr), *C. Grá[n]dez et al. 4919* (MO!). **Pasco**: Distr. Palcazú, El Paujil, 10°20'12"S, 075°15'39"W, 700–800 m, 13 May 2005 (fr), *H. van der Werff 20095* (F [image!]).

### ﻿7. Virola excisa

Taxon classificationPlantaeMagnolialesMyristicaceae

D. Santam. sp. nov.

D8287A04-9E5A-5B91-B531-749358EB70C2

urn:lsid:ipni.org:names:77298666-1

#### Type.

**Figure 13.**
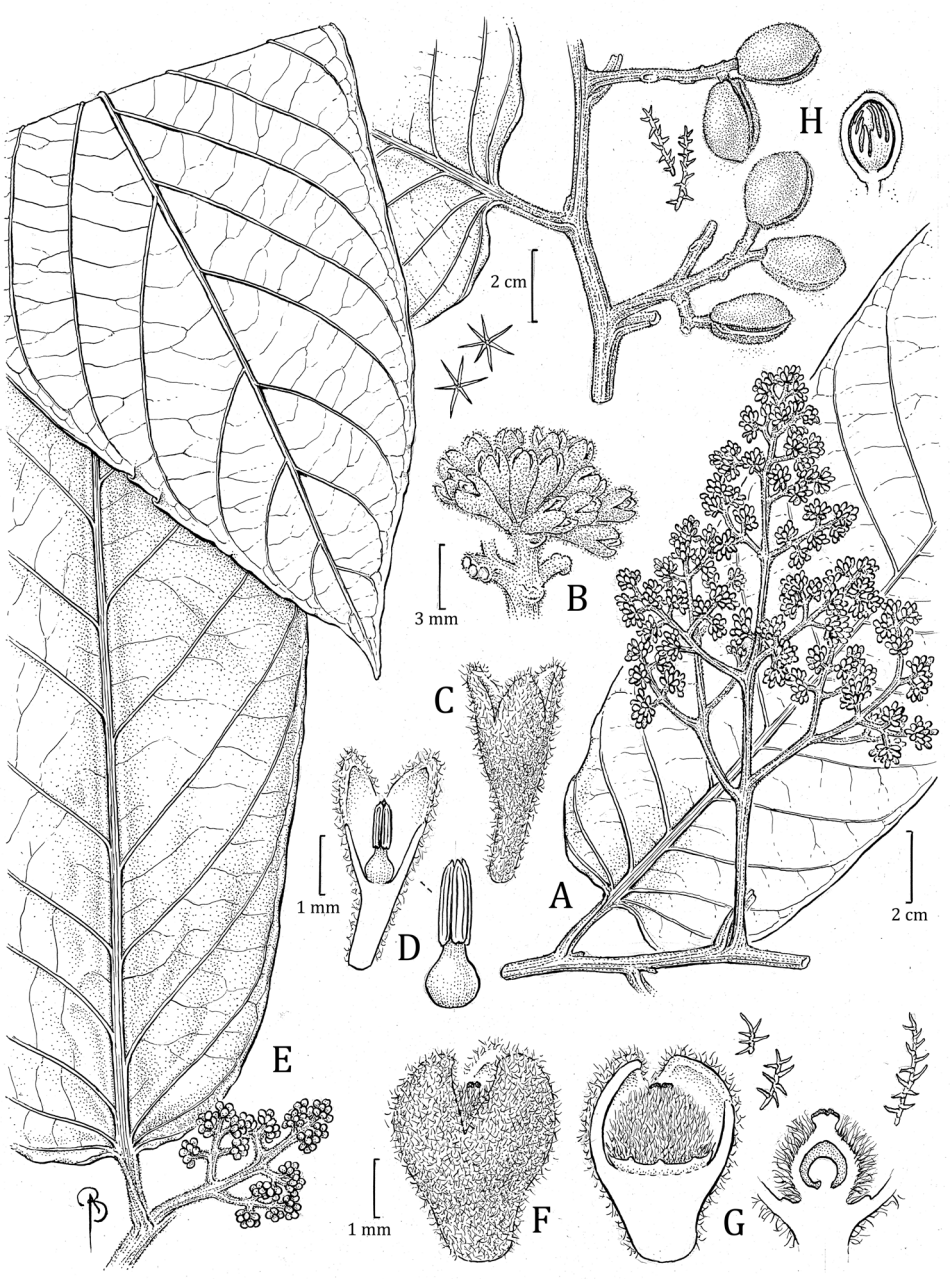
*Virolaexcisa***A** branch with staminate inflorescence **B** partial staminate inflorescence **C** staminate perianth **D** medial section of staminate flower and androecium detail (right) **E** branch with pistillate inflorescence, with detail of trichomes on abaxial surface (right) **F** lateral view of perianth (left) and medial section of pistillate flower (right), with enlargement of trichomes on perianth (above right) **G** medial section of pistillate flower, with an enlargement of trichomes on gynoecium (right) **H** branch with fruits, including an enlargement trichomes (middle left), and an open fruit showing seed covered with laciniate aril (right). Drawn by Bobbi Angell based on *W. Palacios & D. Neill 622* (**A–D** MO), *W. Palacios & C. Iguago 4435* (**E–G** MO), and *W. Palacios 3186* (**H** MO).

**Ecuador. Pastaza**: Río Curaray, dos horas río abajo del pueblo Curaray, en la boca del Río Namoyacu, 01°24'S, 076°45'W, 275 m, 14–18 Aug 1985 (♂ fl), *W. Palacios & D. Neill 622* (holotype: MO! [accession 3482884, barcode MO-1565775]; isotype: F [accession 1989048, barcode V0354346F; image!], INPA [accession 147770; image!], NY!, QCA [n.v.]). Fig. 13

#### Diagnosis.

*Virolaexcisa* is similar to *V.obovata* in the shape of its leaves, which are sparsely stellate and/or dendritic trichomes, or both kinds on abaxial side; staminate flowers with perianth that is nearly glabrous internally and the filament column of similar size to the anthers; and fruits that are covered with a conspicuous layer of ferruginous trichomes. Morphologically, it differs from *V.obovata* in the staminate perianth without resinous punctuations vs. resinous punctuations), long fruits (1.4–1.7 cm vs. 0.8–1 cm), with thick pericarp (1.5–2.3 mm vs. 1–1.2 mm). Given the above mentioned characteristics of leaves and pubescence, *Virolaexcisa* is similar to *V.peruviana*. But it is distinguished by the fruits densely pubescent at maturity (vs. glabrescent), and slightly carinate (vs. conspicuously carinate).

***Tree*** 8–35 m × 20–40 (–60) cm diameter, inner bark described as grayish and rough. ***Exudate*** watery, red, or reddish in the trunk or without specifying from where. ***Twigs*** 0.2–0.47 (–0.55) cm thick, terete, slightly compressed to slightly angulate, tomentose, trichomes dendritic, sessile, ferruginous, without lenticels. ***Leaves*** young terminal bud 1.2–2.2 × 0.3–0.6 cm; petiole 1.3–2 (–3) × 0.28–0.42 cm, slightly canaliculate and winged, often flattened above, tomentose, sometimes glabrescent, the trichomes dendritic; leaf blades (10.5–) 23.5–46.5 × (6.7–) 9.5–13.5 (–19.7) cm, lanceolate to obovate; adaxial surface of mature leaves usually drying brown to dark brown, the surface smooth, sometimes shiny, glabrous; abaxial surface usually drying white grayish, white brownish to pale brown, sparsely pubescent, the trichomes stellate or sometimes mixture of trichomes stellate and dendritic (especially along the veins), the stellate trichomes ca. 0.1–0.2 mm diameter, sessile, the central part of the trichome pale to dark reddish, sometimes colorless, the branches pale reddish or colorless; lateral veins (13–) 18–24 per side, 3–4 veins per 5 cm, spaced 1.3–2.5 (–3.3) cm, on adaxial side the same color as the adaxial surface, flat to slightly raised, on abaxial surface darker than the surface, slightly raised, glabrescent to scattered pubescent, arcuate-ascending, slightly anastomosing near the margin and without forming a marked intramarginal vein; tertiary veins slightly visible on both sides; midvein adaxially flat to slightly elevated, abaxially raised, rounded, tomentose to puberulent, more pubescent to the sides; base truncate to subcordate, rarely deeply cordate, not revolute, flat; margin flat; apex acute to acuminate. ***Staminate inflorescence*** 4.5–15.5 cm long, axes flattened, tomentose, with trichomes dendritic (0.2–0.5 mm long), ferruginous; main axes with 9–15 ramifications, the first pair opposite to subopposite, the second and third ramifications sometimes opposite to subopposite, otherwise alternate; peduncle (1.3–) 1.7–4.3 × (0.13–) 0.21–0.3 cm; bracts 2–2.2 × 1–1.6 mm (measured on immature inflorescences). ***Staminate flowers*** in dense terminal fascicles of 9–25+ flowers, on a receptacle 1.5–1.7 mm wide, sometimes absent; perianth 1.5–2.8 mm long, ovate to obovate, fleshy, cream, yellowish brown, or ferruginous (probably by the trichomes) when fresh, connate 1–1.5 mm long, external surface densely pubescent with ferruginous and dendritic trichomes, internal surface glabrous to almost glabrous; lobes 3, 1–1.1 × 0.5–0.8 mm, and 0.1–0.2 mm thick, without resinous punctuations when rehydrated; stamens 3, the filament column (0.4–) 0.5–0.8 mm long and 0.3–0.4 mm wide, glabrous, fleshy, straight and wide throughout its length, constricted at the apex; anthers 0.5–0.7 mm long, 0.3–0.4 mm wide; apiculus ca. 0.1 mm long, acuminate, separate. ***Pistillate inflorescence*** 4–7 cm long, axes flattened, tomentose, the trichomes dendritic, ferruginous; peduncle 1.5–4.2 × 0.18–0.46 cm; main axes with 9–10 ramifications, the first and second pair (usually) opposite to subopposite, the other alternate; bracts not seen. ***Pistillate flowers*** in terminal fascicles of 3–10+ flowers, on a receptacle 2–4 mm wide; perianth 1.7–2.5 mm long (measurement from immature flower), globose, brown when fresh, connation not seen, external surface densely pubescent with ferruginous and dendritic trichomes, internal surface glabrous; lobes probably 3, not seen properly due to the immaturity of flowers, ca. 0.2–0.3 mm thick, without resinous punctuations when rehydrated; gynoecium 1.1–2 × 1.2–2 mm, globose, densely pubescent, with ferruginous trichomes; stigma 2-lobed, 0.4–0.7 × 0.2 mm, sessile, drying blackish, slightly wavy at the margins. ***Infructescence*** 3.5–7.5 (–11.5) cm long, with 4–7 fruits, peduncle ca. 1.4–3.5 (–5) × 0.3–0.5 cm. ***Fruits*** 2–2.6 × 1.4–1.7 cm, when fresh green or brown (the latter probably due to the trichomes) and covered with brown trichomes, ovoid, without stipe, densely tomentose, the trichomes dendritic (0.1–0.4 mm long), sessile, ferruginous, falling very easily like dust, the surface smooth, the line of dehiscence slightly carinate, the base truncate to obtuse, apex acute; pericarp 1.5–2.3 mm thick; pedicel 0.4–0.7 cm long. ***Seed*** 2–2.3 × 1.1–1.3 cm, the testa drying brown to dark brown, sulcate and bullate; aril when fresh described as red, dark brown, brown-reddish to blackish when dry, the texture dry and thin, laciniate almost to the base, in narrow bands distally.

#### Distinctive characters.

**Figure 14.**
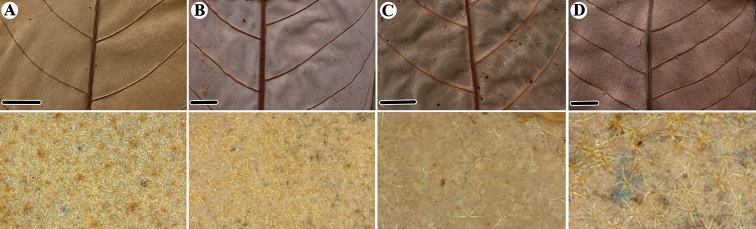
Comparison of abaxial surface, veins, and trichomes of **A***V.calophylla***B***V.excisa***C***V.obovata* and **D***V.peruviana***A** from *J. E. L. S. Ribeiro et al. 1138*, MO **B** from *A. [H.] Gentry & C. Díaz 58536*, MO **C** from *R. Rueda & J. Ruíz 621*, MO **D** from *C. A. Grández & A. Chiquispama 1065*, MO. Scale bars: 1 cm.

*Virolaexcisa* can be recognized by its long, lanceolate to obovate leaves that are truncate to subcordate at the base with an underside that is usually white grayish when dry and sparsely pubescent with stellate sessile trichomes (sometimes with dendritic trichomes along the veins) (Fig. 14B); lateral veins that are well separated, with the marginal and tertiary veins not very conspicuous; staminate flowers with the external perianth covered with dense pubescence of dendritic trichomes and a glabrous internal surface, a fleshy filament column that is similar in length ([0.4–] 0.5–0.8 mm long) to the anthers (0.5–0.7 mm long); fruits that are densely pubescent with trichomes that fall easily like dust; and the seed with a bullate, sulcate testa sulcate (Fig. 6I). Additionally, herbarium specimens are usually aromatic.

#### Etymology.

The specific epithet of the new species comes from the word *excisus*, meaning cut out (Stearn 1992). The makes reference to the fact that several herbarium specimens of this new species come from felled trees that were killed to make way for oil pipes (e.g. *V. Zak & S. Espinoza 4733*, *5149; F. Hurtado 2980*). This has created irreparable damage to nature, destroying the habitat of this and many other species, potentially paving the path toward their extinction.

#### Distribution.

*Virolaexcisa* is known from Colombia (Amazonas, Putumayo departments), Ecuador (Napo, Pastaza, Morona-Santiago provinces), Peru (Amazonas, Huánuco, Loreto, Pasco, Ucayali departments), and Brazil (Acre, Amazonas state) (Fig. 18B), where it occurs in primarily moist tropical forest. Some herbarium labels mention that it grows on hills with red soils, sandy clay soil, near black water or várzea. It ranges in elevation from 130–580 m, with two collections reaching 700 and 830 m.

#### Phenology.

Staminate flowers of *Virolaexcisa* have been collected in February, May to October, and pistillate flowers in May, July to September. Fruits have been collected in January to April, August to December.

#### Common name and uses.

Common names include in Colombia: sangre de toro (*J. Cuatrecasas 10653* [F, without common name at COL]). Ecuador: *gomenkowe* (Wao tededo; Pérez et al. 2014), gunhuékonmo (Huaorani; *E. Gudiño et al. 956*), lugumpapu (Huaorani; *D. Rubio & T. Coba 839*), huapa (Quichua; *E. Gudiño et al. 2139, 2154*), numpa tsempu kumpari (Huambisa; *V. Huashikat 2267, 2322*), puca huapa (Quichua; *H. Vargas 1590*), tsempu (Huambisa; *V. Huashikat 682*). Peru: cumala blanca ([*B.] Kröll 487)*, sempo (Aguaruna; *J. J. Wurdack 2272*). Brazil: ucuhuba ([*W. A.] Ducke 396*), ucuuba (*M. Silveira et al. 840*), ucuúba da folha grande (*M. Silveira et al. 718*). The trunk of *V.excisa* is used for construction of houses, while the aril attracts birds, rodents, and monkeys (Pérez et al. 2014, as *V.obovata*) or the wood used for firewood (*M. Silveira et al. 840*).

#### Preliminary conservation status.

*Virolaexcisa* is Not Threatened following IUCN criteria B1a and B2a. It is known from 25 localities, has an EOO of 810,234 km^2^, and an AOO of 112 km^2^. While this species grows in regions of the world that are threatened by human landscape modification (Antonelli 2022), its distribution is wide enough that this species does not need to be considered threatened currently.

#### Discussion.

Of morphology apparent in herbarium specimens, the leaf blades and some flower and fruit features of *V.excisa* are similar to *V.obovata*, and most of the studied specimens were identified as such in *Flora of Ecuador* (Jaramillo et al. 2004). Both species share similarly shaped leaves that appear to be glabrous abaxially, but actually bear sparsely stellate and/or dendritic trichomes, or both kinds; staminate flowers with perianth that is nearly glabrous internally and the filament column of similar size to the anthers; and fruits that are covered with a conspicuous layer of ferruginous trichomes. Despite these morphological similarities, *V.excisa* differs from *V.obovata* in traits related to the leaf base, the perianth of staminate flowers, and fruit, pericarp, and seed size. Differences between these species are summarized in Table 9. In addition to the features in Table 9, there are distinguishing traits that are difficult to describe properly; compared to *V.obovata*, herbarium specimens of *V.excisa* tend to have more elongate leaves and staminate perianth, fruits usually have more conspicuous carina, and trichomes are denser and fall more easily.

**Table 9.** Comparison of *Virolaexcisa*, with *V.obovata*.

Morphological character	*V.excisa*	*V.obovata*
Leaf blades size, base and shape	(10.5–) 23.5–46.5 × (6.7–) 9.5–13.5 (–19.7) cm; base truncate to subcordate, rarely deeply cordate; lanceolate to obovate	11.5–29.5 (–34) × 6.8–12.5 cm; base attenuate to acute; obovate-elliptic.
Staminate perianth	Without resinous punctuations	With resinous punctuations
Infructescence peduncle	ca. 1.4–3.5 (–5) cm long	0.7–1.3 cm long
Fruits	2–2.6 × 1.4–1.7 cm, without stipe, the base truncate to obtuse (Fig. 6I)	1.3–2.3 ×x 0.8–1 cm, shortly stipitate, the base rounded (Fig. 6J)
Pericarp thickness	1.5–2.3 mm	1–1.2 mm
Seed	2–2.3 × 1.1–1.3 cm	1.7–1.9 × 0.6–0.7 cm

*Virolaexcisa* resembles *V.calophylla* and especially *V.peruviana* in the shape of its leaves (including the base and lateral vein pattern) and long staminate inflorescences with perianth that is glabrous internally. In addition, *V.calophylla* and the new species have similar fruit morphology (at least when young). *Virolaexcisa* can be distinguished from *V.calophylla* by its abaxial leaf blades that are sparsely pubescent and puberulent (vs. densely pubescent and appearing squamose; see Fig. 14A), shorter, narrower staminate inflorescences, shorter perianth, and filament column that is similar in length to the anthers (vs. filament column longer than anthers). Immature fruits of *V.calophylla* are densely pubescent (e.g. *D. Daly et al. 6773*, INPA [image!], MO!, NY!) and appear similar to those of *V.excisa*; however, at maturity, its fruits tend to be minutely tomentelous to glabrescent (vs. densely tomentose; see Fig. 6E, I). Additionally, abaxial leaf blades of herbarium specimens of *V.calophylla* tend to be silver to golden.

*Virolaexcisa* can distinguished from *V.peruviana* by its staminate flowers with short anthers (0.5–0.7 mm vs. 1.1–1.6 mm long, from Smith and Wodehouse 1938) and fruits that are slightly carinate (vs. conspicuously carinate) and densely pubescent at maturity (vs. glabrescent). For a detailed comparison of the indument and carina of fruits between these two species see Figs 6I, K (fruits) and 14B, D (indument). Although, some specimens of *V.excisa* have a cordate leaf base (e.g. *V. Huashikat 2267*, MO; *D. Rubio & T. Coba 839*, MO), they are never as deeply cordate or as narrow as in *V.peruviana*, whose lobes also sometimes overlap or cover the twig (e.g. *W. H. Lewis et al. 10074*, MO).

#### Notes.

The majority of specimens cited here as *V.excisa*, including the source of illustrations in Pérez et al. (2014) and Jaramillo et al. (2004) were previously identified as *V.obovata*. In addition to the list of paratypes of *V.excisa* below, we provide a list of specimens that correspond to *V.obovata*. Our comparison between these two species is based on these specimens, as well as images of the original material of *V.obovata* collected and described by the Italian-Brazilian botanist, Adolpho Ducke (*A. Ducke 1509*, A, F, IAN, MG, NY!, RB-2 sheets, US-2 sheets).

#### Specimens examined.

**Colombia. Amazonas**: Araracuara, Río Caquetá, margen derecha 3 km arriba de la isla Sumaeta, 00°36'S, 072°10'W, 200–300 m, 31 May 1990 (fl bud), *E. Alvarez et al. 668* (COAH [n.v.], NY!); Parque Nacional Nataural Amacayacu, 03°47'S, 070°15'W, 200–220, 11 Nov 1991, *J. Pipoly et al. 15818* (COL!). **Putumayo**: selva higrófila del río Putumayo, Puerto Porvenir, arriba de Puerto Ospina, 230–250 m, 19 Nov 1940 (fr), *J. Cuatrecasas 10653* (COL!, F [image!]). **Ecuador. Napo**: Parque Nacional Yasuní, Carretera y Olecoducto de Maxus en construcción, Km 27, 00°35'S, 076°30'W, 250 m, 4–27 July 1993 (fl bud), *M. Aulestia 23* (MO!, UPCB [n.v]); Parque Nacional Yasuní, Pozo petrolero Daimi 2, 00°55'S, 076°11'W, 200 m, 26 May–8 Jun 1988 (♂ fl bud), *C. E. Cerón & F. Hurtado 3834* (INPA [image!], MEXU [image!], MO!, QCNE [n.v.], US [image!]); ibid., 26 May–8 Jun 1988 (♀ fl bud), *C. E. Cerón & F. Hurtado 4147* (COL!, INPA [image!], MO!, NY!); Parque Nacional Yasuní, Pozo Petrolero “Amo II” de Conoco, 00°52'S, 076°05'W, 230 m, 11–17 Jan 1988 (fr), *F. Coello 59* (MO!, NY!); La Joya de los Sachas, Comunidad de Pompeya, lado sur del Río Napo, Campamento de Maxus, Río Jivino, carretera Maxus Km 1–5, 00°25'S, 076°37'W, 220 m, 1–28 Sep 1992 (fr imm), *A. Grijalva & G. Grefa 115* (MO!, NY!); ibid., 23–29 Nov 1992 (fr), *A. Grijalva et al. 239* (MO!, QCA [n.v.]); La Joya de los Sachas, Pompeya, 00°25'S, 076°37'W, 250 m, 16–17 Aug 1992 (fr imm), *E. Gudiño 1677* (COL!, MO!, QCNE [n.v.]); Pompeya, Río Indillama, entre la desembocadura al Napo y el cruce de la carretera de MAXUS, 00°25'S, 076°37'W, 250 m, 13 Dec 1992 (st, fr), *E. Gudiño et al. 2139* (COL!, LOJA [n.v.], MO!, NY!, QCA [n.v.], UPCB [n.v.]); Pompeya, Carretera MAXUS km 3.9–5.2, 00°25'S, 076°37'W, 250 m, 14–15 Dec 1992 (fr), *E. Gudiño et al. 2154* (COL!, LOJA [n.v.], MEXU [image!], MO!, QCA [n.v.], QCNE [n.v.]); Estación Experimental INIAP-Payamino, costado oeste del Río Payamino, 5 km al NW de Coca, 00°13'S, 077°10'W, 300 m, 25 Sep 1985 (fr), *D. Neill et al. 6891* (INPA [image!], MO!, QCA [n.v.]); Maxus petroleum pipeline road, under construction, 2 km south of Río Napo, Comuna Pompeya, 00°30'S, 076°40'W, 220 m, 4 Dec 1992 (♀ fl, fr imm), *D. Neill et al. 10182* (MO!, NY!); 5 km al Norte de Coca y de la vía Coca-Payamino, Finca Tipán, 00°25'S, 077°00'W, 250 m, 22 Oct 1988 (fr), *W. Palacios 3186* (INPA [image!], MO!, PMA [image!], QCA [n.v.]); Tena, Estación Biológica Jatun Sacha, 10 km al oeste de la Estación, carretera hacia Tena, 01°03'S, 077°40'W, 500 m, 20 Sep 1989 (♀ fl bud), *W. Palacios & C. Iguago 4435* (INPA [image!], MO!); Añangu, Parque Nacional Yasuní, 00°31'32"S, 076°23'W, 260–350 m, 30 May–21 Jun 1982 (bud fl), *SEF no. 8934* (NY-2 sheets!); Estación Experimental INIAP-San Carlos, Reserva Florística EL Ahuano, 00°19'S 076°50'W, 250 m, 8 Sep 1986 (fr), *J. Zaruma 605* (NY!). **Pastaza**: Arajuno, Campamentos temporales 9, 22 y 25, línea propuesta del oleoducto Villano-CPF por ARCO, Km 25 noroeste del pozo Villano 2, 01°27'S, 077°36'W, 700 m, 3–14 Sep 1998 (♂ fl bud), *E. Freire & L. Santi 3380* (MO!); Pozo petrolero “Moretecocha” de ARCO, 75 km al este de Puyo, 01°34'S, 077°25'W, 580 m, 4–21 Oct 1990 (imm fr), *E. Gudiño et al. 956* (MO!, QCNE [n.v.]); Pozo petrolero Villano 2 de ARCO, 01°25'S, 077°20'W, 400 m, 1–18 Dec 1991 (fr), *F. Hurtado 2980* (MO!); Río Capihuari, tributary of Río Pastaza, 02°30'S, 076°50'W, 285 m, 23 July 1980 (bud fl), *B. Øllgaard et al. 35140* (NY!); Río Curaray, dos horas río abajo del pueblo Curaray, en la boca del Río Namoyacu, 01°24'S, 076°45'W, 275 m, 14–18 Aug 1985 (♂ fl bud), *W. Palacios & D. Neill 612* (INPA [image!], MO!, QCNE [n.v.]); Pozo Petrolero Villano 2, 01°29'S, 077°27'W, 24 July 1992 (♀ fl bud), *W. Palacios 10279* (MO!, QCNE [n.v.]); Pozo Petrolero “Danta 2” de UNOCAL, 50 km al sur-sureste de Curaray, 01°47'S, 076°48'W, 365 m, 1–19 Oct 1990 (fr), *D. Rubio & T. Coba 839* (INPA [image!], MEXU-2 sheets [image!], MO!); Ruta del oleoducto propuesto por ARCO, Villano-La Independencia, Km 24, 3 km al sur de San Virgilio, 01°24'S, 077°39'W, 830 m, 4 Sep 1997 (♂ fl), *H. Vargas et al. 1590* (MO!, QCNE [n.v.]). **Morona-Santiago**: Pozo petrolero “Garza” de TENNECO, 35 km (aprox.) al noreste de Montalvo, 01°49'S, 076°42'W, 260 m, 2–12 Jul 1989 (♂ fl bud), *V. Zak & S. Espinoza 4733* (INPA [image!], MO!, QCNE [n.v.]); [without province], margen izquierda del río San Miguel, entre Puerto Nuevo y Conejo, 28 Mar 1953 (fr), *G. Gutierréz 2716* (COL!). **Peru. Amazonas**: Valle del Río Santiago, aprox. 65 km N de Pinglo, quebrada Caterpiza, 2–3 km atrás de la comunidad de Caterpiza, [03°50'00"S, 077°40'00"W], 200 m, 19 Sep 1979 (fr), *V. Huashikat 682* (INPA [image!], MO!); ibid.,15 Mar 1980 (fr), *V. Huashikat 2267* (INPA [n.v.], MO!); ibid., 21 Mar 1980 (fr), *V. Huashikat 2322* (MO!); Condorcanqui, Distrito El Cenepa, Comunidad de Mamayaque, 04°34'49"S, 078°14'01"W, 400 m, 11 Aug 1997 (fr), *R. Rojas et al. 0272* (MO!); Bagua, Rainforest along Río Marañón 2–10 km above mouth of Río Santiago, 250–275 m, 14–15 Oct 1962 (fl), *J. J. Wurdack 2272* (NY!). **Huánuco**: Yuyapichis, Puerto Inca, 00°40'S, 075°02'W, 270 m, 01–15 Jul 1989 (bud fl), [*B.] Kröll 476* (NY!); ibid., 1–15 Jul 1989 (bud fl), [*B.] Kröll 487* (NY!); ibid., 1–15 Aug 1987 (♀ fl), [*B.] Kröll 527* (NY!); ibid., 16–31 Dec 1989 (fr), [without collector name] *Flores & Tello 192* (NY!); ibid., 16–30 Jun 1990 (fl bud), [without collector name] *Flores & Tello 1801* (NY!); ibid., 16–31 Aug 1990 (♀ fl), [without collector name] *Flores & Tello 1995* (NY!); ibid., 1–15 Aug 1987 (♂ fl), [without collector name] *Saito 14* (NY!). **Loreto**: Habanillo, Km 67, carretera Dtto. Iquitos-Nauta, 04°10'S, 073°30'W, 130 m, 03 Jun 1988 (fl bud), *R. Vásquez et al. 10712* (INPA [image!], MO!); Ucayali, vicinity of LSV base camp, Quebrada Shesha, tributary of Rio Abajao, ca, 65 km NE of Pucallpa, 08°02'S, 073°55'W, 250 m, 25 Jun 1987 (♂ fl), *A. [H.] Gentry & C. Díaz 58536* (MO!, NY!). **Pasco**: 6 km N of Puente Lorencillo No. 1 on Carretera Marginal, 32 km S of Dantas, 09°56'S, 075°00'W, 350 m, 30 Jun 1987 (st), *A. [H.] Gentry & C. Díaz 58640* (MO!). **Ucayali**: Río Blanco, [not elev.], 30 Jul 1923 (♂ fl), *G. Tessmann 3055* (NY!). **Brazil. Acre**: Cruzeiro do Sul, Rio Juruá, right margin of Igarapé Viseu, ca. 6 km from left bank of Rio Juruá, 08°15'S, 072°44'W, [not elev.], 05 Nov 1991 (fr), *C. A. Cid Ferreira et al. 10586* (NY!, UFACPZ [n.v.], UPCB [n.v.]); Jordão, along Rio Jordão, 09°13'28"S, 071°58'26"W, 230–250 m, 06 Feb 2009 (fr), *R. Goldenberg 1316* (NY!, RB [image!]); Porto Acre, Bacia do Rio Purus, Reserva Florestal de Humaitá, margem esquerda do Rio Acre, ca. 4 horas de barco abaixo de Rio Branco, 10°07'S, 069°13'W [not elev.], 03 Nov 1993 (fr), *M. Silveira et al. 718* (INPA [image!], NY, UFACPZ [n.v.]); Sena Madureira, trail from W bank of Rio Iaco to Rio Purus, 3 km above confluence, [not elev.], 5 Oct 1968 (fr), *G. T. Prance et al. 7862* (F [image!], INPA [image!], NY!, P [image!], U [image!], US [image!]); Tarauacá, 1–3 km east of Rio Tarauacá, at Tarauacá, [not elev.], 24 Sep 1968 (fr), *G. T. Prance 7510* (INPA [image!], NY!, US [image!]); Bacia do Rio Juruá, Rio Tarauacá, 08°27'39"S, 071°22'46"W, [not elev.], 19 Sep 1994 (imm fr), *M. Silveira et al. 840* (INPA [image!], NY!). **Amazonas**: Limoeiro, Est. Ecológica do Juamí Japurá, Rio Japurá margem direita abaixo da confluência com Rio Puruê, [not elev.], 16 Apr 1986 (fr), *C. A. Cid Ferreira et al. 7240* (NY!, UPCB [n.v.]); Parana do Autaz-Mirim, lago de Cobra, [not elev.], 25 Aug 1973 (♀ fl & imm fr], *C. C. Berg et al. P19742* (INPA [image!], NY!, US [image!]); Rio Solimões, loco Bom Futuro (ripa boreali fluvii, super São Paulo de Olivença), [not elev.], 4 Feb 1937 (♂ fl), [*W. A.] Ducke 396* (NY!, US [image!]); near mouth of Rio Embira (tributary of Rio Tarauaca), 07°30'S, 070°15'W, [not elev.], 06 Jun 1933 (♂ fl), *B. A. Krukoff 4713* (NY-2 sheets!, US [image!]); Lábrea, trail from W bank of Rio Purus, opposite Labrea, [not elev.], 30 Oct 1968 (fr), *G. T. Prance 8106* (NY!, US [image!]); Rio Purus, Lago Preto, 2 km north of Lábrea, [not elev.], 25 Jun 1971 (bud fl), *G. T. Prance et al. 13692* (NY!, US [image!]).

#### Specimens examined of *Virolaobovata*.

**Colombia. Amazonas**: Puerto Nariño, [not elev.], 24 Jul 1965 (♂ fl), *G. Lozano C. et al. 588* (COL!); trapecio Amazónico, entre los ríos Loretoyacu y Hamacayacu, [not elev.], [s.d.] Dec 1945 (fr), *J. M. Duque-Jaramillo 2366* (COL); Araracuara, Pintadillo (frente a la tercer isla), margen izquierda río Caquetá, [not elev.], 14 Apr 1986 (fr), *J. H. Torres et al. 3159* (COL). **Peru. Loreto**: Maynas, Rio Yuvineto, affluent du Putumayo, [not elev.], 06 Feb 1978 (fr), *S. Barrier 549* (NY!); Maynas, near Villa Nueva, Borro Indian village on upper Río Yaguasyacu, tributary of Río Ampiyacu, [not elev.], 8 Nov 1977 (fr), *A. [H.] Gentry & J. Revilla 20448* (MO!); Maynas, in vicinity of Mishana, between Río Nanay and Río Itaya, 130 m, 29 Nov 1977 (fr), *A. [H.] Gentry et al. 21006* (MO); Maynas, Mishana, Río Nanay, halfway between Iquitos and Santa María de Nanay, 130 m, [s.d.] 1979 (♂ fl), *R. Ramírez 10* (MO!); Iquitos, Allpahuayo, bosque del Instituto de Investigación de la Amazonía Peruana, km 21 carretera Quistococha-Nauta, [03°58'16"S, 073°25'07"W], [not elev.], 5 Jul 1992 (♂ fl), *R. Rueda & J. Ruíz 621* (MO!); Maynas, Llachapa, (Explor Napo), Río Napo, 130 m, 21 Jan 1983 (fr), *R. Vásquez & N. Jaramillo 3822* (MEXU [image!], MO!, NY!); Iquitos, Allpahuayo, Estación Experimental del Instituto de Investigaciones de la Amazonía Peruana (IIAP), [03°58'16"S, 073°25'07"W], [not elev.], 22 Aug 1990 (imm fr), *R. Vásquez et al. 14250* (MO!).

### ﻿8. Virola parkeri

Taxon classificationPlantaeMagnolialesMyristicaceae

D. Santam. & Lagom. sp. nov.

61765F25-F214-5BB4-A7F8-61010E9962B3

urn:lsid:ipni.org:names:77298667-1

#### Type.

**Figure 15.**
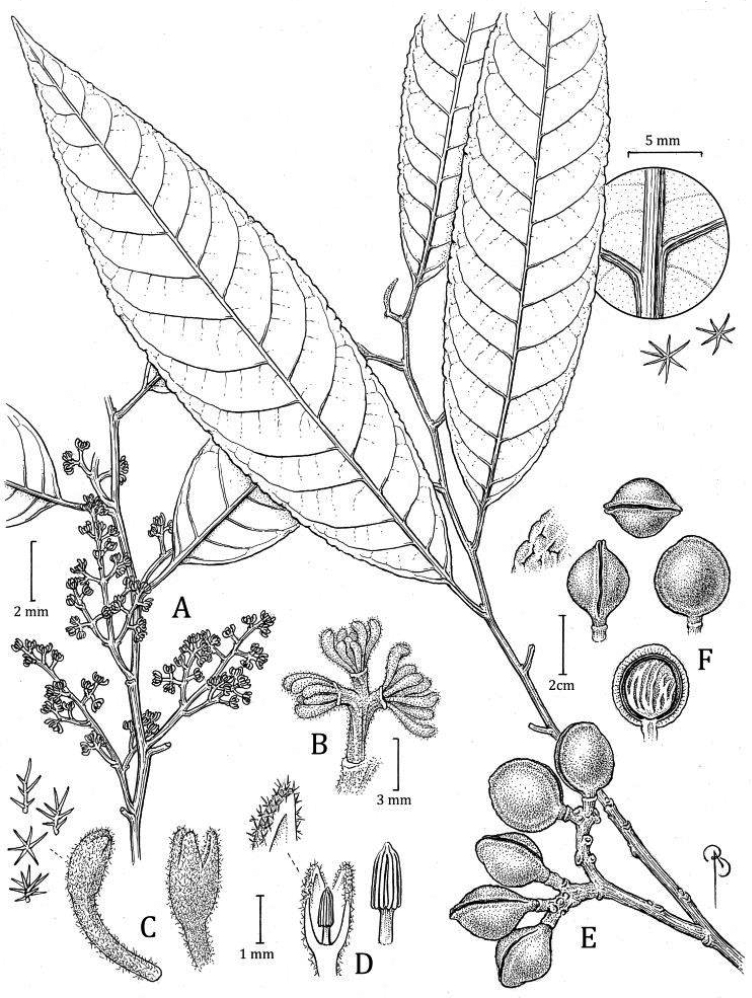
*Virolaparkeri***A** branch with staminate inflorescence **B** part of staminate inflorescence **C** lateral view of staminate perianth with an enlargement of trichomes (left) **D** medial sections of staminate flower, with detail of the lobe with trichomes (above left), and androecium (right) **E** branch with infructescence and detail of abaxial surface of leaf blade showing the midvein with enlargement of trichomes (top right) **F** different views of the fruits, detail of fruit surface (left), and an open fruit showing the seed covered by a laciniate aril (below). Drawn by Bobbi Angell based on *M. Huamán et al. 334* (**A–D** MO), and *A. Monteagudo et al. 10761* (**E–F** MO).

**Peru. Pasco**: Oxapampa, Dist. Palcazú, Evaluación de los Recursos del Bosque 0.5 ha en la Reserva Comunal Yanesha, Comunidad Nativa Loma Linda-Laguna, Sector Nueva Aldea, 10°23'13"S, 075°05'28"W, 600–620 m, 17 Oct 2005 (fr), *A. Monteagudo, A. Peña, R. Francis, L. Quicha, E. Camavilca & W. G. Camaña 10761* (holotype: MO! [accession 6101576, barcode MO-2134612], isotypes: AMAZ [n.v.], HUT [n.v.], MOL [n.v.], USM [n.v.], US [accession 3558469; image!]). Fig. 15

#### Diagnosis.

*Virolaparkeri* morphologically differs from all other species by the combinations of the fruit with a conspicuous “wing” along its dehiscence line, the surface is bullate and inconspicuously pubescent, flowers with the column of filaments shorter (0.2–0.4 mm long) than the anthers (0.5–0.7 mm long), leaf blades covered abaxially with inconspicuous, stellate and sessile trichomes and lateral veins that are evenly spaced.

***Tree*** 28–35 m tall, diameter, inner bark not described. ***Exudate*** reddish, location of exudate on plant not stated. ***Twigs*** 0.2–0.7 cm thick, terete, puberulent to glabrescent, trichomes stellate, sessile, brown-reddish, without lenticels. ***Leaves*** young terminal bud ca. 1.1 × 0.2 cm; petiole 1.4–1.7 × 0.16–0.22 cm, slightly canaliculate, puberulent (especially within the channel) to glabrescent, the trichomes stellate, sometimes with hyaline squamiform structures between the trichomes; leaf blades 19.7–24 × 5.2–6.4 cm, oblong; adaxial surface of mature leaves drying brown to grayish, glabrous, the surface smooth, sometimes shiny; abaxial surface drying white-grayish to pale brown, sparsely pubescent, the trichomes stellate, ca. 0.1 mm diameter, sessile, the central part of the trichome usually colorless, but sometimes with the central part reddish, the branches colorless; lateral veins 15–17 per side, 4 veins per 5 cm, spaced 1.5–1.7 (–2) cm, on adaxial side flat, the same color as the adaxial surface or a little darker, on abaxial surface slightly raised, tomentose, arcuate-ascending, slightly anastomosing near the margin and without forming a marked intramarginal vein; tertiary veins very slightly visible on both sides; midvein adaxially slightly elevated, immerse in a small channel, abaxially raised, rounded, puberulent to glabrescent, usually with denser pubescence on the sides; base acute, not revolute, flat; margin flat; apex acute. ***Staminate inflorescence*** 5.8–7 cm long, axes flattened, tomentose, with dendritic trichomes, ferruginous; peduncle 1.5–1.8 × 0.18–0.24 cm; main axes with 9–10 ramifications, the first pair opposite, subopposite or alternate, the others alternate; bracts not seen. ***Staminate flowers*** (in bud) in dense terminal fascicles of 10–30+ flowers, on a receptacle 1.5–2.5 mm wide; perianth 1.5–1.8 mm long, ovate to lanceolate, creamy yellow when fresh, connate to ca. 1 mm in length, external surface densely pubescent with ferruginous and dendritic trichomes, internal surface moderately pubescent (especially on the lobes); lobes 3, ca. 0.9 × 0.5 mm, ca. 0.2 mm thick, without resinous punctuations when rehydrated; stamens 3, the filament column 0.2–0.4 mm long and ca. 0.2–0.3 mm thick, glabrous, straight, constricted to slightly constricted at the apex; anthers 0.5–0.7 mm long, ca. 0.2 mm wide; apiculus ca. 0.8 mm long, acuminate, connate. ***Pistillate inflorescences*** and ***pistillate flowers*** not seen. ***Infructescence*** ca. 2.5 cm long, with 1–5 fruits, peduncle ca. 1 × 0.6 cm. ***Fruits*** 3–3.6 × 2.8–3.1 cm, green when fresh (brown to blackish when dry), oblate, shortly stipitate, very inconspicuously pubescent (not falling like dust), puberulent at the base and sometimes on the carina, the trichomes stellate (sometimes with few dendritic), sessile, ferruginous, the surface bullate, the line of dehiscence winged, the wing ca. 0.4 cm long, the base obtuse, the apex rounded; pericarp ca. 0.3 mm thick (on the thickest side; ca. 0.2 mm thick on the thinnest side); pedicel 0.4–0.6 cm long. ***Seed*** ca. 1.9 × 1.6 cm, the testa brown to blackish when dry, smooth; aril color when fresh not described, blackish when dry, the texture dry and thin, laciniate almost to the base, in narrow bands distally.

#### Distinctive characters.

The most striking character of *Virolaparkeri* is its fruit, which is reminiscent of Saturn’s form: it is oblate with a conspicuous “wing” along its dehiscence line (Fig. 6L, 15F). The fruit is further bullate and inconspicuously pubescent. Other characteristics that distinguish this new species are its leaf blades that are covered abaxially with inconspicuous, usually colorless stellate and sessile trichomes and lateral veins that are evenly spaced; staminate flowers with perianth that is densely pubescent outside and the inner side moderately pubescent, the lobes are relatively thin, the column of filaments are shorter (0.2–0.4 mm long) than the anthers (0.5–0.7 mm long); and the thin, laciniate aril that covers the seed almost to the base.

#### Etymology.

The specific epithet honors Theodore A. Parker III (1 Apr. 1953–3 Aug. 1993), renowned and talented ornithologist and research associate of the LSU Museum of Natural Science. Parker died in a plane crash on August 3, 1993 while surveying a remote forest in Ecuador, along with three other people: Raul Mortensen (the pilot), Eduardo Aspiazu (ecologist), and Alwyn H. Gentry (botanist). *Virolaparkeri* occurs in Peru, a country where Parker spent much of his life studying birds and protecting natural resources. In this country, Parker together with Scott Robinson, established a Big Day record (331 bird species). He also contributed to a field guide to the birds of Peru, an important resource for birdwatching ecotourism. Finally, Parker was part of the Rapid Assessment Program (RAP) at Conservation International, which involved teams of scientists conducting biological surveys in remote areas of the tropics to determine their level of biodiversity and potential for conservation. Thanks to Parker’s efforts, Bolivia established Madidi National Park, one of the most important centers for biodiversity.

#### Distribution.

*Virolaparkeri* is known only from Peru (Pasco Department) (Fig. 18C). It is found in primary forest between 400 and 620 m in elevation.

#### Phenology.

Staminate flowers and fruits of *Virolaparkeri* have been recorded in October. Pistillate flowers were not seen in the studied material.

#### Common name and uses.

Common names include banderín and rrohuatquech (Peru; *M. Huamán et al. 334*). The wood is used in the manufacture of furniture and boxes, and house building (*M. Huamán et al. 334*).

#### Preliminary conservation status.

*Virolaparkeri* is Critically Endangered following IUCN criterion B2a. Justifying this status, it is known from only a single specimen, collected in 2008. The only locality of this species is within the Loma Linda community of the Yanesha indigenous community, and so may benefit from an ongoing compensated community-based forest monitoring program (Kowler et al. 2020).

#### Discussion.

*Virolaparkeri* is morphologically similar to *V.yasuniana* from Ecuador (which is formally described below) in various leaf traits, including overall shape, trichomes on the abaxial surface, lateral vein separation, and color when dry; additionally, both species share staminate perianth that is moderately pubescent internally and fruits with a markedly carinate dehiscence line, appearing like a wing. Differences among the two species are summarized in Table 10.

**Table 10.** Comparison of *Virolaparkeri*, with *V.yasuniana*.

Morphological character	*V.parkeri*	*V.yasuniana*
Leaf blade size	19.7–24 × 5.2–6.4 cm	15.7–27.5 × 4.4–6.6 cm
Spaced lateral veins	1.5–1.7 (–2) cm apart	0.9–1.5 (–1.9) cm apart
Staminate perianth lobes	ca. 0.9 × 0.5 mm	0.7–1 × 0.5–0.8 mm
Filament column	0.2–0.4 mm long, and ca. 0.1 mm wide at the base	0.3–0.5 mm long, and ca. 0.2–0.3 mm wide at the base
Anthers long	0.5–0.7 mm	0.5–0.6 mm
Apiculus long	ca. 0.8 mm	ca. 0.1 mm
Fruit size	3–3.6 × 2.8–3.1 cm	3.4–4.2 × 3.1–4 cm
Fruit carina	ca. 0.4 cm long (Fig. 6L)	0.4–0.7 cm long (Fig. 6M)
Seed	ca. 1.9 × 1.6 cm	ca. 2.5 × 1.9 cm

*Virolaparkeri*’s fruit with a wing-like carina resembles those of *V.peruviana* (see Fig. 6K, L). However, the new species is distinguished from *V.peruviana* in its leaves with acute bases (vs. usually cordate), fewer lateral veins (15–17 vs. 17–30 per side) that are usually more separated (1.5–1.7 [–2] vs. 0.6–1.3 [–1.7] cm), staminate inflorescences that are narrower and shorter, staminate flowers with the filament column shorter than anthers (0.5–0.7 mm vs. 1.1–1.6 mm long; from Smith and Wodehouse 1938), and oblate (vs. ellipsoid) fruits with rounded apex (vs. acute or apiculate).

#### Notes.

The type specimen of *Virolaparkeri* (*A. Monteagudo et al. 10761*) was previously identified as *Otobaparvifolia* (Markgr.) A. H. Gentry. However, while species of *Otoba* have malpighiaceous trichomes on abaxial leaf surfaces of leaf blades, these trichomes are dendritic or stellate in *Virola*. The other specimen studied (*M. Huamán et al. 334*) was identified as *V.elongata*. Duplicates may have been distributed under these names.

#### Specimens examined.

**Peru. Pasco**: Oxapampa, Palcazú, Comunidad Nativa Loma Linda-Laguna, sector Nueva Aldea, Bosque de la Asociación Forestal Yanesha Concoll-Toroñ (AFYCT), 10°21'51"S, 075°03'20"W, 400 m, 16 Oct 2008 (♂ fl), *M. Huamán et al. 334* (MO!, P [image!]).

### ﻿9. Virola tuckerae

Taxon classificationPlantaeMagnolialesMyristicaceae

D. Santam. & Lagom. sp. nov.

8B81F7EB-C487-55F8-9FE6-80F03D873554

urn:lsid:ipni.org:names:77298668-1

#### Type.

**Figure 16.**
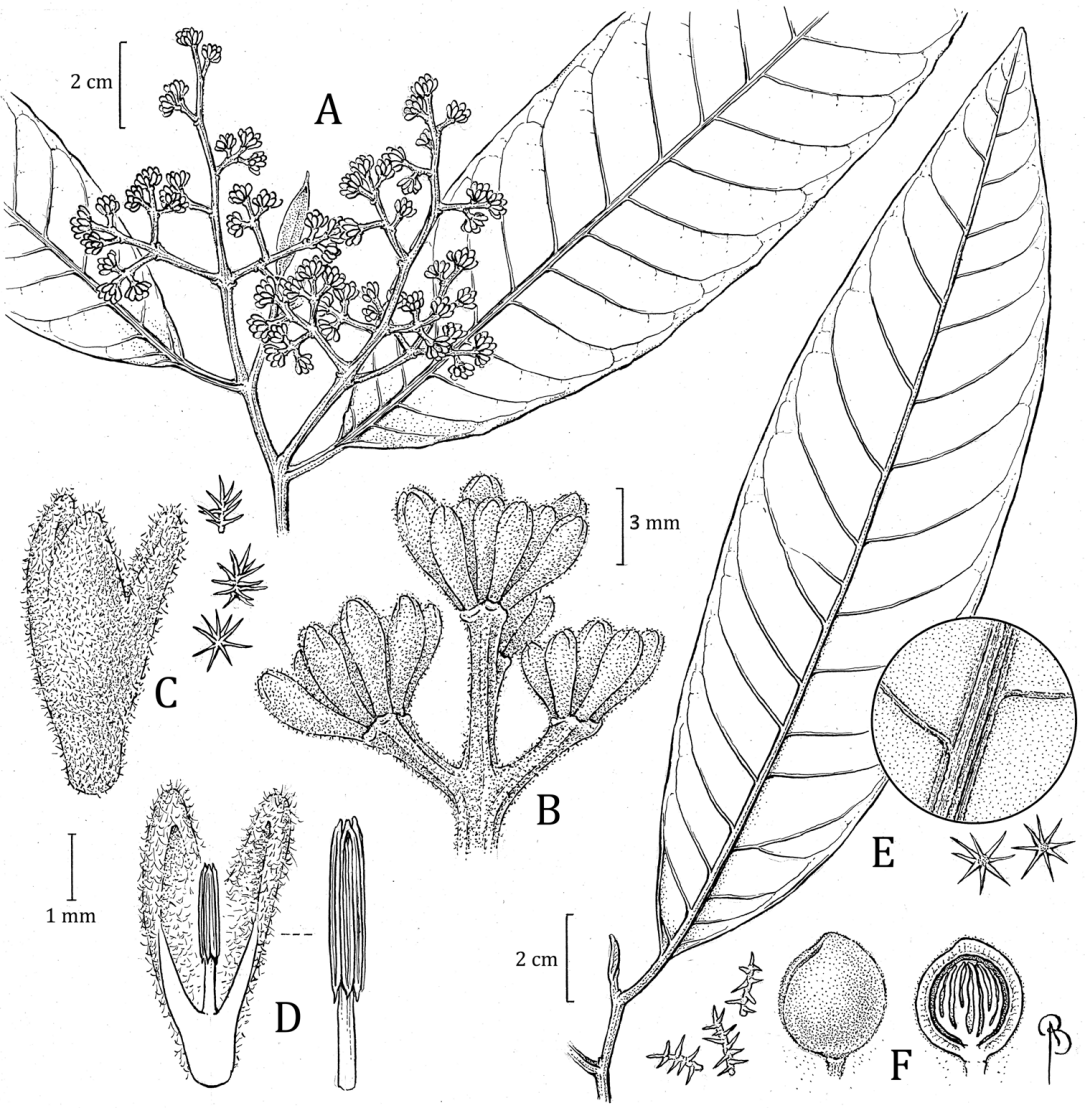
*Virolatuckerae***A** branch with staminate inflorescence **B** partial staminate inflorescence **C** lateral view of staminate perianth with an enlargement of trichomes (right) **D** medial section of staminate flower and androecium (right) **E** Leaf blade on adaxial side, with detail of petiole and trichomes **F** fruits with detail of trichomes (left) and an open fruit showing seed covered with laciniate aril (right). Drawn by Bobbi Angell based on *J. Pipoly et al. 16797* (**A–D** MO), and *Á. Cogollo et al. 4147* (**E–F** MO).

**Colombia. Antioquia**: Las Orquídeas, Vereda Calles, Parque Nacional Natural Las Orquídeas, Quebrada Honda, filo al NW de La Cabaña Calles, Parcela W, subparcelas W 8-W 9, 1300 m, 06°29'N, 076°14'W, 11 Dec 1992 (♂ fl), *J. Pipoly, Á. Cogollo, D. Cádenas, M. Villa, O. Alvarez, L. Velez 16962* (holotype: MO! [accession 05011143, barcode MO-2657528], isotypes: not seen). Fig. 16

#### Diagnosis.

*Virolatuckerae* is similar to *V.cogolloi* in share similar distributions, leaf blades that are densely pubescent abaxially. Morphologically, it differs in having staminate flowers with long filament column (1.2–1.4 mm long vs. 1.1–1.2 mm), fruits with an inconspicuous layer and persistent trichomes (conspicuous layer of trichomes, caducous, that fall like dust). *Virolatuckerae* previously confused with *V.sebifera*. It differs from these by the staminate flowers with perianth internally densely pubescent (vs. glabrous or almost glabrous), long staminal column (0.6–0.7 [–0.9] mm long vs. 0.2–0.6 mm), and large fruits (2.7 × 2.5 cm vs. 1–1.9 [–2.1]).

***Tree*** (12–) 18–30 m × 17–30.1 cm diameter, inner bark not described. ***Exudate*** red, location of exudate on plant not stated, or in the fruit hyaline and oxidizing red. ***Twigs*** 0.21–0.37 cm thick, terete or slightly compressed, tomentose, trichomes dendritic, sessile, ferruginous, without lenticels or lenticels very small and scattered. ***Leaves*** young terminal bud 1.2–2 × 0.24–0.31 cm; petiole 1.1–1.7 (–2) × 0.23–0.35 (–0.5) cm, slightly canaliculate, not winged or slightly winged (*J. Pipoly et al. 16805*), tomentose, the trichomes dendritic; leaf blades 21–29.5 (–33.5) × 5–7.2 (–11.7) cm, narrowly oblong or rarely elliptical; adaxial surface when drying on mature leaves brown to blackish brown, the surface smooth, sometimes shiny, glabrous; abaxial surface when drying pale brown to grayish brown, densely pubescent, the trichomes stellate, ca. 0.1 mm diameter, sessile, the central part of the trichome pale reddish, sometimes a little darker, the branches pale reddish and not contrasting much in color with the central part of the trichome; lateral veins 16–19 per side, 3–4 (–6) veins per 5 cm, spaced 1.1–1.8 (–2.1) cm, on adaxial side, the same color as the adaxial surface or slightly darker, flat to slightly raised, on abaxial surface blackish to brown reddish, raised, puberulent to glabrescent above, densely pubescent to the sides, arcuate-ascending distally, slightly anastomosing near the margin and without forming a marked intramarginal vein; tertiary veins very slightly visible on both sides, but especially above; midvein adaxially flat to slightly elevated, abaxially raised, rounded, tomentose to puberulent, more pubescent to the sides; base cuneate, not revolute, flat; margin flat; apex acute. ***Staminate inflorescence*** 5–9.5 cm long, axes flattened, tomentose, trichomes dendritic, ferruginous; peduncle 0.6–1.8 (–2.8) × 0.2–0.38 cm; main axes with (3–) 5–9 (–12) ramifications, the first pair opposite to subopposite, the others alternate; bracts not seen. ***Staminate flowers*** (in bud) in dense terminal fascicles of 9–20 flowers, on a receptacle 2–3 mm wide; perianth 3.5–5 mm long, oblong, fleshy, ferruginous when fresh (probably by the trichomes), connate to 1.5–2.5 mm in length, external surface densely pubescent with ferruginous and dendritic trichomes, internal surface densely pubescent (especially in the lobes); lobes 3, 2–2.5 × 1.2–1.7 mm, and 0.2–0.5 mm thick, without resinous punctuations when rehydrate); stamens 3, the filament column 0.6–0.7 (–0.9) mm long and 0.2 mm wide, glabrous, straight or sometimes a little wider at the base, not constricted at the apex; anthers 1.2–1.6 mm long, and 0.2–0.4 mm wide; apiculus 0.1–0.2 mm long, acuminate, slightly separated or connate. ***Pistillate inflorescence*** and ***flowers*** unknown. ***Infructescence*** unknown. ***Fruit*** 2.7 × 2.5 cm (only one seen, and that immature; *Á. Cogollo et al. 4147*), when fresh green and covered with brown trichomes, globose, densely tomentose, the trichomes dendritic (ca. 0.1–0.2 mm long), sessile, ferruginous, that fall as easily as dust, the surface probably smooth, the line of dehiscence slightly carinate, the base and the apex obtuse; pericarp ca. 2.4 mm thick; pedicel unknown. ***Seed*** length unknown × ca. 1.4 cm, the testa brown when dry, slightly ribbed distally; aril color not described when fresh, blackish when dry, the texture dry and thin, laciniate almost to the base, in narrow bands distally.

#### Distinctive characters.

*Virolatuckerae* can be recognized by its narrow, oblong leaves with relatively close lateral veins (3–4 [–6] veins per 5 cm) and a cuneate base; its short-pedunculate staminate inflorescence with flowers organized in dense fascicles; its staminate flowers with fleshy perianth that is densely pubescent on both sides and a straight filament column that is shorter (0.6–0.7 [–0.9]) mm long) than the anthers (1.2–1.6 mm long); and its globe fruit that is densely tomentose with dendritic and ferruginous trichomes that fall like dust (Fig. 6N). Like other species described here, the new species is covered with stellate and sessile trichomes on the abaxial side of the leaf blades.

#### Etymology.

It is a pleasure to name a species of *Virola* in honor of Dr. Shirley Cotter Tucker, a botanist, lichenologist, and Professor Emeritus at Louisiana State University (LSU). Despite many challenges she faced as a woman in science, Shirley has had an illustrious career marked by many honors, including a Boyd Professorship, the most prestigious academic rank granted at LSU to internationally renowned scholars. Shirley is an important leader in botany, and served as president of two of the USA’s most prominent botanical societies, the Botanical Society of America and the American Society of Plant Taxonomists. Shirley’s intellectual contributions to botany are lasting, providing the foundation framework from which current research on floral morphology and evolution builds. Much of Shirley’s academic research (which includes more than 100 published articles) has focused on floral morphology and anatomy, especially of legumes and magnoliids— including studies within Myristicaceae (Armstrong and Tucker 1986), making it particularly special to name a species of *Virola* in her honor. She maintains her passion for lichens into retirement, and actively curates loans of lichen specimens from her home in Santa Barbara, California. The generous philanthropy of Shirley and her late husband, Kenneth Tucker, have greatly benefitted the botanical community. At LSU, where two of the authors work, donations from the Tucker family established the Shirley C. Tucker Endowed Chair in Plant Systematics. The LSU herbarium is named in her honor.

#### Distribution.

*Virolatuckerae* is only know from Antioquia, Colombia (Fig. 18A). It has been collected in premontane wet forest at 1300–1420 m elevation.

#### Phenology.

*Virolatuckerae* was collected with flowers in December and fruit specimens collected in February. Pistillate flowers were not seen in the studied material.

#### Common name and uses.

Sebo cordillero (Colombia; *Á. Cogollo et al. 4147*).

#### Preliminary conservation status.

*Virolatuckerae* is Endangered following IUCN criterion B2a. Justifying this status, it is known from two localities and has an AOO of 4 km^2^. While *V.tuckerae* benefits from its occurrence within Las Orquídeas National Park, this region (including within the national park) is still vulnerable to deforestation to expand human activities, including agriculture and livestock grazing (Pedraza-Peñalosa 2015; González-Caro and Vásquez 2017).

#### Discussion.

All the studied specimens with flowers of *Virolatuckerae* were previously identified as *V.sebifera*, a species that is widely distributed from Central to South America. While *V.sebifera* has variable leaf morphology (i.e., shape, base, apex), it differs from *V.tuckerae* in its long staminate inflorescences (8–23 cm long vs. 5–9.5 cm long), internally glabrous or almost glabrous perianth (vs. densely pubescent), shorter staminal column (0.2–0.6 mm vs. 0.6–0.7 [–0.9] mm long), and smaller fruits (1–1.9 [–2.1] × 0.7–1.4 [–1.7] cm vs. 2.7 × 2.5 cm) that are usually covered by the dense and thick layer of trichomes. While the type of abaxial leaf pubescence is variable in *V.sebifera*, dendritic, pediculate trichomes are common (vs. stellate, sessile trichomes). Further, *V.sebifera* usually occurs at lower elevations.

*Virolatuckerae* shares similarities with *V.yasuniana*, including its leaf shape, sessile, stellate trichomes on the abaxial leaf surface, and the color of dried herbarium specimens. *Virolayasuniana* is a species primarily from the lowlands of Ecuador (200–480 [1000] m elevations) that is formally described below. However, *V.tuckerae* differs from it in its densely pubescent abaxial leaf surface (vs. sparsely pubescent to glabrescent in *V.yasuniana*) (Fig. 4N, O), staminate flowers with perianth that is densely pubescent internally (vs. moderately pubescent) and long anthers (1.2–1.6 mm vs. 0.5–0.6 mm long), and densely pubescent fruits (vs. puberulent).

Finally, *Virolatuckerae* and *V.cogolloi*, grow closely in the same region (Urrao, sector Calles, Colombia); the differences and similarities between these species are discussed under *V.cogolloi*.

#### Notes.

As mentioned above, collections with flower have been previously identified as *V.sebifera*. The specimen with fruit (*Á. Cogollo et al. 4147*) was previously identified as *V.elongata*. Duplicates may have been distributed under these names.

#### Specimens examined.

**Colombia. Antioquia**: Parque Nacional Natural Las Orquídeas, Margen derecha del Río Calles y de la qubrada “El Guaguo”, 06°32'N, 076°19'W, 1390–1420 m, 12 Feb 1989 (fr), *Á. Cogollo et al. 3924* (COL!); Urrao, Parque Nacional Natural Las Orquídeas, Margen derecha del Río Calles, 06°32'N, 076°19'W, 1300 m, 21 Feb 1989 (fr), *Á. Cogollo et al. 4147* (COL!, MO!); Urrao, Parque Nacional Natural Las Orquídeas, Vereda Calles Quebrada Honda, filo NW de La Cabaña Calles, 06°29'N, 076°14'W, 1330 m, 8 Dec 1992 (♂ fl), *J. Pipoly et al. 16797* (MO!, NY!); ibid., 8 Dec 1992 (♂ fl), *J. Pipoly et al. 16805* (MO!, NY!); Las Orquídeas, Vereda Calles, filo NW de La Cabaña Calles, 06°29'N, 076°14'W, 1330 m, 10 Dec 1992 (♂ fl), *J. Pipoly et al. 16881* (MO!, NY!); ibid., 10 Dec 1992 (♂ fl), *J. Pipoly et al. 16888* (MO!).

### ﻿10. Virola yasuniana

Taxon classificationPlantaeMagnolialesMyristicaceae

D. Santam. sp. nov.

54D6138D-14B6-5DAF-ABC8-22AC298219B9

urn:lsid:ipni.org:names:77298669-1

#### Type.

**Figure 17.**
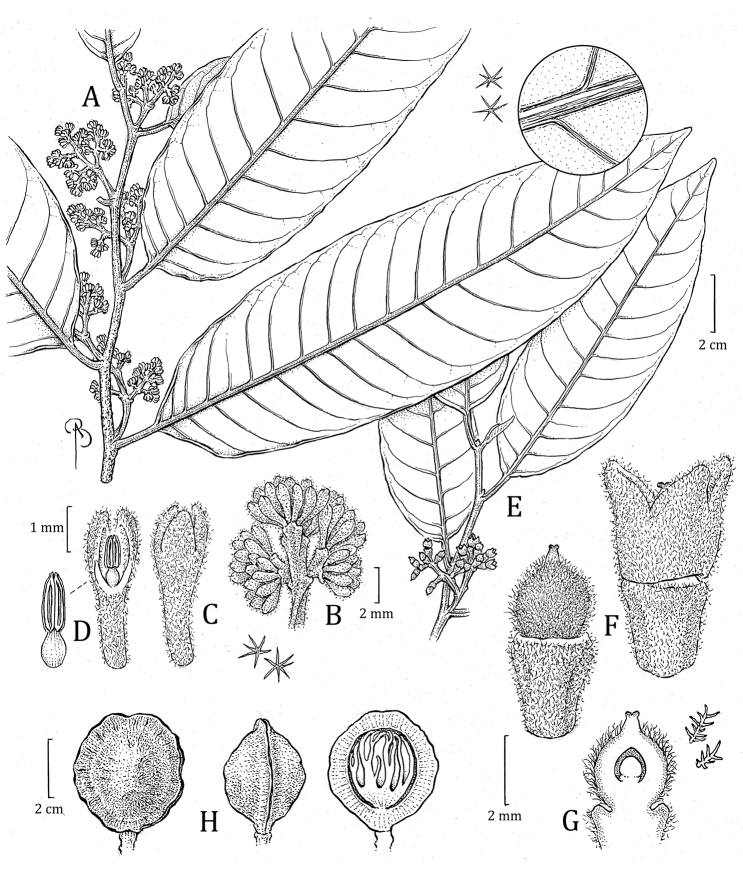
*Virolayasuniana***A** branch with staminate inflorescence, with detail of midvein and trichomes (upper right) **B** partial staminate inflorescence **C** lateral view of staminate perianth **D** medial section of staminate flower (right) and androecium (left) **E** branch with pistillate inflorescence **F** lateral view perianth (right) of pistillate flower and gynoecium (left) **G** Medial section of pistillate flower, showing detail of trichomes on gynoecium (right) **H** different views of the fruits, and an open fruit (right) showing the seed covered by a laciniate aril. Drawn by Bobbi Angell based on *V. Zak & S. Espinoza 5039* (**A–D** MO), *C. Cerón & F. Hurtado 6560* (**E–G** MO); *V. Zak & S. Espinoza 5149* (**H** MO).

**Ecuador. Pastaza**: Pastaza Cantón, Pozo petrolero “Ramírez”, 20 km al sur de la población de Curaray, 300 m, 01°32'S, 076°51'W, 21–28 Feb 1990 (fr), *V. Zak & S. Espinoza 5149* (holotype: MO! [accession 04782630, barcode MO-713299], isotypes: NY!, QCNE [n.v.], US [accession 3625319; image!). Fig. 17

#### Diagnosis.

*Virolayasuniana* morphologically differ from all others species by the combinations of large fruits (3.4–4.2 × 3.1–4 cm) with very conspicuous wings in the line of dehiscence, staminate flowers, with a filaments column that is wide at the base, constricted at the apex, and usually shorter (0.3–0.5 mm long) than the anthers (0.5–0.6 mm long).

***Tree*** 15–30 m × 30–40 cm diameter, outer bark brown, powdery, rough and thin, inner bark orange-red. ***Exudate*** translucent on internal bark, or watery reddish or reddish purple, location of exudate on plant not stated. ***Twigs*** 0.2–0.4 cm thick, terete, puberulent to glabrescent, trichomes stellate, sessile, brown-reddish to whitish, sometimes slightly lenticellate. ***Leaves*** young terminal bud 1–2 × 0.19–0.28 cm; petiole 1.1–1.7 × 0.15–0.21 cm, canaliculate, sometimes very short alate, puberulent, sometimes tomentose, the trichomes stellate; leaf blades 15.7–27.5 × 4.4–6.6 cm, lanceolate to oblong; adaxial surface when drying on mature leaves pale to dark brown, grayish, or blackish, the surface smooth, sometimes shiny, glabrous; abaxial surface when drying grayish, pale to dark brown, or white-grayish, sparsely pubescent to glabrescent, the trichomes stellate, ca. 0.1 mm diameter, sessile, the central part of the trichome colorless or reddish, the branches brown-reddish or colorless; lateral veins 14–18 per side, 4–5 veins per 5 cm, spaced 0.9–1.5 (–1.9) cm, on adaxial side, the same color as the adaxial surface or a little darker, flat, on abaxial surface slightly raised, glabrous to scattered pubescent, arcuate-ascending, slightly anastomosing near the margin and without forming a very marked intramarginal vein; tertiary veins very slightly visible on both sides; midvein adaxially slightly elevated, abaxially raised, rounded to triangular, puberulent to glabrescent; base acute, not revolute, flat; margin flat; apex acute. ***Staminate inflorescence*** 6–6.7 cm long, axes flattened, tomentose, trichomes appressed dendritic, ferruginous; peduncle 1.2–1.7 × 0.19–0.26 cm; main axes with 6–9 ramifications, the first pair opposite to subopposite, the others alternate; bracts not seen. ***Staminate flowers*** (in bud) in dense terminal fascicles of 15–25+ flowers, on a receptacle 2.1–3 mm wide; perianth 1.3–1.8 mm long, subglobose to infundibuliform to ovate, subcarnose, brown when fresh, connate to 1.2–1.5 mm in length, external surface densely pubescent with ferruginous and dendritic-stellate trichomes, internal surface moderately pubescent (especially in the lobes); lobes 3, 0.7–1 × 0.5–0.8 mm, ca. 0.1 mm thick, without resinous punctuations when rehydrated; stamens 3, the filament column 0.3–0.5 mm long, ca. 0.2–0.3 mm wide, glabrous, wide at the base and constricted at the apex; anthers 0.5–0.6 mm long, and 0.3–0.4 mm wide; apiculus ca. 0.1 mm long, acuminate, connate. ***Pistillate inflorescence*** 2.6–.2.9 cm long, axes flattened, tomentose, with trichomes dendritic, ferruginous; peduncle ca. 1 × 0.2–0.3 cm; bracts not seen. ***Pistillate flowers*** in terminal fascicles of 1–2 flowers, on a receptacle 2.5–3.5 mm wide; perianth ca. 3–3.1 mm long, ovate, subcarnose, brown when fresh, connate by ca. 1.5–2 mm long, external surface densely pubescent with ferruginous and dendritic trichomes, internal surface moderately pubescent (especially in the lobes), sometimes slightly pubescent at the base; lobes 3, ca. 1.3–1.8 × 1.1–1.3 mm, and ca. 0.1 mm thick; gynoecium ca. 2–3 × 1.6–2.4 mm, globose, densely pubescent, with ferruginous trichomes; stigma 2-lobed, ca. 0.3–0.5 × 0.3 mm, erect, flat seen from above, drying blackish, slightly wavy at the margins. ***Infructescence*** 2.3–3.4 cm long, with 1–2 fruits, peduncle 0.7–1.5 × 0.34–0.6 cm. ***Fruits*** 3.4–4.2 × 3.1–4 cm (including wings), green when fresh (blackish when dry), ellipsoid to somewhat flattened (immature), shortly stipitate, puberulent, the trichomes stellate, sessile, ferruginous or whitish and not falling like dust, the surface rugulose, the line of dehiscence winged, the wing 0.4–0.7 cm long, the base rounded to subcordate, the apex rounded; pericarp 1.3 and 2.3 mm thick (measured from two specimens); pedicel 0.7–1 cm long. ***Seed*** ca. 2.5 × 1.9 cm, the testa drying dark brown to yellowish, slightly ribbed distally; aril color when fresh described once as red, brown-reddish to blackish when dry, the texture dry and thin, laciniate almost to the base, in narrow bands distally.

#### Distinctive characters.

The very distinctive fruits of *Virolayasuniana*, which are large with very conspicuous wings in the line of dehiscence (Fig. 6M), make it almost impossible to confuse with any other species of *Virola* already described. Other characteristics that distinguish this new species include leaf blades that are abaxially covered with inconspicuous stellate and sessile trichomes, evenly spaced lateral veins that are comparatively spaced, and moderately pubescent internal perianth in staminate flowers, and a filaments column that is wide at the base, constricted at the apex, and usually shorter (0.3–0.5 mm long) than the anthers (0.5–0.6 mm long).

#### Etymology.

The specific epithet refers to Parque Nacional Yasuní, where most of the collections of this new species come from. At present, 2700 vascular plant species are documented from Yasuní, including a high diversity of lianas, epiphytes, and ferns; it is estimated its flora comprises approximately 3213 species; Yasuní is also home to *ca.* 1570 species of birds, fishes, mammals, amphibians and reptiles (Bass et al. 2010; Pérez et al. 2014). The description of *Virolayasuniana* is one step closer to a full documentation of this region.

#### Distribution.

**Figure 18.**
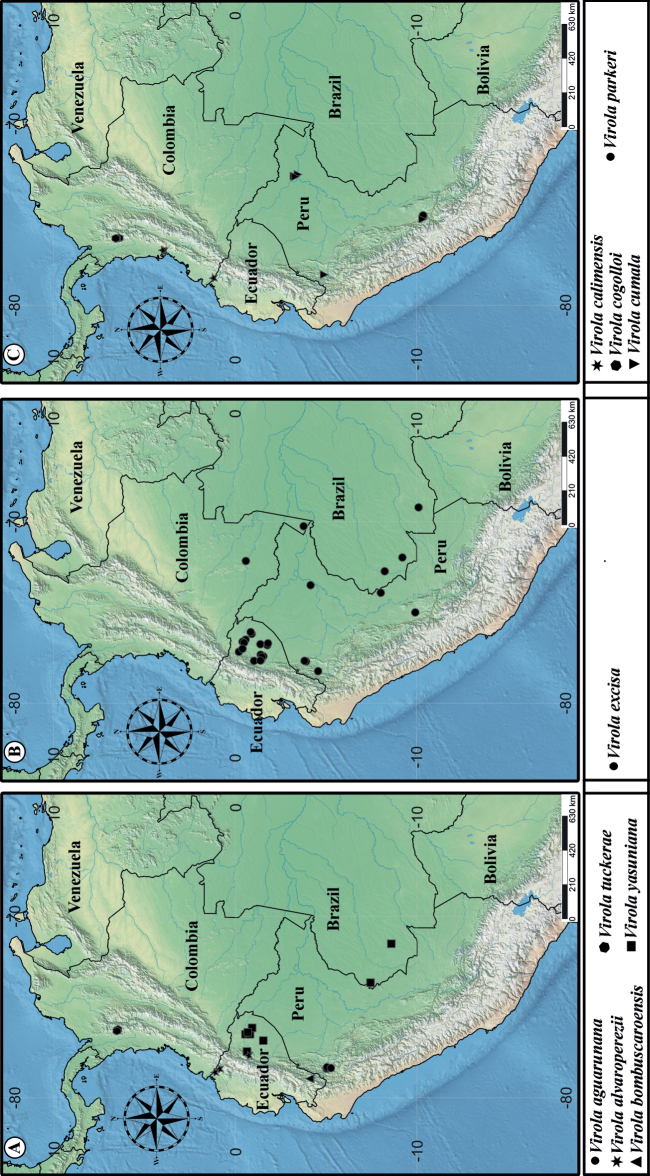
Geographic distribution of the new species of *Virola*.

*Virolayasuniana* is known from the Napo and Pastaza provinces of Ecuador, where it occurs in primary forests, on hills with red soil, or soils composed of sedimentary rocks. Is also located in Acre state of Brazil, where it grows in terra firme on poorly drained terrace (Fig. 18A). It ranges from 200–480 m in elevation, with a single collection reaching 1000 m (*C. Cerón & F. Hurtado 6560*).

#### Phenology.

Staminate flowers of *Virolayasuniana* have been collected in January, February and November, while pistillate flowers have been collected in April, May, and July. Fruits are known from February, March, June, and September.

#### Common name and uses.

Dobompapoca (Ecuador: Huaorani; *M. Aulestia & O. Gonti 1969*); guapa (Ecuador: Quichua; *C. Cerón & F. Hurtado 6560*). The wood has commercial use (*C. Cerón & F. Hurtado 6560*).

#### Preliminary Conservation Status.

*Virolayasuniana* is Vulnerable following IUCN criterion B2a. It is known from two localities, has an EOO of 117,581 km^2^, and an AOO of 32 km^2^. This species benefits from its occurrence in the Yasuní National Park of Ecuador, which experiences very low rates of deforestation (Bass et al. 2010; van der Hoek 2017), even while the broader region is experiencing land use changes (Heredia-R et al. 2021).

#### Discussion.

It is possible to confuse *Virolayasuniana* with *V.parkeri* from Peru due to their similar leaves (i.e. shape, color when dry, base, venations, and trichomes), staminate perianth that is pubescents on both surfaces, and markedly carinate fruit. Differences among the two species are summarized in Table 11.

**Table 11.** Comparison of *Virolayasuniana*, with *V.calophylla*, *V.obovata*, and *V.peruviana*. ^†^From Smith and Wodehouse (1938).

Morphological character	*V.yasuniana*	*V.calophylla*	*V.obovata*	*V.peruviana* ^†^
Leaf blade size, and base	15.7–27.5 × 4.4–6.6 cm; acute	(15–) 20–60 × 10–16 cm; (usually) deeply cordate to truncate (obtuse)	11.5–29.5 (–34) × 6.8–12.5 cm; attenuate to acute	16–35 × 6.5–10.5 cm; shallowly cordate or rounded
# of lateral veins	14–18	11–28	14–19	17–30
Long filament column	0.2–0.4 mm	0.2–0.6 mm	0.4–0.8 mm	0.4–0.6 mm
Long anthers	0.5–0.6 mm	0.4–0.5 mm	0.4–0.6 mm	1.1–1.6 mm
Fruit size, and pubescent	3.4–4.2 × 3.1–4 cm, inconspicuously pubescent	2.5–3 × 1.2–2.5 cm; tomentulose,	1.3–2.3 × 0.8–1 cm, densely pubescent	2–2.8 × 1.5–2.2 cm; glabrescent
Carina on the line of dehiscence	Markedly carinate (Fig. 6M)	Carinate (Fig. 6E)	Not carinate (Fig. 6J)	Conspicuously carinate (Fig. 6K)
Pericarp thick	1.3 and 2.3 mm (just two specimens measured)	0.5–5 mm	1–1.2 mm	0.5–1.5 mm

Herbarium specimens of *Virolayasuniana* were previously identified as *V.calophylla*, *V.obovata*, or *V.peruviana*, all of which have leaf blades with lateral veins that are well separated and stellate, sessile trichomes on abaxial leaf surface. Additionally, the new species shares fruits with a conspicuous carina with *V.peruviana*. Differences among these species are summarized in Table 10.

#### Notes.

Several collections of *Virolayasuniana* were treated as *V.obovata* in *Flora of Ecuador* (*H. Vargas & J. Cerda 678, V. Zak & S. Espinoza 4871, 5039*, *5049, 5149*; Jaramillo et al. 2004). Additionally, studied with “*aberrant fruits*” that is discussed under *V.peruviana* in *Flora of Ecuador* (Jaramillo et al. 2004) correspond with this new species (*C. Cerón & F. Hurtado 6560, M. Aulestia & G. Grefa 247, M. Aulestia & O. Gonti 1969*).

#### Specimens examined.

**Ecuador. Napo**: Parque Nacional Yasuní, carretera y Oleoducto de Maxus en construcción, Km. 20, 00°33'S, 076°30'W, 250 m, 28–30 Jul 1993 (♀ fl), *M. Aulestia & G. Grefa 247* (MO!, UPCB [n.v.]); Parque Nacional Yasuní, Carretera y oleoducto de Maxus en construcción, km 54–58, 00°48'S, 076°30'W, 250 m, 26–30 Sep 1993 (fr), *M. Aulestia & N. Andi 783* (MO!, QCNE [n.v.], UPCB [n.v.]); Reserva Etnica Huaorani, carretera y oleoducto de Maxus en construcción Km. 92–96, al norte del Río Yasun, 00°55'S, 076°09'W, 250 m, 20 March 1994 (fr), *M. Aulestia & O. Gonti 1969* (MO!, UPCB [n.v.]); Archidona, Carretera Hollín-Loreto, Km 50, comunidad Guagua Sumaco, Faldas al sur del Volcán Sumaco, 00°38'S, 077°27'W, 1000 m, 29 Apr–2 May 1989 (♀ fl), *C. Cerón & F. Hurtado 6560* (MO!, NY!); Parque Nacional Yasuni-ECY, Sendero “Napo”- 300 m, 00°40'40"S, 076°23'40"W, 200–300 m, 24 Sep 2009 (fr), *Á. Pérez & W. Santillán 4361* (MO!); Parque Nacional Yasuni-ECY, Sendero “Chorongo”- 600 m, 00°40'40"S, 076°23'40"W, 200–300 m, 16 Nov 2009 (fl bud), *Á. Pérez & W. Santillán 4402* (MO!); Loreto, 2 Km al oeste del río Tutapishco, 00°36'S, 077°22'W, 480 m, 27 Jan 1996 (♂ fl bud), *H. Vargas & J. Cerda 678* (MO!, QCA [image!], UPCB [n.v.]). **Pastaza**: Pozo petrolero “Ramírez”, 20 km al sur de la población de Curaray, [01°32'S, 076°51'W], 300 m, 21–28 Feb 1990 (♂ fl), *V. Zak & S. Espinoza 4871* (INPA [image!], MO!, NY!); ibid., 21–28 Feb 1990 (♂ fl), *V. Zak & S. Espinoza 5039* (INPA [image!], MO!, NY!, QCNE [n.v.]); ibid., 21–28 Feb 1990 (♂ fl), *V. Zak & S. Espinoza 5049* (INPA [image!], MO!, NY!). **Brazil. Acre**: Mâncio Lima, Bacia do Alto Juruá, Rio Moa, Parque Nacional da Serra do Divisor, caminho para o rio Anil, 07°26'27"S, 073°39'28"W, [not elev.], 17 Jun 1996 (imm fr), *M. Silveira et al. 1374* (NY!, UFACPZ [n.v.], UPCB [n.v.]); Mun. Tarauacá, Basin of Rio Juruá, Rio Tarauacá, right bank, Seringal Tamandaré, Colocaçao Santa Maria, Praia de Santa Maria, 08°35'12"S, 071°30'57"W, [not elev.], 18 Nov 1995 (♂ fl), *D. C. Daly et al. 8594* (NY!, UFACPZ [n.v.], UPCB [n.v.]).

##### ﻿New record

### ﻿ Virola kwatae

Taxon classificationPlantaeMagnolialesMyristicaceae

Sabatier. Adansonia ser. 3,19: 273. 1997.

9D64BA25-DF8E-5A64-B2F5-B43EF9D52015

#### Type.

**French Guiana.** Rivère Arataye, Saut Pararé, 31 Jul 1984 [♀ fl & fr], [*D.] Sabatier 931* (holotype P [barcode P00135215, image!]; isotype CAY, INPA, K [barcode K000575173, image!], MPU [barcode MPU024622, image!], NY! [NY00346038], P-3 sheets [barcodes P00135216, P00135217, P00135218, image!], US [barcode US00623540, image!]).

#### Distributions.

French Guiana and Brazil (Amapá).

#### Common name.

Gaan busi Mulumba (Paramaka; Sabatier 1997). French Guiana: yayamadou, yayamadou montagne (Créole) (Mitchell 2002).

#### Etymology.

The specific epithet alludes to the Spider monkeys, or ateles (*Atelespanisczis*), locally called kwata, who consume the fruits of this species (Sabatier 1997).

#### Note.

*Virolakwatae* was previously known from French Guiana. However, in a recent study of herbarium specimens at New York Botanical Garden, a collection with fruit made in Amapá state, Brazil was located. To our knowledge this represents the first record of this species among the Flora of Brazil.

#### Specimens examined.

**Brazil. Amapá**: Município Macapá, Rio Falsino, approx. 10 km upstream of confluence with Rio Araguari, 00°50'S, 51°45'W, 13 Dec 1984 (fr), *D. C. Daly et al. 3865* (MO [n.v.], NY!, US [image!]).

##### ﻿Nomenclatural note

### ﻿ Virola macrocarpa

Taxon classificationPlantaeMagnolialesMyristicaceae

A. C. Sm. Brittonia 2: 476. 1938.

68198C21-6920-5CE8-9FC7-2022DE5D5B89

#### Type.

**Colombia. Boyacá**: [Municipality Muzo] [El Umbo] El Humbo, ca. 130 mi N of Bogotá, alt. ca. 1100 m, 15 Mar. 1933 [fr], *A. E. Lawrence 675* (holotype: B, destroyed; lectotype, designated here: MO-2 sheets [accession 1068405, 1068404, barcodes MO-100018, MO-100024]; isolectotypes A [barcode 00039901; image!], F-2 sheets [barcodes F0360189F, F0360190F; image!], G-2 sheets [barcodes G00341083, G00341085; image!], K [barcode K000575178; image!], S [S-R-6995], US-2 sheets [barcodes US00098908, US00098909; image!]; photo of the B specimen at P [barcode 02026667; image!]).

#### Distribution.

Endemic to Colombia (Boyacá).

#### Notes.

*Virolamacrocarpa* was described by Smith based on a specimen collected by the Scottish immigrant Alexander E. Lawrance in the Andes of Boyacá, Colombia (Smith and Wodehouse 1938). In the protologue, Smith mentions that the type is deposited in the herbarium of the Botanisches Museum, Berlin-Dahlem (B), and F, M, S, US; there are additional, duplicates at A, G, K, and MO. Because the material at B was destroyed during World War II, we designate the material deposited at Missouri Botanical Garden (MO) as lectotype for this name, according to Art. 9.11 of ICN (Turland et al. 2018). This specimen is mounted on two sheets that are properly labelled as being parts of the same specimen (i.e., “Sheet 1 of 2,” “Sheet 2 of 2) according to ICN Art. 8.3 (Turland et al. 2018). One of these sheets shows the adaxial side of leaves and the infrutescence axes (MO-1068404; sheet 1 of 2), while the other (MO-1068405; sheet 2 of 2) includes fragments of both adaxial and abaxial leaf surfaces and a single dehiscent fruit that is attached, with six additional fruits inside the packet. This second sheet is annotated in Smith’s handwriting. These specimens were distributed under the name of *V.sebifera*.

The name *Virolamacrocarpa* was applied to collections from Costa Rica and Panama that now correspond to *V.allenii*, *V.amistadensis*, and *V.otobifolia*, as well some collections from Colombia and Ecuador that now correspond to *V.alvaroperezii*, *V.calimensis*, and *V.cogolloi*.

It is important to mention that, following our species concept, this name has been widely used to identify specimens, and is thus frequent in the literature and specimen databases. As circumscribed here, *V.macrocarpa* is known only from the type collection and is restricted to Colombia. We believe that the identity of following and others specimens identified as *V.macrocarpa* should be evaluated when more collections are available, including: *D. Cárdenas et al. 2772* (fr.; COL, MO); *A. Gentry & E. Rentería 24055* (fr.; COL, MO), *S. E. Hoyos-Gómez & A. Upegui 130* (fr.; COL).

### ﻿Key to *Virola* species with sessile stellate trichomes with darker centers

(*Virolaalvaroperezii* and *V.macrocarpa* are not included because staminate flowers are unknown; ^†^from Smith and Wodehouse 1938; ^$^from Carreira 1985).

**Table:** 

1a	Mature leaf blades densely pubescent on abaxial surface (Fig. 4C–G, N)	**2a**
2a	Staminate flowers with the filament column shorter than the anthers	**3a**
3a	Leaf blades widely oblong to elliptical (7.3–11 cm wide), base obtuse; staminate inflorescence broadly paniculate; staminate flowers in lax terminal fascicles, the filament column 0.4–0.6 (–0.8) mm long, anthers 0.6–1 (–1.2) mm long; fruits with persistent trichomes; pericarp ca. 3.8–4 mm thick; growing at 20–260 m elevation	***V.calimensis***
3b	Leaf blades narrowly oblong or rarely elliptical (5–7.2 [–11.7] cm wide), base cuneate; staminate inflorescence narrowly paniculate; staminate flowers in dense terminal fascicles, the filament column 0.6–0.7 (–0.9) mm long, anthers 1.2–1.6 mm long; fruits with trichomes that fall like dust; pericarp ca. 2.4 mm thick; growing at 1300–1330 m elevation	***V.tuckerae***
2b	Staminate flowers with the filament column longer than the anthers	**4a**
4a	Abaxial leaf blade surface drying silver to golden (Fig. 4E, F); perianth of staminate flowers 1.2–2.1 mm long, internal surface glabrous to glabrescent	**5a**
5a	Staminate inflorescences 12–30 cm long; ^$^pollen subtype *divergens*, type I; fruits 2.5–3 cm long (Fig. 6E); pericarp 2–5 mm thick	***V.calophylla*** ^†^
5b	Staminate inflorescences 1–4 cm long; ^$^pollen subtype *flexuosa*, type II; fruits 1.8–2.1 cm long (Fig. 6F); pericarp 0.5–0.8 mm thick	***V.calophylloidea*** ^†^
4b	Abaxial leaf blade surface drying brown or whitish-grayish (Fig. 4C, G); perianth of staminate flowers 2.8–4.1 mm long, internal surface densely pubescent	**6a**
6a	Leaf blades with cordate base; staminate inflorescences ca. 5.4 cm long, wide paniculate; staminate flowers in dense terminal fascicles, filament column with scattered trichomes (Fig. 10D); fruits covered with a conspicuous layer of trichomes that fall like dust	***V.bombuscaroensis***
6b	Leaf blades with obtuse base; staminate inflorescences 7.5–9 cm long, narrow paniculate; staminate flowers in lax terminal fascicles, filament column glabrous; fruits covered with an inconspicuous layer of persistent trichomes	***V.cogolloi***
1b	Mature leaf blades sparsely pubescent to glabrescent on abaxial surface (Fig. 4A, H–K, O)	**7a**
7a	Staminate flowers with the lobes of the perianth glabrous or almost glabrous internally	**8a**
8a	Twigs and inflorescence axes covered with appressed trichomes; fruits 2.7–3.5 long, covered by an inconspicuous layer of trichomes (Fig. 3B); pericarp 3.2–3.8 mm thick	***V.allenii***
8b	Twigs and inflorescence axes covered with erect trichomes; fruits 2–2.6 cm long, covered by a conspicuous layer of trichomes (Fig. 6I, J); pericarp 1–2.3 (–3) mm thick	**9a**
9a	Leaf blades truncate to subcordate at the base; perianth of staminate flowers without resinous punctuations when rehydrated; infructescence with peduncle ca. 1.4–3.5 (–5) cm long; fruits 1.4–1.7 cm wide, without stipe, the base truncate to obtuse	***V.excisa***
9b	Leaf blades attenuate to acute at the base; perianth of staminate flowers with resinous punctuations when rehydrated; infructescence with peduncle 0.7–1.3 cm long; fruits 0.8–1 cm wide, shortly stipitate, the base rounded	***V.obovata***
7b	Staminate flowers with the lobes of the perianth pubescent internally	**10a**
10a	Staminate inflorescence 2.2–2.5 cm long; fruits covered with a conspicuous layer of trichomes (Fig. 6A)	***V.aguarunana***
10b	Staminate inflorescence 3–9.5 cm long; fruits inconspicuously pubescent	**11a**
11a	Leaf blades (6.7–) 9.5–13.5 (–19.7) cm wide; staminate flowers with the filament column 0.9–1 mm long	***V.otobifolia***
11b	Leaf blades 4.4–9.5 (–12.5) cm wide; staminate flowers with the filament column 0.2–0.5 mm long	**12a**
12a	Leaf blades elliptical to widely elliptical; staminate flowers in lax terminal fascicles; fruits 1.7–2 cm wide, the line of dehiscence slightly carinate	***V.amistadensis***
12b	Leaf blades lanceolate to oblong; staminate flowers in dense terminal fascicles; fruits 2.8–4 cm wide, the line of dehiscence markedly carinate, like a wing	**13a**
13a	Staminate flowers with the filament column ca. 0.1 mm thick at the base, not widening at the base (i.e., straight throughout); fruits 3–3.6 × 2.8–3.1 cm, oblate, the line of dehiscence with a wing ca. 0.4 cm long	***V.parkeri***
13b	Staminate flowers with the filament column ca. 0.2–0.3 mm thick at the base, widening at the base; fruits 3.4–4.2 × 3.1–4 cm, ellipsoid to somewhat flattened, the line of dehiscence with a wing 0.4–0.7 cm long	***V.yasuniana***

### ﻿Key to distinguishing *Virolacumala* from other *Virola* species with leaves with numerous, close lateral veins

(Adapted from Smith and Wodehouse 1938 and Rodrigues 1980; ^‡^material from Peru quoted here under notes of *V.cumala*).

**Table:** 

1a	Adaxial surface of mature leaf blades pubescent	**2a**
2a	Leaf blades 25–60 cm long; staminate perianth submembranous; mature fruits covered by a conspicuous layer of trichomes (Fig. 12E)	***V.decorticans***
2b	Leaf blades 5–22 (–25.5) cm long; staminate perianth carnose to subcarnose; mature fruits without conspicuous layer of trichomes (glabrescent or densely tomentose to glabrate) (Fig. 12A–C, F, G, I)	**3a**
3a	Leaf blades with 28–32 lateral veins; staminate inflorescences to 8.5 cm long; staminate flowers with perianth 2–3 mm long; filament column 1.3–1.5 mm long; deciduous during blossom	***V.chrysocarpa***
3b	Leaf blades with 24–58 lateral veins; staminate inflorescences to 14 cm long; staminate flowers with perianth ca. 1–1.5 mm long; filament column 0.3–0.4 mm long; evergreen	***V.guggenheimii***
1b	Adaxial surface of mature leaf blades glabrous	**4a**
4a	Leaf blades with 16–38 lateral veins	**5a**
5a	Leaf blades on abaxial side densely tomentose, with pediculate, dendritic trichomes; staminate flowers with perianth 2–2.5 mm long, carnose; androecium 1.2–1.6 mm long; fruits on the line of dehiscence smooth or slightly carinate, apex obtuse (Fig. 12F)	***V.koschnyi***
5b	Leaf blades on abaxial side tomentulose to puberulent, with sessile or shortly pediculate, stellate trichomes; staminate flowers with perianth 1.2–1.8 mm long, membranous to submembranous; androecium 0.8–0.9 mm long; fruits on the line of dehiscence carinate, apex apiculate to acute	**6a**
6a	Leaf blades with 40–50 lateral veins; staminate inflorescence with 10–15 flowers per fascicle	***V.flexuosa***
6b	Leaf blades with 30–38 lateral veins; staminate inflorescence with 50–100 flowers per fascicle	***V.minutiflora***
4b	Leaf blades with (30–) 40–60 lateral veins	**7a**
7a	External surface of staminate perianth almost glabrous (pubescent only at base and apex)	***V.multinervia***
7b	External surface of staminate perianth uniformly pubescent (not described for *V.multicostata*)	**8a**
8a	Staminate flowers with perianth 2–3 mm long; fruits 3–5.7 cm long, conspicuously pubescent, always present; pericarp 3–6 mm thick	**9a**
9a	Leaf blades with 49–65 lateral veins; staminate flowers with filament column 0.5–0.7 mm long; fruits rounded to ellipsoid, covered by a conspicuous layer of trichomes (1–2.3 mm thick), obtuse at apex (Fig. 12D)	***V.cumala***
9b	Leaf blades with (32–) 40–50 lateral veins; staminate flowers with filament column 0.9–1.3 mm long; fruits ovoid-ellipsoid, covered by an inconspicuous layer of trichomes, acuminate to rostrate at apex (Fig. 12G)	***V.megacarpa***
8b	Staminate flowers with perianth 1–1.5 mm long (not described in *V.polyneura*); fruits 1.4–3 (–3.5) cm long, glabrous or densely tomentellous (if tomentellous, cauduous trichomes soon falling and leaving surface completely glabrous; if conspicuously pubescent, the layer of trichomes falling easily to the touch [*V.crebrinervia*]); pericarp 1–3 (–5) mm thick	**10a**
10a	Abaxial surface of leaf blades glabrous to glabrescent	**11a**
11a	Leaf blades 10–18 (–29) × 2.2–3 (–6) cm, narrowly oblong-lanceolate; fruits 2–3.5 × 1.8–2.5 cm, tomentose (Fig. 12C); pericarp 2–5 mm thick	***V.crebrinervia***
11b	Leaf blades 20–28 × 4–10 cm, elliptic or oblong-elliptic; fruits 2.1–2.3 × 1.5–1.8 cm^‡^, glabrous to glabrescent (Fig. 12I); pericarp 1.3–1.5 mm thick^‡^	***V.multicostata***
10b	Abaxial surface of leaf pubescent	**12a**
12a	Leaf blades 10–42 × 3.5–12.5 cm, abaxial surface with sessile trichomes; deciduous in flower	***V.caducifolia***
12b	Leaf blades 5.5–11 × 4–8.5 cm, abaxial surface with pediculate trichomes; evergreen	***V.polyneura***

## Supplementary Material

XML Treatment for Virola aguarunana

XML Treatment for Virola alvaroperezii

XML Treatment for Virola bombuscaroensis

XML Treatment for Virola calimensis

XML Treatment for Virola cogolloi

XML Treatment for Virola cumala

XML Treatment for Virola excisa

XML Treatment for Virola parkeri

XML Treatment for Virola tuckerae

XML Treatment for Virola yasuniana

XML Treatment for Virola kwatae

XML Treatment for Virola macrocarpa
